# Novel Sulfur‐Containing Polyurethanes using 5‐(Chloromethyl)Furfural as a Renewable Building Block

**DOI:** 10.1002/cssc.202500888

**Published:** 2025-08-07

**Authors:** Jorge Andrés Mora Vargas, Jéssica Ribeiro da Silva, Ana Clara Lancarovici Alves, Germán Darío Gómez Higuita, Antonio José Felix Carvalho, Antonio C. B. Burtoloso

**Affiliations:** ^1^ Institute of Chemistry of São Carlos University of São Paulo São Carlos São Paulo CEP 13560‐970 Brazil; ^2^ São Carlos School of Engineering University of São Paulo São Carlos São Paulo CEP 13560‐970 Brazil; ^3^ São Carlos Institute of Physics University of São Paulo São Carlos São Paulo CEP 13560‐970 Brazil

**Keywords:** 5‐(chloromethyl)furfural, biomass‐derived monomers, chemical degradation, furandiacylazide, polyurethanes

## Abstract

The development of novel polymeric materials with unique properties and applications across various industries, driven by biomass‐based molecular platforms, has experienced remarkable growth. Among these, 5‐(chloromethyl)furfural (CMF) has emerged as a promising precursor to produce biomass‐derived monomers and innovative chemical products. Herein, an efficient method is presented for synthesizing novel biomass‐derived diols, achieved in very good yields by reacting CMF with various dithiols, followed by reduction of the resulting dialdehydes. Subsequently, 12 new polyurethanes are synthesized via polyaddition of these diols with commercially available diisocyanates, using an organic base as a catalyst. The resulting polymers exhibit molecular weights ranging from 1.7 to 57.7 kDa, glass transition temperatures between 22 and 120 °C, and degradation temperatures (Td5%) exceeding 170 °C. Additionally, a nearly fully biomass‐derived polymer is synthesized through the reaction of furandiazylazide (FDAz) with one of the obtained diols, via Curtius rearrangement. Recognizing the importance of developing innovative polymers alongside efficient chemical degradation and recycling methods, a synthesized polyurethane is also subjected to chemical degradation studies.

## Graphical abstract

A novel methodology is presented for synthesizing biomass‐derived diols using 5‐(chloromethyl)furfural, thiols, and mild reaction conditions. These innovative diols are subsequently utilized in the production of new polyurethanes via polyaddition with both commercially available and biomass‐derived diisocyanates. Furthermore, a method is presented for the acid‐catalyzed degradation of these polyurethanes.

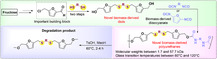

## 1 Introduction

Polyurethanes (PUs) represent an interesting class of polymeric materials extensively employed in the chemical and pharmaceutical industries. They find applications in foams, coatings, elastomers, and more.^[^ ^1^, ^2^ ^]^ Conventionally, PUs have been produced through polyaddition reactions involving diols and diisocyanates. However, these raw materials are predominantly derived from petroleum‐based sources.^[^ ^3^, ^4^ ^]^ In response to environmental concerns, there has been a growing emphasis on developing eco‐friendly methods for synthesizing polyurethanes using natural resources,^[^ ^5^, ^6^, ^7^, ^8^ ^]^ as well as easy ways for its recycling or degradation. An interesting approach in this direction is the chemical conversion of carbohydrates, mainly C_5_ and C_6_ sugars. The use of carbohydrates has emerged as a significant advantage for generating new and known macromolecules, biofuels, renewable monomers for the polymer manufacturing industry, and other chemicals.^[^ ^9^, ^10^, ^11^ ^]^ For example, different polymer feedstocks such as 5‐hydroxymethylfurfural (HMF),^[^ ^12^, ^13^ ^]^ 5‐chloromethylfurfural (CMF),^[^ ^10^ ^]^ furandiazylazide (FDAz),^[^ ^14^ ^]^ and 2,5‐furandicarboxylic acid (FDCA)^[^ ^15^, ^16^ ^]^ have been obtained and employed as key intermediates for the synthesis of different biomass‐derived monomers and polymers containing a furan ring. Among these feedstocks, HMF has gained attention as a raw material for the synthesis of these added‐value compounds. For example, Wilson and Chen reported the synthesis of various rigid difuranic C_11_ diols by the cross‐coupling reaction between HMF and furfural, catalyzed by organic *N*‐heterocyclic carbenes, and their application in the synthesis of polyesters and polyurethanes.^[^ ^17^ ^]^ Like this example, different HMF‐based monomers have been reported.^[^ ^18^, ^19^, ^20^, ^21^ ^]^

Interestingly, 5‐(chloromethyl)furfural (CMF) has emerged as an important and stable alternative to the use of HMF. It can be obtained in good yields by treating carbohydrates, cellulose, or raw biomass with commercial‐grade hydrochloric acid (HCl) in the presence of an organic solvent such as dichloromethane, chloroform, and dichloroethane in a biphasic reaction.^[^ ^22^, ^23^, ^24^, ^25^ ^]^ The furan ring, aldehyde group, and leaving group in CMF make it highly versatile for the synthesis of a wide range of target molecules, including monomers, biofuels, agrochemicals, pharmaceuticals, and other chemical compounds.^[^ ^11^, ^26^, ^27^ ^]^ One of the most important industrial applications of CMF is its transformation into *p‐*xylene, which is a key intermediate employed in the production of terephthalic acid (a monomer employed to obtain terephthalate‐derived polymers).^[^ ^28^, ^29^, ^30^ ^]^ In polymer synthesis, CMF was employed for the synthesis of hydroxyl functional furan polymers using a Barbier polycondensation reaction catalyzed by zinc, a inexpensive and mild metal reagent.^[^ ^31^ ^]^

**Scheme 1:**
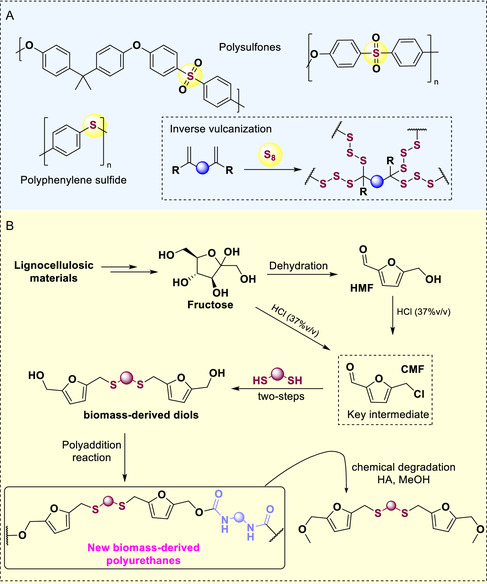
A) Examples of commercial sulfur‐containing polymers. B) This work: synthesis of new polymers from biomass‐derived monomers.

Sulfur‐based polymers have emerged as valuable materials, exhibiting a wide range of mechanical, chemical, physical, and thermal properties that are suitable for various industrial applications. Notably, poly(thiocarbonate), poly(thioesters), and poly(sulfites) are among the most significant representatives (**Scheme** **1**A). However, the synthesis and development of novel, complex sulfur‐based polymers present several challenges, including the complexity and instability of the monomer structures, low polymer solubility, and a tendency to produce disordered structures, among other issues.^[^ ^32^, ^33^, ^34^, ^35^ ^]^ More recently, different research groups have disclosed the inverse vulcanization of vegetable oils with elemental sulfur to synthesize this class of polymeric material. However, the fact that these oils combine different chemical structures in their composition makes the polymerization process difficult.^[^ ^36^, ^37^, ^38^, ^39^ ^]^

Herein, we outline a strategy for synthesizing novel sulfur‐containing polyurethanes (Scheme 1B) using new biomass‐derived diols and commercially available diisocyanates. The diols were produced by reacting CMF with different dithiols under mild reaction conditions, followed by the reduction of the resulting dialdehyde. These novel sulfur‐containing polymers could also be degraded under acid media yielding chemicals with potential for reusability. Moreover, a bio‐based diisocyanate was prepared and employed in the synthesis of a totally biomass‐derived polymer.

## 2 Results and Discussion

In this work, we started our study by evaluating the best conditions for the disubstitution reaction between CMF (**1**) and dithiols (**2**) (**Table** **1**). For this screening, we decided to use propane‐1,3‐dithiol (**2a**) as the model substrate. Initially, **2a** (1 mmol), in a 0.1 mol L^−1^ solution in acetonitrile (ACN), was mixed with 2 equiv. of **1** and 1.5 equivalents of triethylamine. Under these conditions, none of the expected substitution products was observed. To increase the reactivity of CMF (**1**) in the nucleophilic substitution reaction, 20 mol% (0.2 equiv.) of tetrabutylammonium iodide (nBu_4_NI) was employed. Although **1** was totally consumed, a mixture of the di‐ and monoalkylated **3a** and **3a′** was obtained. In this case, the undesired monoalkylated product **3a′** was obtained as the major product (85% yield) (Entry 1). Increasing the amount of base and nBu_4_NI to 3.0 equiv. and 0.4 equiv. provided **3a** and **3a′** in 39% and 41% yields, respectively (Entry 2). Using 3.0 equiv. of **1** under the previous conditions, the desired dialkylated product **3a** was obtained in 70% yield, with no formation of **3a′** (Entry 3). The concentration of the reaction was also evaluated. Increasing it to 0.2 mol L^−1^ and 0.4 mol L^−1^ boosted the reaction yield to 80% and 96% (Entries 4 and 5, respectively). However, increasing the concentration to 2 mol L^−1^ caused a significant reduction in the yield (Entry 6). Using tetrahydrofuran (THF), 2‐methyl tetrahydrofuran (2‐Me‐THF), or ethyl acetate (AcOEt) as solvents, the desired product was obtained in excellent yields as well (Entries 7 to 9). These solvents, especially 2‐Me‐THF, are considered “recommended” by Sanofi's Solvent Selection Guide.^[^ ^40^ ^]^ Finally, based on previous works for monoalkylation reactions,^[^ ^41^ ^]^ sodium iodide (NaI) was also employed as an additive; however, **3a** was not observed or obtained in poor yield, even using ACN as the solvent (Entries 10 and 11).

**Table 1:** Optimization of the reaction conditions.

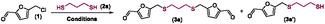						
Entrya)	CMF [equiv.]	Additive [equiv.]	Solvent	C [M]d)	3a [%]	3a′ [%]
**1** b)	2.0	nBu_4_NI (0.2)	ACN	0.1	6	86
**2** c)	2.0	nBu_4_NI (0.4)	ACN	0.1	39	41
**3** c)	3.0	nBu_4_NI (0.4)	ACN	0.1	70	–
**4** c)	3.0	nBu_4_NI (0.4)	ACN	0.2	80	–
**5** c)	3.0	nBu_4_NI (0.4)	ACN	0.4	96	**–**
**6** c)	3.0	nBu_4_NI (0.4)	ACN	2.0	48	16
**7** c)	3.0	nBu_4_NI (0.4)	THF	0.4	95	–
**8** c)	3.0	nBu_4_NI (0.4)	EtOAc	0.4	93	–
**9** c)	**3.0**	**nBu_4_NI (0.4)**	**2‐Me‐THF**	**0.4**	**98**	–
**10** c)	3.0	NaI (0.4)	2‐Me‐THF	0.4	5	–
**11** c)	3.0	NaI (0.4)	ACN	0.4	–	–

a) Yields refer to Isolated products. Scale = 1 mmol of **2a**.

b) Equivalent number of Et_3_N = 1.5

c) Equivalent number of Et_3_N = 3.0. All reactions were carried out at room temperature for 20 h.

d) The concentration is based on compound **2a.**

**Scheme 2:**
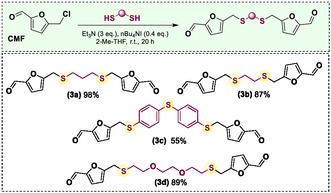
Dialdehydes obtained by the reaction between CMF and dithiols. Scale = 1 mmol of dithiol. Isolated yields.

With the optimal conditions established, we synthesized three new dialdehydes (**3**) using different dithiols, as depicted in **Scheme** **2**. Using ethane‐1,2‐dithiol as the starting material, the corresponding product **3b** was obtained in 89% yield. The reaction was also effective in the presence of less nucleophilic thiols, yielding products **3c** and **3d** in moderate to good yields. It is noteworthy that the excess of CMF employed in the reaction can be recovered using chromatography, during the purification of the desired dialdehydes **3** (yielding between 40% and 48%).

**Scheme 3:**
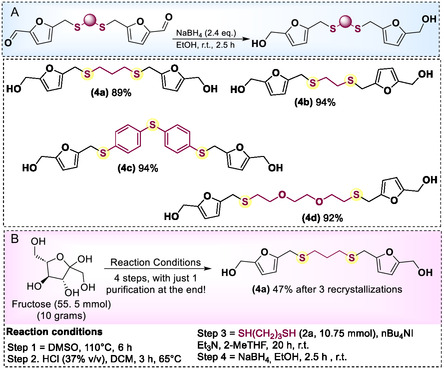
A) Diols (**4**) obtained by the reduction of **3**. B) Synthesis of the diol **4a** from fructose employing only extraction and recrystallization as purification methods.

It is important to note that although the mono‐alkylation of alcohols and thiols with CMF is already described in literature, to the best of our knowledge, this is the first example for the di‐alkylation version. The development of this approach enabled access to unprecedented furan‐based structures, representing a new class of renewable monomers. After confirming the structure of each dialdehyde **3**, using ^1^H and^13^C{^1^H} nuclear magnetic resonance (NMR) analysis (see Supporting Information), the corresponding diols were obtained using sodium borohydride as the reducing agent. Compounds **4a–d** were prepared in 89–94% yields (**Scheme** **3**A). One of the key points in sustainability is making processes industrially translatable. Considering that, we decided to obtain these bio‐based diol monomers in a larger scale and without using chromatographic purification for each reaction step. Starting from fructose, employing only extractions and making the purification only in the last step, by recrystallization, diol **4a** could be obtained in 47% yield from **2a** (Scheme 3B, for details, see Supporting Information).

Additionally, and with the aim of identifying a better approach for the reduction step, we screened various hydrogen sources, including formic acid, potassium formate (HCOOK), molecular hydrogen, and isopropanol. For this, dialdehyde **3b** was used as the model substrate, with the results summarized in **Table** **2**. Treatment of **3b** with H_2_ (using a balloon) or HCOOK in the presence of 10 mol% Pd/C did not yield the diol **4b** (Entries 1 and 2).^[^ ^42^ ^]^ Similar results were obtained with Zn dust and ammonium chloride (Entry 3).^[^ ^43^ ^]^ Using formic acid as the hydrogen source, along with palladium acetate (Pd(OAc)_2_) and tricyclohexylphosphine (Cy_3_P),^[^ ^44^ ^]^ produced low yields of **4b** (Entry 4). Interestingly, The Meerwein‐Ponndorf‐Verley reaction, an interesting alternative for reducing biomass‐derived compounds,^[^ ^45^, ^46^, ^47^ ^]^ was explored using the inexpensive aluminum isopropoxide (Al(*i*‐PrO)_3_) as the catalyst.^[^ ^48^, ^49^ ^]^ This approach furnished **4b** in excellent yields, even on a larger scale (up to 97%, Entries 5–7).

**Table 2:** Reaction conditions tested for the aldehyde reduction.

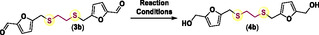		
Entrya)	Reaction conditions	4b [%]
**1** b)	Pd/C (10 mol%), H_2_, K_2_CO_3_ (20 mol%), EtOH/H_2_O, 2 h, 25 °C	N.R.
**2** b)	Pd/C (10 mol%), HCOOK (3 equiv.), EtOH/H_2_O, 24 h, 25 °C.	N.R.
**3** b)	Zn (10 equiv.), NH_4_Cl (8 M), THF, 15 h	N.R.
**4** b)	Pd(OAc)_2_ (10 mol%), Cy_3_P (14 mol%), H_2_O (20 mol%), formic acid (8 equiv.), dioxane, 18 h, 90 °C	30
**5** b)	Al(*i*‐PrO)_3_ (1.2 equiv.), *i*‐PrOH/THF (7:2), 1 h, 85 °C.	68
**6** b)	**Al(** ***i*** **‐PrO)**_**3**_ **(1.2 equiv.),** ***i*** **‐PrOH/THF (7:2), 1 h, 85 °C, then HCl**	**97**
**7** c)	Al(*i*‐PrO)_3_ (1.2 equiv.), *i*‐PrOH/THF (7:2), 1 h, 85 °C, then HCl	87

a) Isolated yield.

b) Scale of the reaction = 1 mmol of **3b**.

c) Scale of the reaction = 3.1 mmol of **3b** (1.0 g of starting material).

Having satisfactorily obtained the biomass‐derived diols, we proceeded to synthesize new polyurethanes (PUs). As is known, the synthesis of this type of polymeric material requires the use of some catalysts to achieve high molecular weight, with organotin‐based catalysts being traditionally used. However, their utilization comes with certain drawbacks, including challenges in removing the catalyst from PUs, which can elevate the purification costs. Additionally, concerns arise regarding the toxicity of the catalyst and other associated issues. To solve these drawbacks, organocatalysts have emerged as an interesting alternative to avoid organotin‐based catalysts. Among these, organic bases, including tertiary amines, amidines, *N*‐heterocycle carbenes, and guanidines, have gained widespread use, due to their high selectivity, relatively fast reaction rates, and excellent tolerance for different functional groups.^[^ ^50^, ^51^ ^]^

**Scheme 4:**
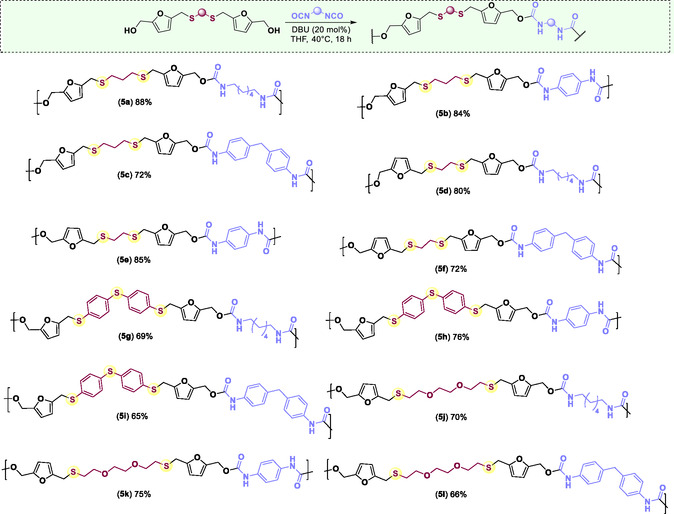
Chemical structures of the obtained biomass‐derived PUs. Reaction condition = 0.5 mmol of diisocyanate, 0.5 mmol of diol, 20 mol% of DBU, and THF as the solvent. The reaction was carried out at 40 °C for 18 h. The yield corresponds to the mass of isolated polymer after methanol wash as a percentage of the anticipated total mass, assuming 100% conversion of the reagents.

Accordingly, we evaluated the polyaddition reaction between the synthesized diols (**4**) and different commercially available diisocyanates, using 1,8‐diazabicyclo[5.4.0]undec‐7‐ene (DBU) as the catalyst (**Scheme** **4**). Twelve new PUs were synthesized using this protocol, and their proposed structures were supported by infrared spectroscopy and ^1^H and^13^C{^1^ H} NMR (see Supporting Information). Infrared spectra showed the N‐H absorption band (υ_N‐H_) between 3307 and 3288 cm^−1^. Additionally, a strong absorption band was observed at about 1700 cm^−1^, which is characteristic of the stretching vibrations of C=O in the urethane group. The stretching vibrations of the C—N bond and the bending vibration of the N—H bond were observed around 1537 cm^−1^ and 1533 cm^−1^, respectively. In the ^1^H NMR spectra, the chemical shift of the hydrogen on the urethane group (‐NHCOO‐) was observed at either 9.6 ppm or 7.2 ppm. Two peaks with chemical shifts of about 6.4 ppm (*d*, *J *= 3.1 Hz, 2 H) and 6.2 ppm (*d*, *J *= 3.2 Hz, 2 H) were observed and assigned to the hydrogens on the furan ring. Hydrogens on the methylene groups bonded to the oxygen and sulfur atoms were observed at about 5.0 ppm and 3.7 ppm, respectively. In the ^13^C NMR spectra, the carbonyl peak was observed between 155 ppm and 153 ppm, and the quaternary carbons on the furan ring were verified at 152 ppm and 149 ppm. Olefinic carbons on the furan ring were also observed with a chemical shift between 111 ppm and 108 ppm. In addition, all the signals corresponding to the hydrogen and carbon atoms on the aliphatic or aromatic substituents were observed by ^1^H and ^13^C NMR (see Supporting Information).

**Figure 1:**
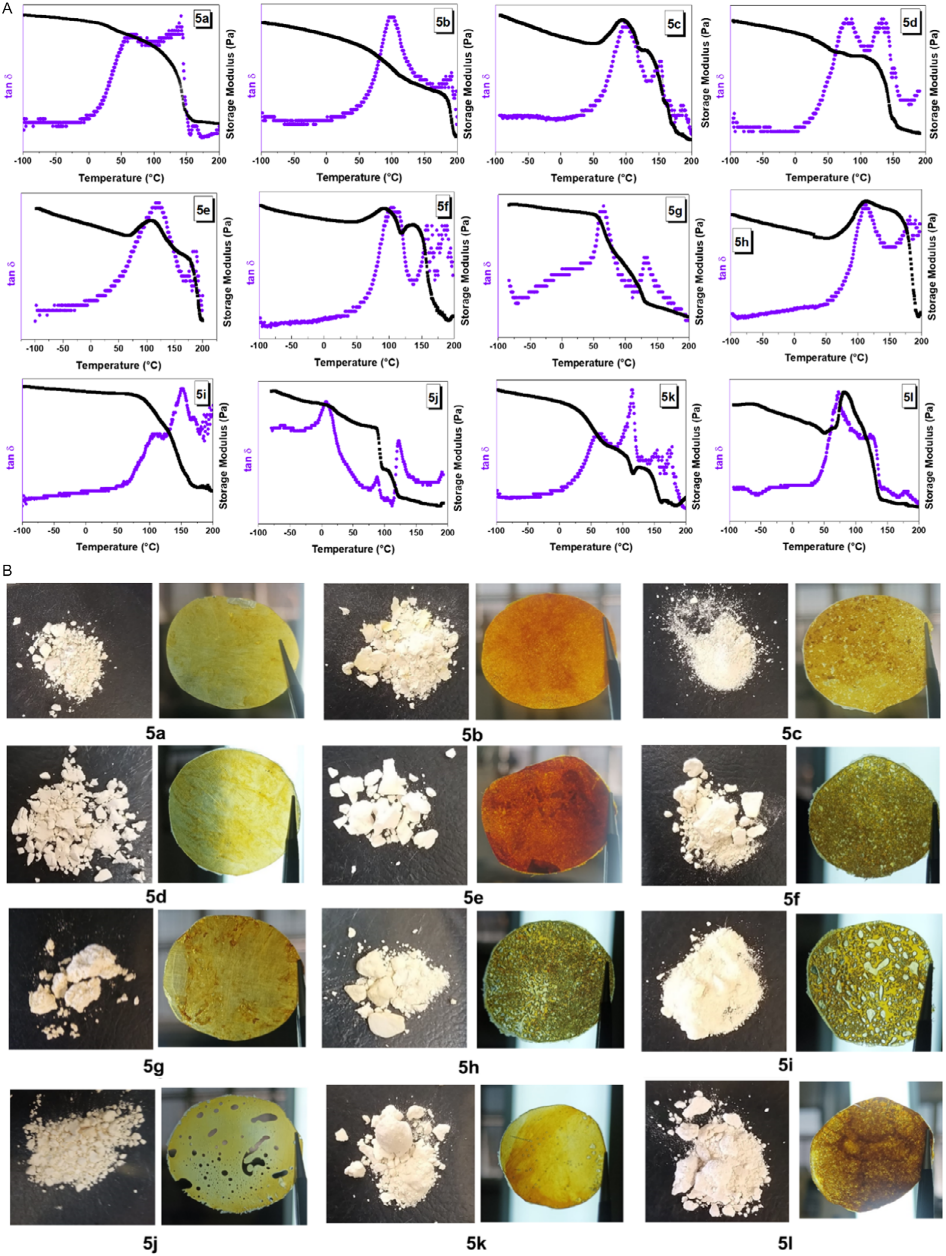
A) DMTA curves for the synthesized polyurethanes derived from diols (**4**) and commercially available diisocyanates. B) Photographs of the obtained polymer after precipitation with methanol and the resulting films. PU **5g** was pressed at a temperature of 145 °C. PUs **5a**, **5b**, **5d**, **5k**, and **5l** were pressed at 150 °C. PUs **5c**, **5f**, and **5i** were pressed at 155 °C, and PUs **5j**, **5h**, and **5e** were pressed at 160 °C.

The thermal properties of each PU were also investigated via dynamic mechanical thermal analysis (DMTA) (**Figure** **1**) and thermogravimetric analysis (TGA) (See Supporting Information). The grass transition temperatures (*T*_g_) of each PU were determined by DMTA. As shown in Table 2, *T*_g_ of the PUs ranged from 60 to 120 °C, depending on the chemical structure of the polyurethane. Comparatively, PUs derived from hexamethylene diisocyanate (HMDI) with diols **4a**, **4b**, and **4c** (**5a**, **5d**, and **5g**) exhibit lower *T*_*g*_, those PUs derived from 1,4‐phenylene diisocyanate (PPDI) and 4,4′‐methylenebis‐(phenyl isocyanate) (MDI). PUs **5a**, **5g**, and **5k** showed the lowest *T*_*g*_ values (between 60 and 66 °C).

The TGA curves indicated three thermal degradation events, influenced by the presence of aliphatic or aromatic groups in PUs. For example, PUs synthesized from HMDI showed the first and second events with the maximum degradation rates occurring at temperatures between 180 °C and 390 °C, with weight losses between 61 and 75%, and the third degradation step at temperatures between 370 and 552 °C with weight losses between 12 and 16%. These degradation steps are associated with urethane bonds degradation (around 200 °C), alcohols, isocyanates, and furan rings scission. The onset thermal decomposition temperatures (T_d_ at 5% weight loss) are also shown in Table 2. Linear PUs (**5a**, **5d**, **5g**, and **5j**) showed T_d_ at 5% around 245 °C. However, aromatic groups in the diisocyanate and diol resulted in materials with lower T_d_ at 5% (between 173 and 200 °C). Temperatures corresponding to 15 wt% and 50 wt% of degradation were used to compare the thermal stability of the PUs. *T*_d50%_ was greater than 300 °C in all cases. TGA curves also showed that the PUs did not completely decompose even at temperatures above 900 °C, resulting in a stable carbonaceous material as presented in Table 2. This behavior was also observed in other PUs containing furan rings.^[^ ^17^, ^52^ ^]^ Finally, the molecular weight (M_W_) of each PU was determined using static light scattering (**Table** **3**). We obtained polyurethanes with molecular weights ranging between 1.7 and 57.7 kDa. Notably, similar yields and molecular weights were achieved by reducing the reaction time by 0.5 h, instead of 18 h (see Supporting Information).

**Table 3:** PU thermal properties and molecular weight.

Polymer	Thermal degradationa)				T_g_ [°C]b)	M_W_ [kDa]c)	DPd)
	T_5%_ [°C]	T_15%_ [°C]	T_50%_ [°C]	Char [%]			
**5a**	245	267	313	16	66	4.5	9
**5b**	178	218	322	20	98	2.2	5
**5c**	179	202	310	24	99	1.7	3
**5d**	244	263	301	13	78	2.6	5
**5e**	183	206	364	39	120	12.3	26
**5f**	184	220	313	26	105	2.8	5
**5g**	242	267	315	23	65	6.4	10
**5h**	189	231	360	32	110	5.2	8
**5i**	200	251	436	38	105	3.3	5
**5j**	243	274	315	12	88	57.7	101
**5k**	173	232	313	23	60	2.2	4
**5l**	173	234	313	23	74	3.0	5
**5m**	203	260	313	25	22	27.4	57

a) Determined using the TGA curves.

b) Determined from the DMTA.

c) Determined using the Debye plot obtained by SLS. The intercept of the plot gives 1/M_W_.

d) Degree of polymerization.

**Figure 2:**
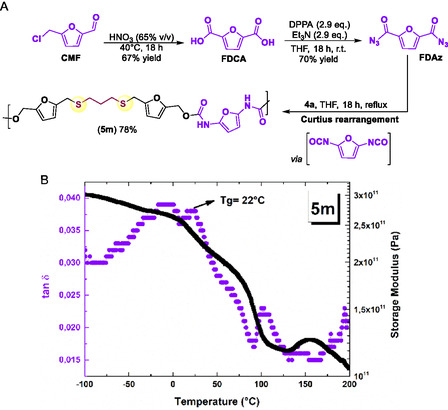
A) Synthetic route employed to obtain **5m**. The yield of **5m** corresponds to the mass of isolated polymer after methanol wash as a percentage of the anticipated total mass, assuming 100% conversion of the reagents. B) DMTA curve of the synthesized polyurethane.

After successfully investigating the synthesis of novel polyurethanes through the polyaddition reaction, involving the new bio‐based diols (**4**) and commercially available diisocyanates, our focus shifted to synthesizing a polyurethane where both monomers were, in major, derived from biomass. To achieve this, we employed furandiacylazide (FDAz), an interesting biomass derivative.^[^ ^14^ ^]^ FDAz can be synthesized in good yield from 2,5‐furandicarboxylic acid (FDCA) using diphenyl phosphoryl azide (DPPA, a stable, nonexplosive azide source) and triethylamine (Et_3_N).^[^ ^14^ ^]^ Our strategy began with the synthesis of FDCA by the oxidation of CMF using nitric acid.^[^ ^53^ ^]^ Subsequently, FDCA was reacted with DPPA and ET_3_N in THF at room temperature, producing the desired FDAz. Through a Curtius rearrangement, FDAz provided in situ the diisocyanate as the key intermediate. This intermediate then reacted with diol **4a**, yielding the polyurethane **5m**, which showed a molecular weight of 27.4 kDa (determined by SLS) (**Figure** **2**A). The proposed structure of **5m** was supported by infrared spectroscopy and ^1^H and ^13^C{^1^H} NMR (see Supporting Information).

The thermal properties of this PU were also investigated by TGA. A thermal event was observed in the TGA curve with the maximum degradation occurring at temperatures between 180 and 352 °C, with weight loss *c.a.* 56%. Additionally, the onset thermal decomposition temperature (*T*_d5%_) was observed at 203 °C (Table 2). *T*_*g*_ of **5m** was observed by DMTA being 22 °C (Figure 2B).

**Scheme 5:**
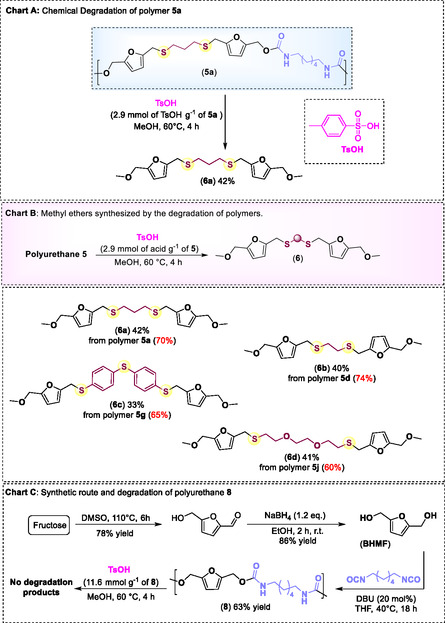
Chart A) Chemical degradation of polyurethane **5a** using *p*‐toluenesulfonic acid monohydrate (TsOH) and methanol. Chart B) Methyl ethers (**6**) obtained by treating different polyurethanes with TsOH (2.9 mmol of acid per g of polymer) in methanol at 60 °C. Values in red belong to the percentage of polymer degradation based on recovered material. Chart C) Synthetic route employed for the synthesis of **8**.

Understanding the significance of chemical degradation and recycling methods for polymers, we proceeded to investigate the degradation of synthesized polyurethanes under acidic conditions. We began by degrading polyurethane **5a** using *p*‐toluenesulfonic acid monohydrate (TsOH) and methanol at 60 °C for 2 h. Initially, we employed 2.9 mmol of TsOH per gram of polymer, which resulted in 32% yield of degradation product **6a** (**Scheme** **5**), an ether‐derived diol, with the polymer exhibiting 30% degradation based on the recovered material. By increasing the acid concentration to 5.8 mmol per gram of polymer, we observed an increase in polymer degradation to ≈51%. However, this resulted in a decrease in the yield of product **6a** (20% yield). Repeating the initial reaction condition (2.9 mmol of TsOH per gram of polymer), but increasing the reaction time to 4 h, we observed a 70% degradation of the polyurethane **5a**, with **6a** as the only product with 42% yield (Scheme 5, Chart A). Using *p*‐nitrobenzoic or acetic acid (even at a concentration of 11.6 mmol of acid per gram of polymer) did not result in any degradation, as the polymer remained intact and fully recovered. Finally, treating different polyurethanes with *p*‐toluenesulfonic acid (2.9 mmol of acid per gram of polymer) yielded the corresponding methyl ether (**6a**–**6d**) in moderate yields (Scheme 5, Chart B).

In order to compare the acid degradation of the *sulfur*‐containing polymers with the *oxygen*‐containing ones, polyurethane **8** was synthesized. Polymer **8** was synthesized using HMDI and 2,5‐bis(hydroxymethyl)furan (BHMF) as the starting materials, with DBU as the catalyst. The polymer was obtained in 63% yield based on the mass of the isolated polymer (Scheme 5, Chart C). Degradation studies were conducted using 11.6 mmol of TsOH per gram of polymer, resulting in only 13% degradation, as determined by the recovered material. The remaining polymer was isolated as a black solid, and no degradation products were observed (monitored by TLC), even when the TsOH concentration was further reduced (for details of the methodology, see Supporting Information). These results indicate that introducing sulfur atoms between the furan rings not only provides new properties for the synthesized polymers but also enables their easy degradation into value‐added products under mild reaction conditions (the extent to which this is an advantage or otherwise depends on the balance between biodegradability and serviceability).

## 3 Conclusion

As a conclusion, a new way to make bio‐based diols, using CMF as an important and promising biomass‐derived chemical platform, is described. In just two steps, several new diols were prepared in global yields of up to 87% and using 2‐Me‐THF as a green solvent. Larger‐scale preparations, starting directly from fructose, without purification of any chemical intermediate were also accomplished. This provided the diol in 47% global yield after four sequential transformations and a single purification in the last step by recrystallization. All the diols were employed in the preparation of twelve (12) new polyurethanes, each with different properties and characteristics. A nearly fully bio‐based polyurethane was also prepared, employing a biomass‐derived diisocyanate. Finally, we could also demonstrate the chemical degradation of these sulfur‐derived PUs, comparing it with similar oxygenated polymers.

## Conflict of Interest

The authors declare no conflict of interest.

## Supporting information

Supplementary Material

## Data Availability

The data that support the findings of this study are available in the supplementary material of this article.
