# Supplementary material for: Novel Sulfur‐Containing Polyurethanes using 5‐(Chloromethyl)Furfural as a Renewable Building Block
Source: ChemSusChem. 2025 Aug 7;18(18):e202500888. doi: 10.1002/cssc.202500888 (PMC12456384; doi:10.1002/cssc.202500888)

Supporting information

Jorge Andrés Mora Vargas,[a] Jéssica Ribeiro da Silva,[a] Ana Clara Lancarovici Alves,[b] Germán Darío Gómez Higuita,[c] Antonio José Felix Carvalho,[b] and Antonio C. B. Burtoloso*[a]

[a] Institute of Chemistry of São Carlos, University of São Paulo, São Carlos, São Paulo, CEP 13560-970, Brazil

[b] São Carlos School of Engineering, University of São Paulo, São Carlos, São Paulo, CEP 13560-970, Brazil

[c] São Carlos Institute of Physics, University of São Paulo, São Carlos, São Paulo, CEP 13560-970, Brazil

*E-mail: antonio@iqsc.usp.br

Table of contents

1.	General Information.	2
2.	Synthesis of new biomass-derived diols from 5-(chloromethyl)furfural.	2
2.1.	Synthesis of 5-(chloromethyl)furfural (CMF) from fructose (two-step)	2
2.2.	Synthesis of dialdehydes (3) by substitution reaction between CMF and dithiols	4
2.2.1.	Copies of the NMR spectra of dialdehydes (3).	5
2.3.	Reduction reaction of dialdehydes (3) to diols (4) using $NaBH_4$.	10
2.3.1.	Copies of the NMR spectra of diols (4).	11
2.4.	Synthesis of 4a from fructose using four reaction steps.	15
2.5.	Meerwein–Ponndorf–Verley reduction reaction.	17
3.	Synthesis of polyurethanes (5) using diols (4) and commercial diisocyanates.	18
4.	Synthesis of polyurethanes (5) using 0.5 h of reaction.	19
5.	Copies of the RMN spectral of the polyurethanes (5).	22
6.	Copies of the infrared spectra obtained for the polyurethanes (5)	46
7.	Copies of the TGA thermograms and DTG curves obtained for the polyurethanes (5).	48
8.	Copies of the Debye plots obtained for each synthesized PU using 18 h of reaction.	52
9.	Copies of the Debye plots and molecular weights obtained for each synthesized PU using 0.5 h of reaction.	54
10	Synthesis of the polymer 5m via Curtius rearrangement.	56
11.	Degradation of polyurethane 5a using p-toluenesulfonic acid.	60

1. General Information

All commercially available reagents were used as purchased. Thin-layer chromatography (TLC) analyses were performed using silica gel plates, with UV absorption (254 nm) detection for visualization. All nuclear magnetic resonance (NMR) analyses were recorded using $CDCl_3$ or DMSO-d_6 as solvents and tetramethylsilane (TMS) as the internal standard. ^{1}H NMR spectra were recorded at 500 MHz or 400 MHz and ^{13}C{^{1}H} NMR spectra at 126 MHz or 100 MHz using 500 (Agilent Technologies-500/54 Premium Shielded) or 400 (Agilent Technologies-400/54 Premium Shielded) instruments. Chemical shifts are reported in ppm downfield from TMS concerning the internal solvent. Infrared spectra were obtained using FT-IR (Bruker, model ALPHA) at 4.0 cm^{-1} resolution and are reported in wavenumbers. The samples were dispersed neatly on a ZnSe crystal (ATR mode). TGA curves were obtained using a SDT-Q600 Simultaneous TGA/DTA (TA Instruments, USA), and a sample mass of *c.a.* 7.0 mg (weighed in thermobalance with precision of 0.1 µg). The heating rate was 10 °C min^{-1} under a nitrogen dynamic atmosphere (50 mL min^{-1}). Dynamic mechanical thermal analysis (DMTA) was performed in a Perkin Elmer Pyris DMA 8000 (Frequency = 1Hz). The temperature range was -100°C to 200°C and the heating rate was 5 °C min^{-1}. Test mode= Dual Cantilever, Material Pocket.

Molecular weight (M_w) was determined via static light scattering (SLS) using a Litesizer™ 500 DLS equipment provided with a Laser diode light source (output power = 40 mW, and λ_0 = 658 nm). Measurements were performed in a 10 mm optical path quartz cuvette, with four polished sides. The cuvette was washed in an ultrasonic bath using detergent (Extran MA 02), deionized water, and isopropyl alcohol, each step for 10 minutes, followed by drying in an oven for 2 hours. Data were collected using the side scattering angle detector (θ = 90°), with each data point was obtained by averaging over three measurements consisting of 12 runs each. The relationship between scattering intensity and M_w is made through the Debye-Zimm equation: $(K{\cdot}c/\Delta R) = (1/M_w) + 2{\cdot}A_2{\cdot}c$. Where K is an optical constant, c is the polymer concentration, ΔR is the Rayleigh ratio of a reference solvent, M_w is the polymer molecular weight, and A_2 is the second virial coefficient. The optical constant K is defined as $K = 4\pi^2{\cdot}(dn/dc)^2{\cdot}[(n_0^2)/(N_A{\cdot}\lambda_0^4)]$. Where dn/dc is the refractive index increment, n_0 is the refractive index of the solvent, N_A is Avogadro´s constant, and λ_0 is the wavelength of the incident beam. Toluene (ΔR = 1.1457x10^{-7} cm^{-1}) was used as the reference solvent, while DMSO (n_0 = 1.4768) was used as the solvent for all polymers samples. Debye plots were constructed by plotting $Kc/\Delta R$ against polymer concentration. We used three concentrations (15 mg mL^{-1}, 10 mg mL^{-1}, and 5 mg mL^{-1}). The molecular weight was obtained from the intercept of the fitted line, while the slope corresponded to A_2. Before measurements, all samples were filtered through a 0.45 µm syringe filter to remove impurities. Aliquots of 1 mL of each solution were used during the measurements. All the experiments were conducted at 25°C.

2. Synthesis of new biomass-derived diols from 5-(chloromethyl)furfural.

2.1 Synthesis of 5-(chloromethyl)furfural (CMF) from fructose (two-step).

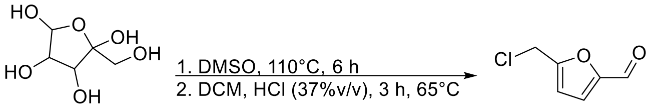

A 50 mL round bottom flask equipped with a distillation column was charged with 5 g of fructose (27.7 mmol) and 25 mL of dimethylsulfoxide (DMSO). The formed solution was heated at 110°C for 6 h. Then, 50 mL of distilled water was added and the obtained mixture was extracted with ethyl acetate (6 x 50 mL). The organic phases were mixed and washed with a saturated solution of sodium chloride, dried over anhydrous sodium sulfate, and evaporated using reduced pressure providing the 5-hydroxymethylfurfural (HMF) as the intermediate, which was employed in the next step without further purifications.

In the next step, a 250 mL round bottom flask equipped with a distillation column was charged with the HMF obtained in the last step, 100 mL of dichloromethane (DCM), and 50 mL of commercial HCl (37% v/v). The obtained mixture was vigorously stirred and heated at 65°C for 3 h. Upon completion, the organic phase was separated from the aqueous phase, and this last one was extracted with DCM (3 x 50 mL). Finally, the organic layers were combined, and dried over

anhydrous Na_2SO_4. The solvent was removed on a rotary evaporator providing the crude material, which was purified using a chromatography column loaded with silica gel pre-eluted with the mobile phase solvent (DCM). Chromatography afforded the desired CMF as a yellow oil in 58% yield (two steps). It is important to mention that CMF can be obtained directly from fructose using commercial HCl (37% v/v) and DCM or dichloroethane as the solvents.

5-(Chloromehtyl)furfural (CMF) = ^{1}H NMR (400 MHz, $CDCl_3$) δ 9.64 (s, 1H), 7.22 (d, *J* = 3.5 Hz, 1H), 6.60 (d, *J* = 3.5 Hz, 1H), 4.63 (s, 2H). ^{13}C NMR (101 MHz, $CDCl_3$) δ 177.79, 156.10, 152.90, 121.88, 112.01, 36.57.

Copies of the NMR spectra of CMF

^{1}H NMR (400 MHz, $CDCl_3$) 5-(chloromethyl)furfural

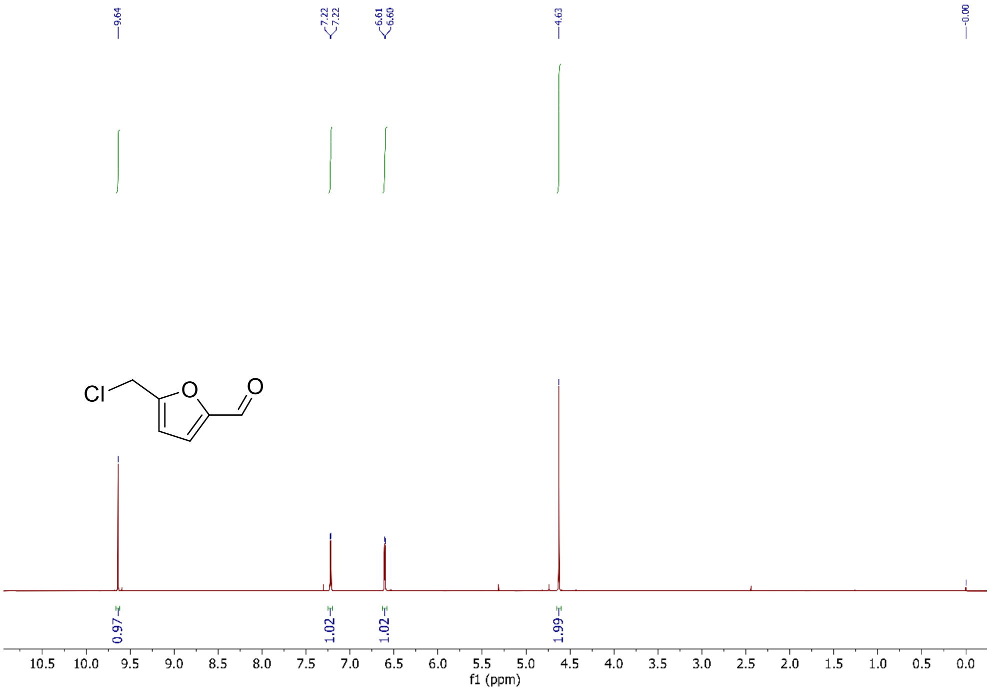

^{13}C NMR (101 MHz, $CDCl_3$) 5-(chloromethyl)furfural

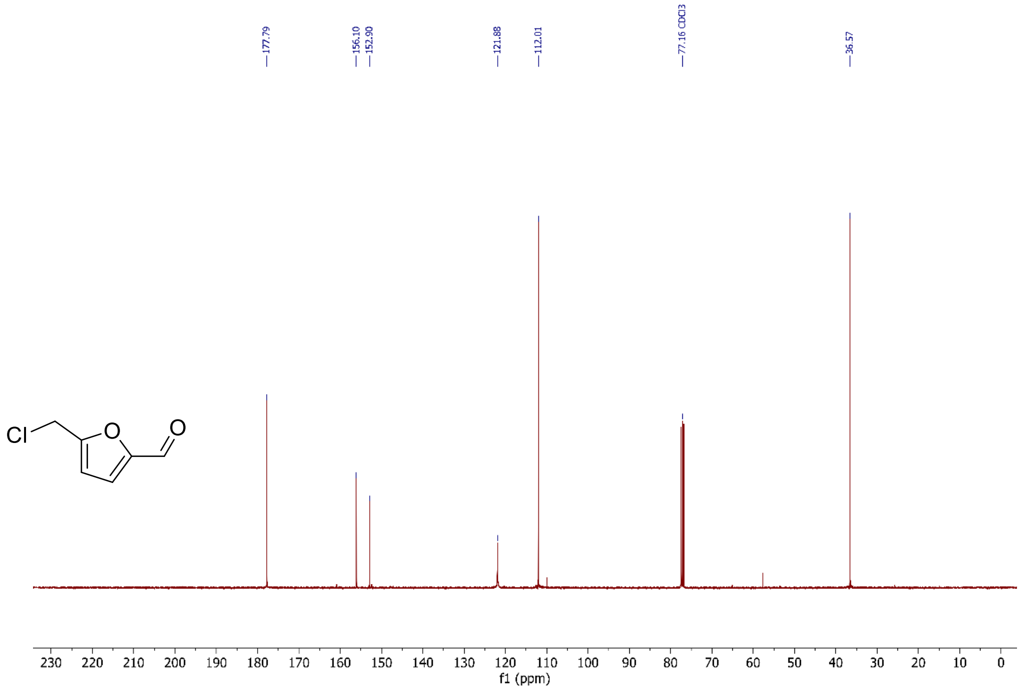

2.2 Synthesis of dialdehydes (3) from CMF and dithiols

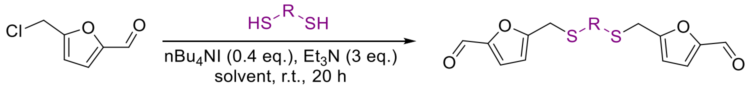

A 5 mL round bottom flask was charged with 3 mmol of CMF, 0.4 mmol of tetrabutylammonium iodide (nBu_4NI, 0.4 eq.), and 2.5 mL of 2-methyltetrahydrofuran (2-MeTHF). Subsequently, 1.0 mmol of the corresponding dithiol and 3 mmol of triethylamine (Et_3N, 3 eq.) were added. The formed mixture reacted at room temperature for 20 h. Upon completion, the solvent was evaporated under reduced pressure, and the obtained crude material was purified using a chromatography column loaded with silica gel pre-eluted with the mobile phase solvents (EtOAc/Hex 3:7). Chromatography afforded the desired products as oils or solids.

5,5'-((propane-1,3-diylbis(sulfanediyl))bis(methylene))bis(furan-2-carbaldehyde)(3a) = This product was synthesized using 3 mmol of CMF and 1 mmol of 1,3-propanedithiol as the starting materials. The purification by flash column chromatography on silica gel afforded 311 mg (98%) of the desired product as a yellow oil. **^{1}H NMR (500 MHz, $CDCl_3$) δ** 9.57 (s, 2H), 7.20 (d, J = 3.5 Hz, 2H), 6.44 (d, J = 3.5 Hz, 2H), 3.75 (s, 4H), 2.65 (t, J = 7.1 Hz, 4H), 1.85 (p, J = 7.1 Hz, 2H). **^{13}C NMR (126 MHz, $CDCl_3$) δ** 177.4, 159.4, 152.5, 110.5, 31.0, 28.5. **IR: v (neat, ATR)/cm^{-1}** 3115, 2917, 2824, 1671, 1579, 1512, 1361, 1279, 1240, 1194, 1136,1020, 965, 804, 770. **R_f** = 0.25 (EtOAc: Hex 3:7**). HMRS (ESI-TOF) m/z: [M + Na]$^+$** calculated for $C_{15}H_{16}O_4S_2$ 347.0382, found 347.0388

5,5'-((ethane-1,2-diylbis(sulfanediyl))bis(methylene))bis(furan-2-carbaldehyde) (**3b**) = This product was synthesized using 3 mmol of CMF and 1 mmol of 1,2-ethanedithiol as the starting materials. The purification by flash column chromatography on silica gel afforded 270 mg (87%) of the desired product as a white solid. **^{1}H NMR (500 MHz, $CDCl_3$)** δ 9.57 (s, 2H), 7.19 (d, *J* = 3.5 Hz, 2H), 6.45 (d, *J* = 3.6 Hz, 2H), 3.80 (s, 4H), 2.77 (s, 4H). **^{13}C NMR (126 MHz, $CDCl_3$)** δ 177.3, 159.1, 152.5, 110.6, 32.0, 28.6. **IR: v (neat, ATR)/cm^{-}**1 3114, 2915, 2824, 1665, 1579, 1511, 1393, 1361, 1278, 1243, 1193, 1020, 966, 804, 770,693. **R_f =** 0.28 (EtOAc: Hex 3:7**). m.p. =** 94-95 °C. **HMRS (ESI-TOF) m/z: [M + Na]$^+$** calculated for $C_{14}H_{14}O_4S_2$ 333.0232, found 333.0219.

5,5'-(((thiobis(4,1-phenylene))bis(sulfanediyl))bis(methylene))bis(furan-2-carbaldehyde) (3c) This product was synthesized using 3 mmol of CMF and 1 mmol of 4,4´-thiobisbenzenethiol as the starting materials. The purification by flash column chromatography on silica gel afforded 256 mg (55%) of the desired product as a yellow solid. **^{1}H NMR (500 MHz, $CDCl_3$)** δ 9.55 (s, 2H), 7.30 – 7.18 (m, 8H), 7.13 (s, 2H), 6.34 (dd, *J* = 3.6, 0.8 Hz, 2H), 4.12 (s, 4H). **^{13}C NMR (126 MHz, $CDCl_3$)** δ 177.4, 158.2, 152.4, 134.8, 133.8, 131.6, 110.9, 31.7. **IR: v (neat, ATR)/cm^{-1}** 3116, 2921, 2825, 1672, 1576, 1513, 1474, 1392, 1361, 1279, 1240,1193, 1098, 1074, 1020, 966, 868, 805, 771, 754, 696. **R_f** = 0.30 (EtOAc: Hex 3:7**). m.p. =** 72-74°C **HMRS (ESI-TOF) m/z: [M + Na]$^+$** calculated for $C_{24}H_{18}O_4S_3$ 489.0259, found 489.0286.

5,5'-(5,8-dioxa-2,11-dithiadodecane-1,12-diyl)bis(furan-2-carbaldehyde) (3d) = This product was synthesized using 3 mmol of CMF and 1 mmol of 2,2'-(Ethylenedioxy)diethanethiol as the starting materials. The purification by flash column chromatography on silica gel afforded 342 mg (89%) of the desired product as a yellow oil. **^{1}H NMR (500 MHz, $CDCl_3$)** δ 9.56 (s, 2H), 7.18 (d, *J* = 3.5 Hz, 2H), 6.45 (dd, *J* = 3.6, 0.7 Hz, 2H), 3.84 (s, 4H), 3.66 (t, *J* = 6.4 Hz, 4H), 3.60 (s, 4H), 2.73 (t, *J* = 6.4 Hz, 4H). **^{13}C NMR (126 MHz, $CDCl_3$) δ** 177.34, 159.48, 152.43, 110.59, 71.13, 70.44, 31.61, 28.84. **IR: v (neat, ATR)/cm^{-1}** 2915, 2863, 1665, 1580, 1511, 1474, 1395, 1353, 1281, 1247, 1194, 1101, 1019, 965, 865, 804, 767, 754, 698. **R_f** = 0.07 (EtOAc: Hex 3:7). **HMRS (ESI-TOF) m/z: [M + Na]$^+$** calculated for $C_{18}H_{22}O_6S_2$ 421.0756, found 421.0742.

5-(((3-mercaptopropyl)thio)methyl)furan-2-carbaldehyde (3a') = This product was obtained as a byproduct in the reaction between CMF and 1,3-propanedithiol. **^{1}H NMR (500 MHz, $CDCl_3$)** δ 9.58 (s, 1H), 7.20 (d, *J* = 3.5 Hz, 1H), 6.45 (d, *J* = 3.6 Hz, 1H), 3.77 (s, 2H), 2.69 (t, *J* = 7.1 Hz, 2H), 2.62 (dt, *J* = 8.1, 6.9 Hz, 2H), 1.88 (p, *J* = 7.0 Hz, 2H), 1.35 (t, *J* = 8.1 Hz, 1H). **^{13}C NMR (126 MHz, $CDCl_3$) δ IR: v (neat, ATR)/cm^{-1}** 2922, 2826, 1668, 1579, 1512, 1361, 1279, 1241, 1195, 1135, 1020, 966, 803, 770, 756. **R_f** = 0.32 (EtOAc: Hex 3:7**).**

2.2.1 Copies of the NMR spectra of dialdehydes

^{1}H NMR (500 MHz, $CDCl_3$) 5,5'-((propane-1,3-diylbis(sulfanediyl))bis(methylene))bis(furan-2-carbaldehyde)(3a)

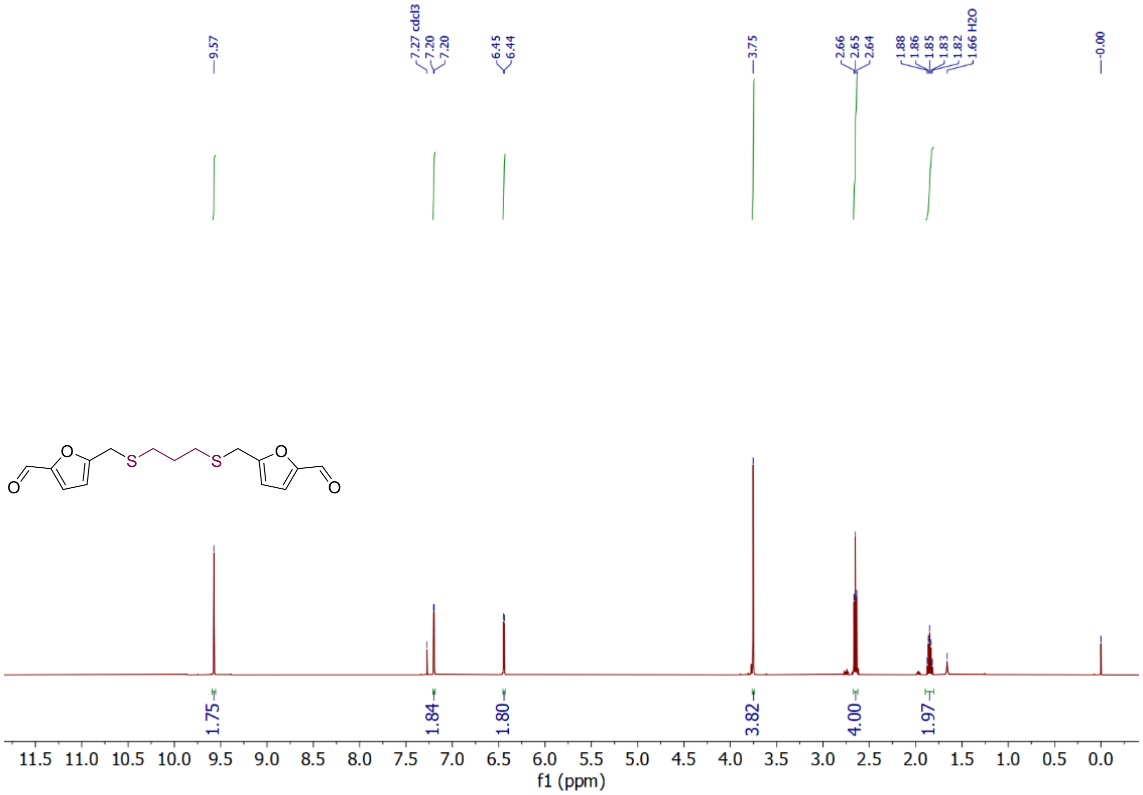

^{13}C NMR (126 MHz, $CDCl_3$) 5,5'-((propane-1,3-diylbis(sulfanediyl))bis(methylene))bis(furan-2-carbaldehyde)(3a)

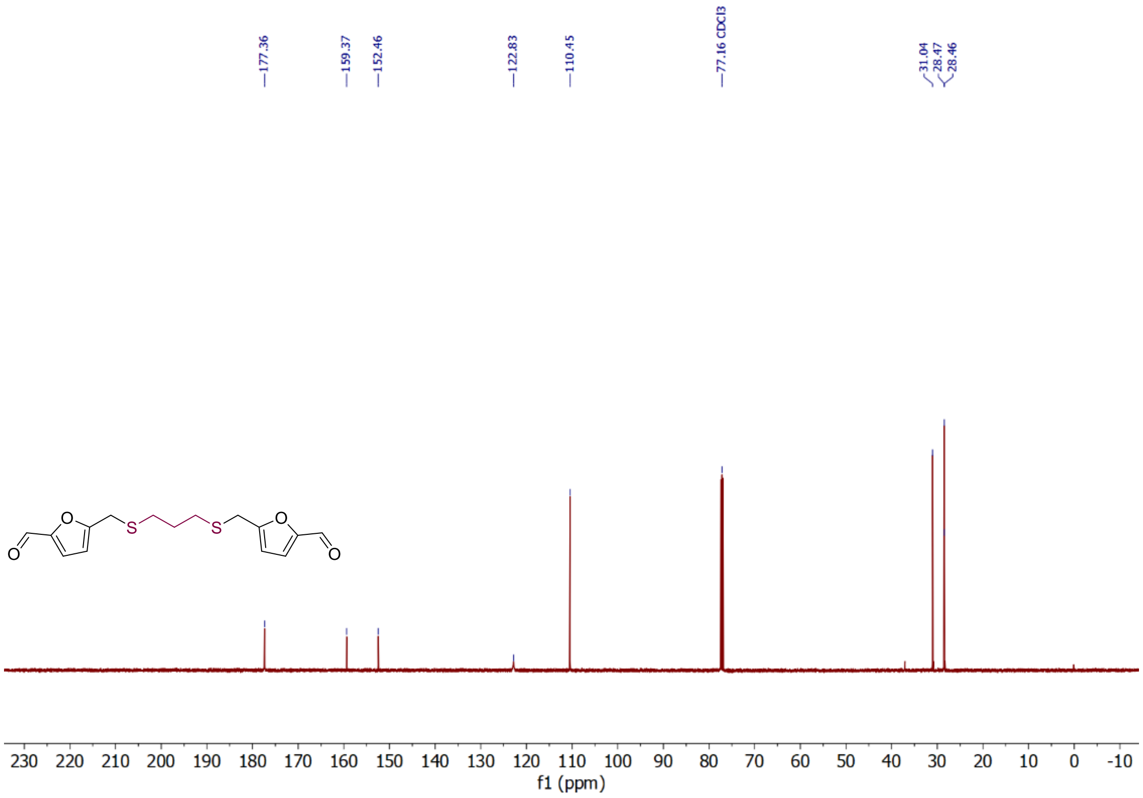

^{1}H NMR (500 MHz, $CDCl_3$) 5,5'-((ethane-1,2-diylbis(sulfanediyl))bis(methylene))bis(furan-2-carbaldehyde)

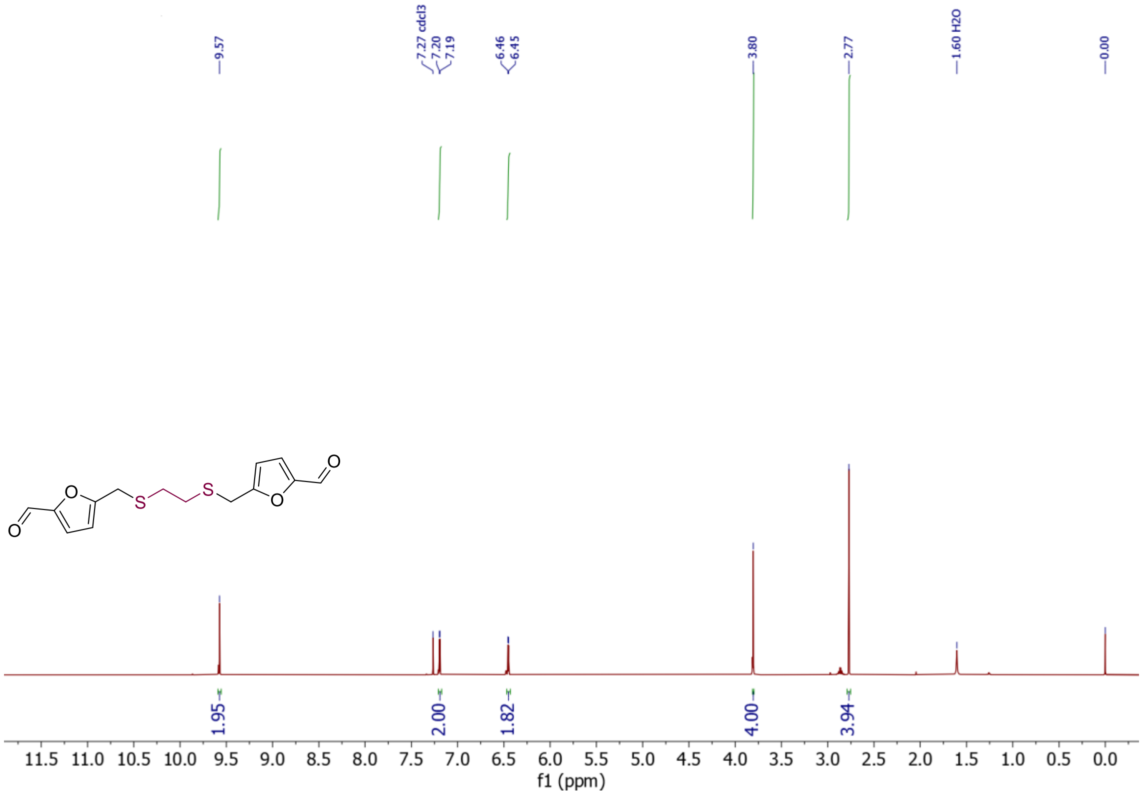

^{13}C NMR (126 MHz, $CDCl_3$) 5,5'-((ethane-1,2-diylbis(sulfanediyl))bis(methylene))bis(furan-2-carbaldehyde)

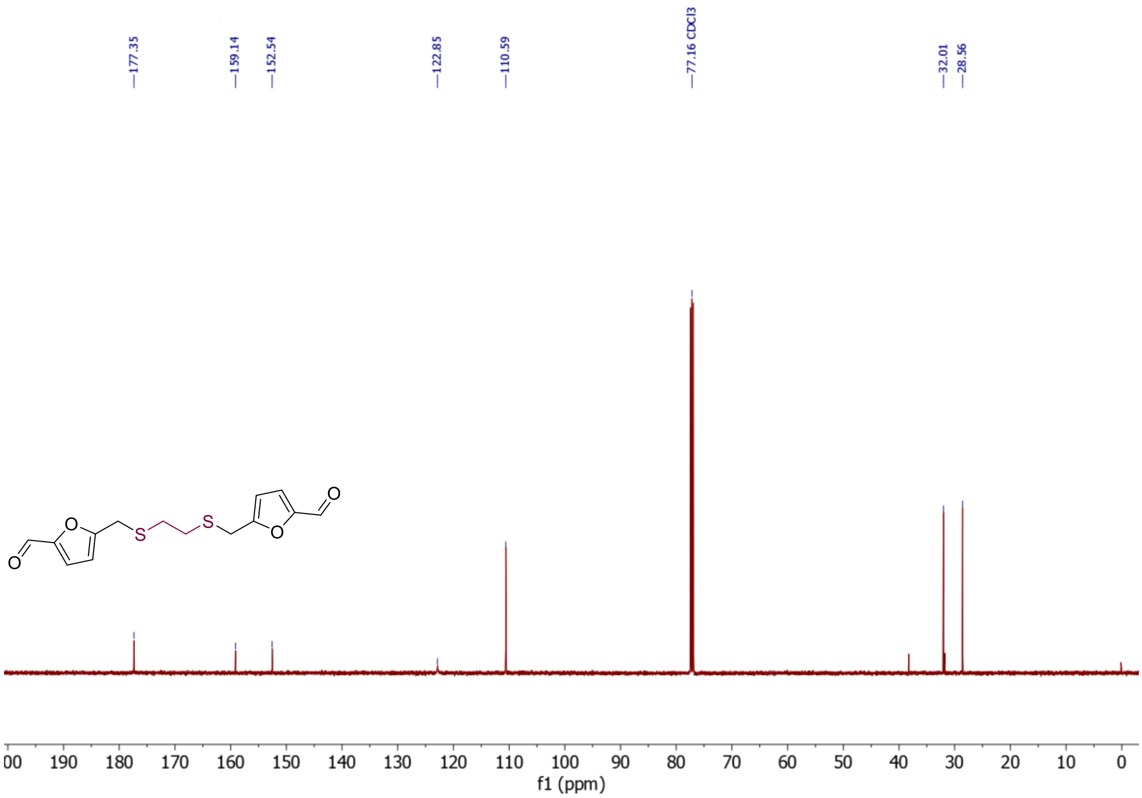

^1H NMR (500 MHz, $CDCl_3$)
5,5'-(((thiobis(4,1-phenylene))bis(sulfanediyl))bis(methylene))bis(furan-2-carbaldehyde)

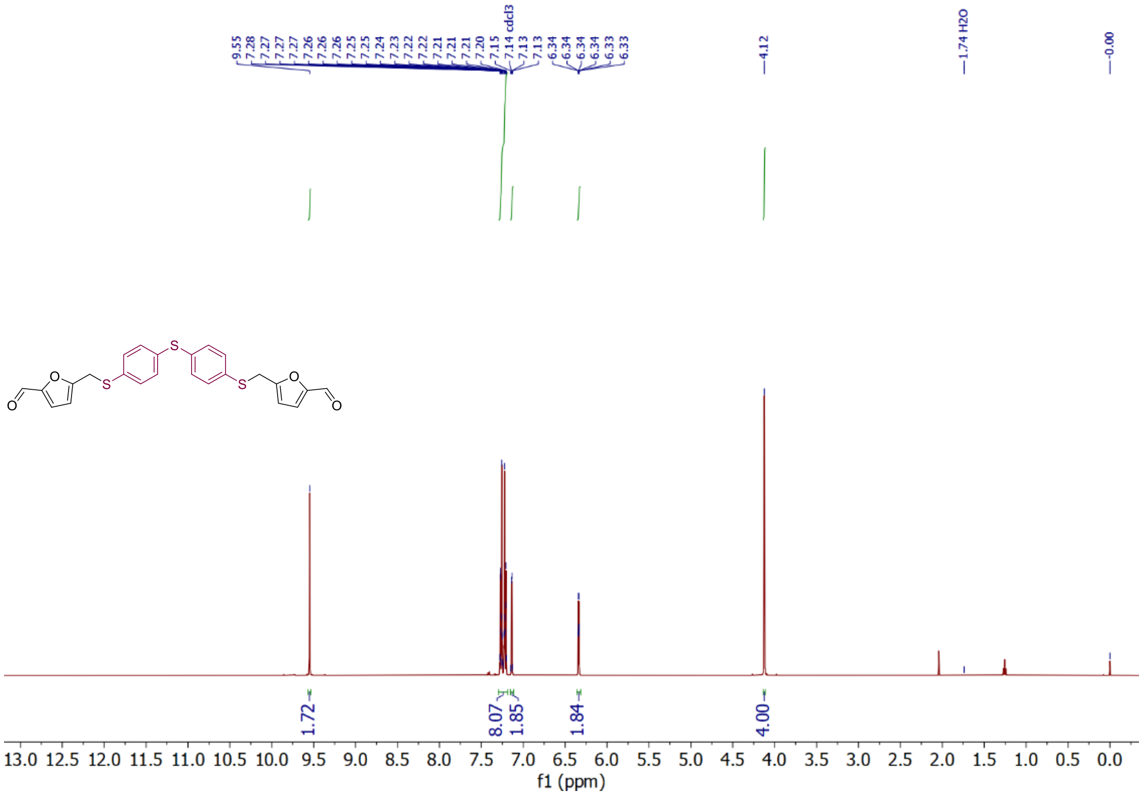

^{13}C NMR (126 MHz, $CDCl_3$)
5,5'-(((thiobis(4,1-phenylene))bis(sulfanediyl))bis(methylene))bis(furan-2-carbaldehyde)

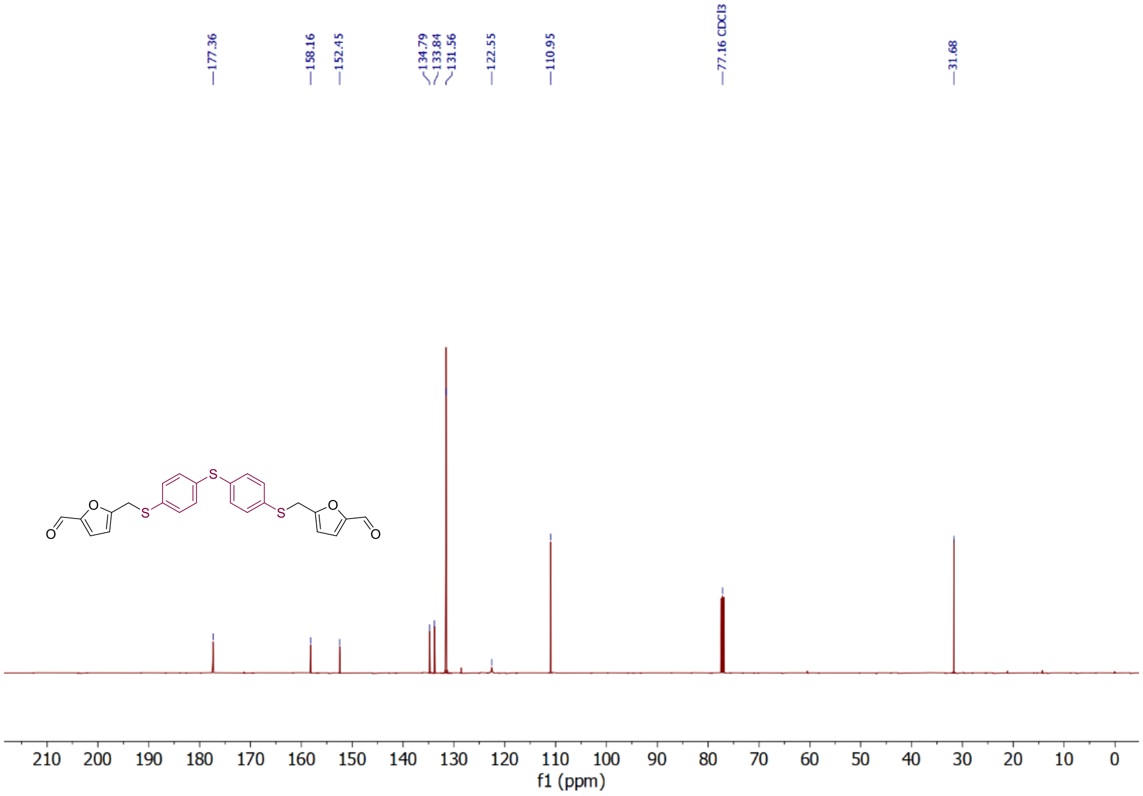

^{1}H NMR (500 MHz, $CDCl_3$) 5,5'-(5,8-dioxa-2,11-dithiadodecane-1,12-diyl)bis(furan-2-carbaldehyde) (3d)

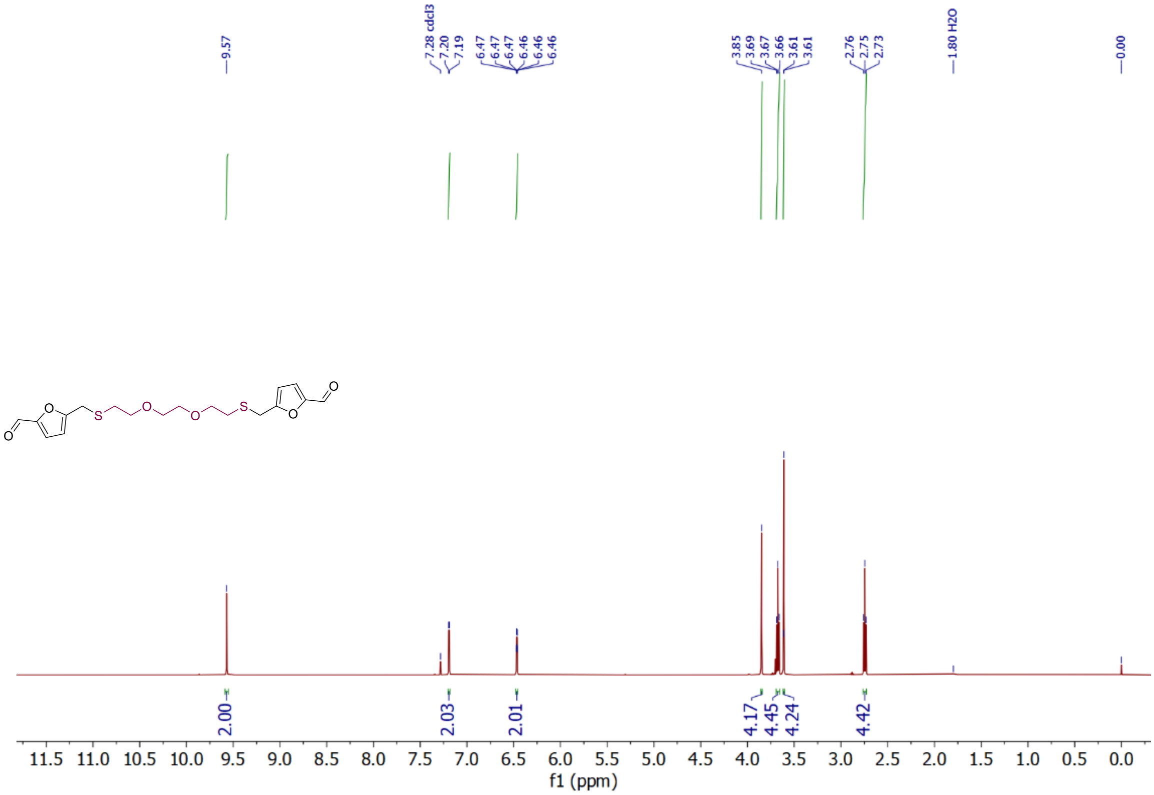

^{13}C NMR (126 MHz, $CDCl_3$) 5,5'-(5,8-dioxa-2,11-dithiadodecane-1,12-diyl)bis(furan-2-carbaldehyde) (3d)

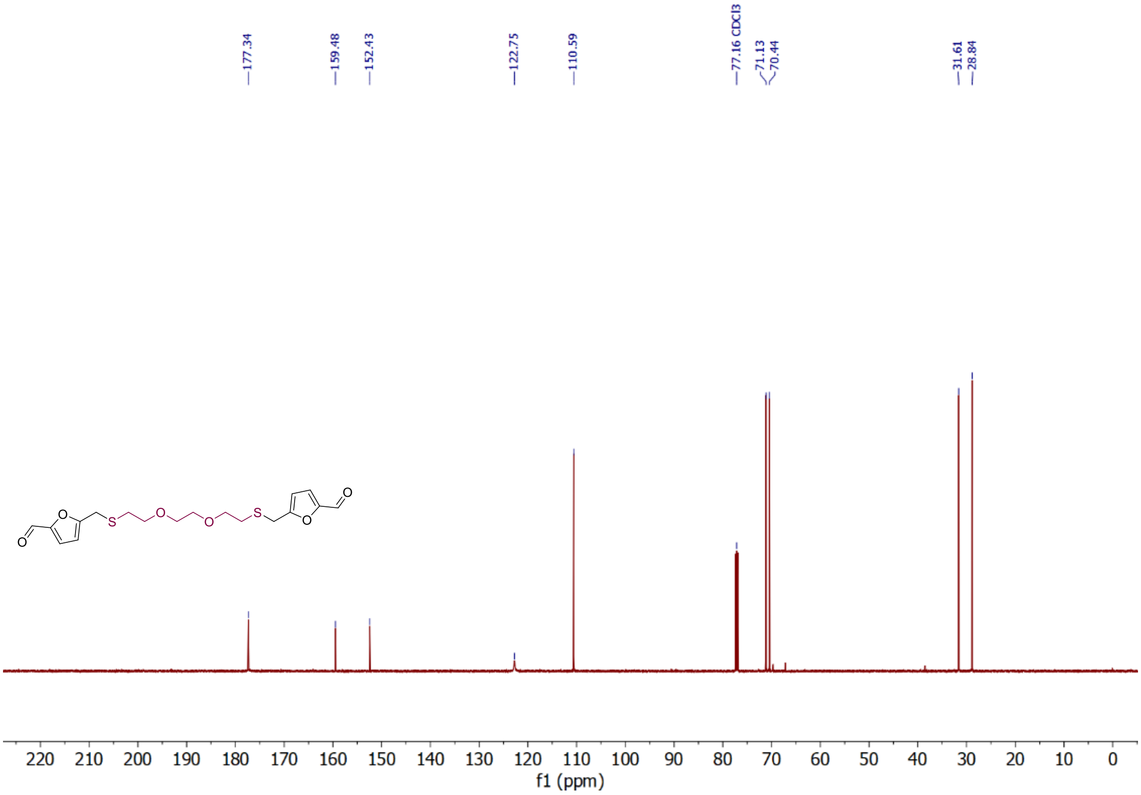

^{1}H NMR (500 MHz, $CDCl_3$) 5-(((3-mercaptopropyl)thio)methyl)furan-2-carbaldehyde (3a')

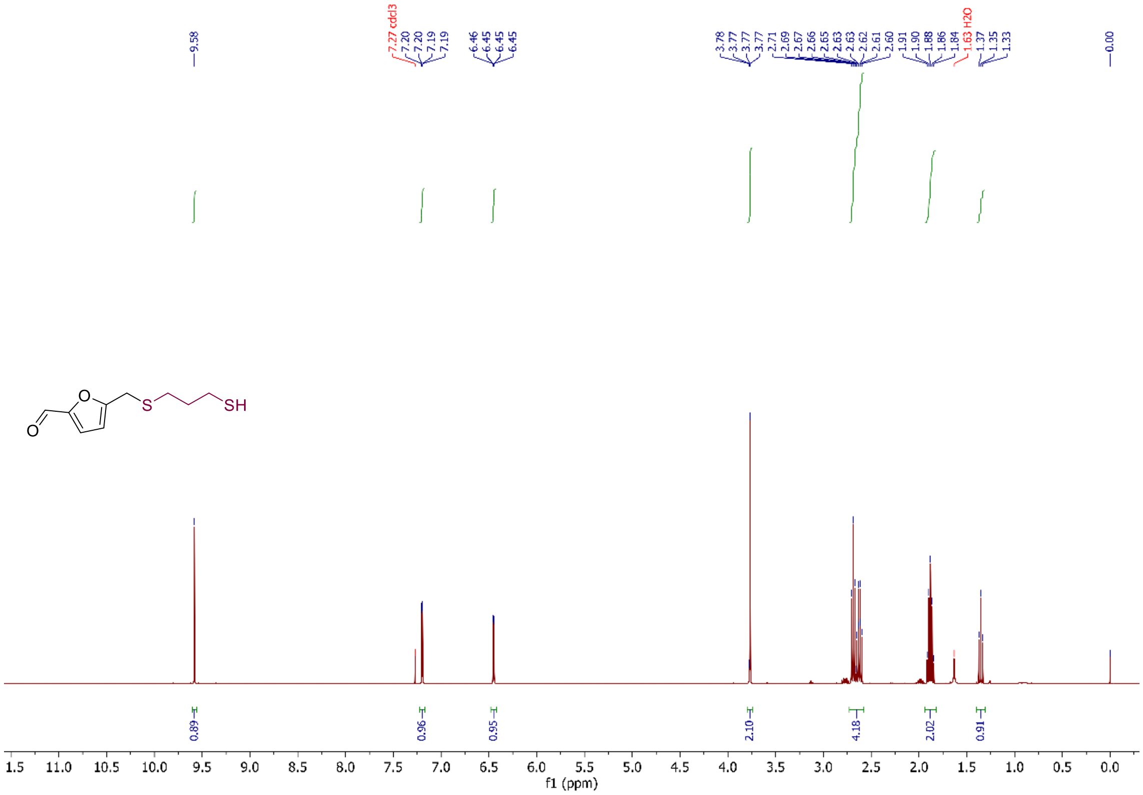

^{13}C NMR (126 MHz, $CDCl_3$) 5-(((3-mercaptopropyl)thio)methyl)furan-2-carbaldehyde (3a')

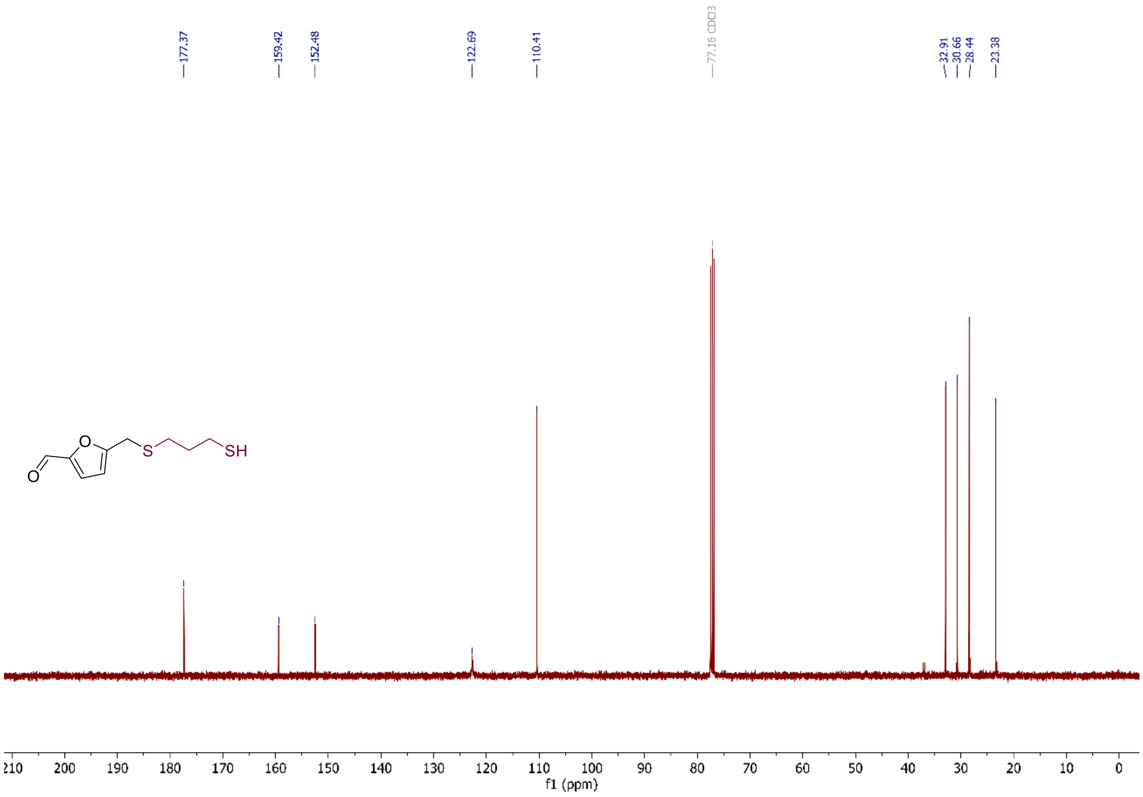

2.3 Reduction reaction of dialdehydes (3) to diols (4) using $NaBH_4$

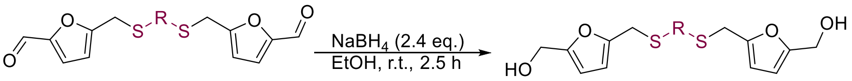

A 25 mL round bottom flask was charged with 1 mmol of the appropriate dialdehyde, 2.4 mmol of $NaBH_4$, and 15 mL of ethanol. The formed mixture reacted at room temperature for 2.5 h. Upon completion, the solvent was evaporated under reduced pressure, and the crude material was purified using a chromatography column loaded with silica gel pre-eluted with the mobile phase solvents (EtOAc/Hex 1:1).

(((propane-1,3-diylbis(sulfanediyl))bis(methylene))bis(furan-5,2-diyl))dimethanol (4a) = The purification by flash column chromatography on silica gel afforded 292 mg (89%) of the desired product as a white solid. **^{1}H NMR (500 MHz, DMSO-d_6)** δ 6.18 (s, 4H), 5.16 (t, *J* = 5.7 Hz, 2H), 4.33 (d, *J* = 5.8 Hz, 4H), 3.72 (s, 4H), 2.54 – 2.51 (m, 4H), 1.74 (p, *J* = 7.2 Hz, 2H).**^{13}C NMR (126 MHz, DMSO-d_6)** δ 154.9, 150.8, 108.1, 107.7, 55.7, 29.9, 28.3, 27.3. **IR: v (neat, ATR)/cm^{-1}** 3369, 2923, 2870, 1554, 1418, 1404, 1253, 1229, 1188, 1143, 1011, 757. **R_f** = 0.32 (EtOAc: Hex 1:1). **m.p. =** 81-82°C. **HMRS (ESI-TOF) m/z: [M + Na]$^+$** calculated for $C_{15}H_{20}O_4S_2$ 351.0695, found 351.0696.

(((ethane-1,2-diylbis(sulfanediyl))bis(methylene))bis(furan-5,2-diyl))dimethanol (4b) = The purification by flash column chromatography on silica gel afforded 296 mg (94%) of the desired product as a white solid. **^{1}H NMR (500 MHz, DMSO-d_6)** δ 6.18 (s, 4H), 5.17 (t, *J* = 5.8 Hz, 2H), 4.33 (d, *J* = 5.8 Hz, 4H), 3.77 (s, 4H), 2.65 (s, 4H). **^{13}C NMR (126 MHz, DMSO-d_6)** δ 154.9, 150.7, 108.22, 107.7, 55.7, 30.9, 27.3. **IR: v (neat, ATR)/cm^{-1}** 3357, 2923, 1557, 1405, 1248, 1227, 1196, 1130, 1002, 978, 957, 789, 754, 716. **R_f** = 0.22 (EtOAc: Hex 1:1). **m.p. =** 80-82°C. **HMRS (ESI-TOF) m/z: [M + Na]$^+$** calculated for $C_{14}H_{18}O_4S_2$ 337.0529, found 337.0536.

((((thiobis(4,1-phenylene))bis(sulfanediyl))bis(methylene))bis(furan-5,2-diyl))dimethanol (4c) = The purification by flash column chromatography on silica gel afforded 442 mg (94%) of the desired product as a white solid. **^{1}H NMR (500 MHz, DSMO-d_6)** δ 7.38 – 7.32 (m, 4H), 7.27 – 7.20 (m, 4H), 6.18 (d, *J* = 3.1 Hz, 2H), 6.16 (d, *J* = 3.1 Hz, 2H), 5.17 (s, 2H), 4.32 (s, 4H), 4.25 (s, 4H). **^{13}C NMR (126 MHz, DSMO-d_6)** δ 155.1, 149.5, 135.3, 132.2, 131.2, 129.48, 108.8, 107.7, 55.6, 29.4. **IR: v (neat, ATR)/cm^{-1}** 3358, 2924, 2866, 1573, 1555, 1475, 1390, 1252, 1225, 1100, 1010, 800, 748. **R_f** = 0.29 (EtOAc: Hex 1:1). **m.p. =** 80-83 °C. **HMRS (ESI-TOF) m/z: [M + Na]$^+$** calculated for $C_{24}H_{22}O_4S_3$ 493.0572, found 493.0577.

((5,8-dioxa-2,11-dithiadodecane-1,12-diyl)bis(furan-5,2-diyl))dimethanol (4d) = The purification by flash column chromatography on silica gel afforded 370 mg (92%) of the desired product as a white solid. **^{1}H NMR (500 MHz, DMSO-d_6)** δ 6.20 – 6.15 (m, 4H), 5.16 (t, *J* = 5.7 Hz, 2H), 4.33 (d, *J* = 5.8 Hz, 4H), 3.77 (s, 4H), 3.56 – 3.48 (m, 8H), 2.62 (t, *J* = 6.7 Hz, 4H). **^{13}C NMR (126 MHz, DMSO-d_6)** δ 154.9, 150.8, 108.2, 107.6, 70.0, 69.5, 55.7, 30.4, 27.8. **IR: v (neat, ATR)/cm^{-1}**, 3386, 2920, 2865, 1634, 1555, 1097, 1009, 979, 794, 752. **R_f** = 0.32 (EtOAc: Hex 1:1**). m.p. =** 53-54°C**. HMRS (ESI-TOF) m/z: [M + Na]$^+$** calculated for $C_{18}H_{26}O_6S_2$ 425.1069, found 425.1060.

2.3.1 Copies of the NMR spectra of diols (4)

^{1}H NMR (500 MHz, DMSO-d_6) (((propane-1,3-diylbis(sulfanediyl))bis(methylene))bis(furan-5,2-diyl))dimethanol (4a)

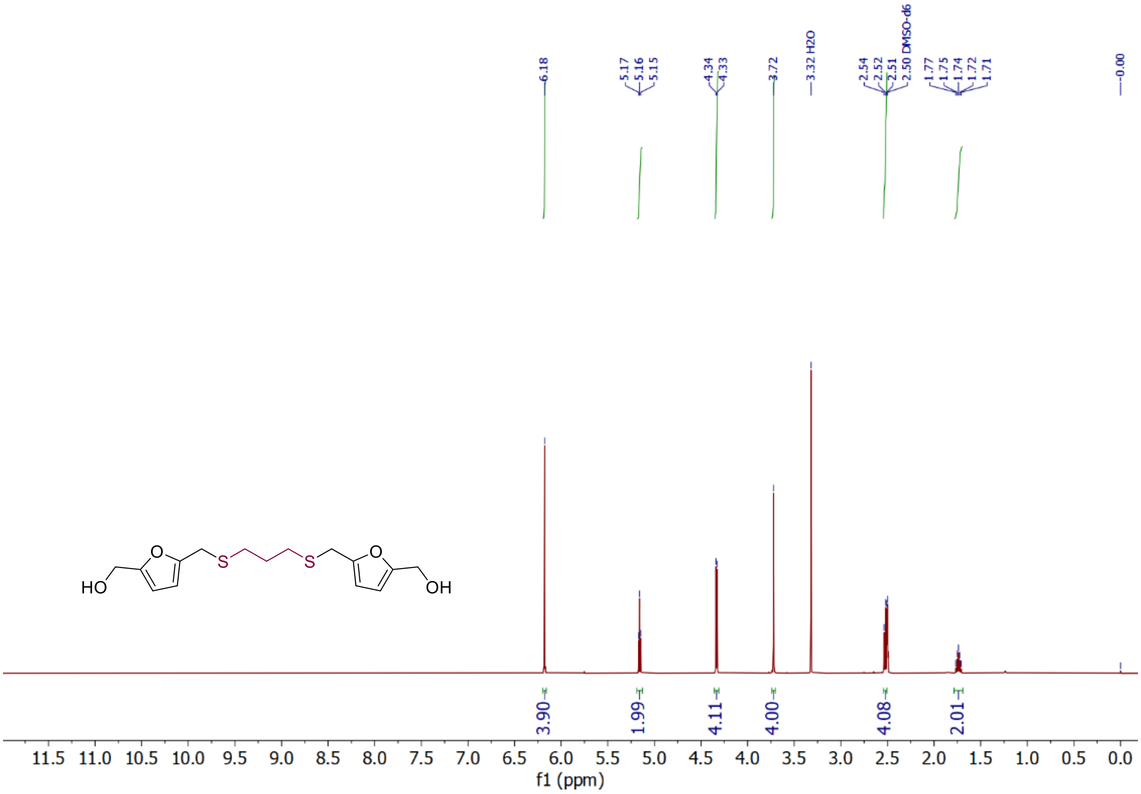

^{13}C NMR (126 MHz, DMSO-d_6) (((propane-1,3-diylbis(sulfanediyl))bis(methylene))bis(furan-5,2-diyl))dimethanol (4a)

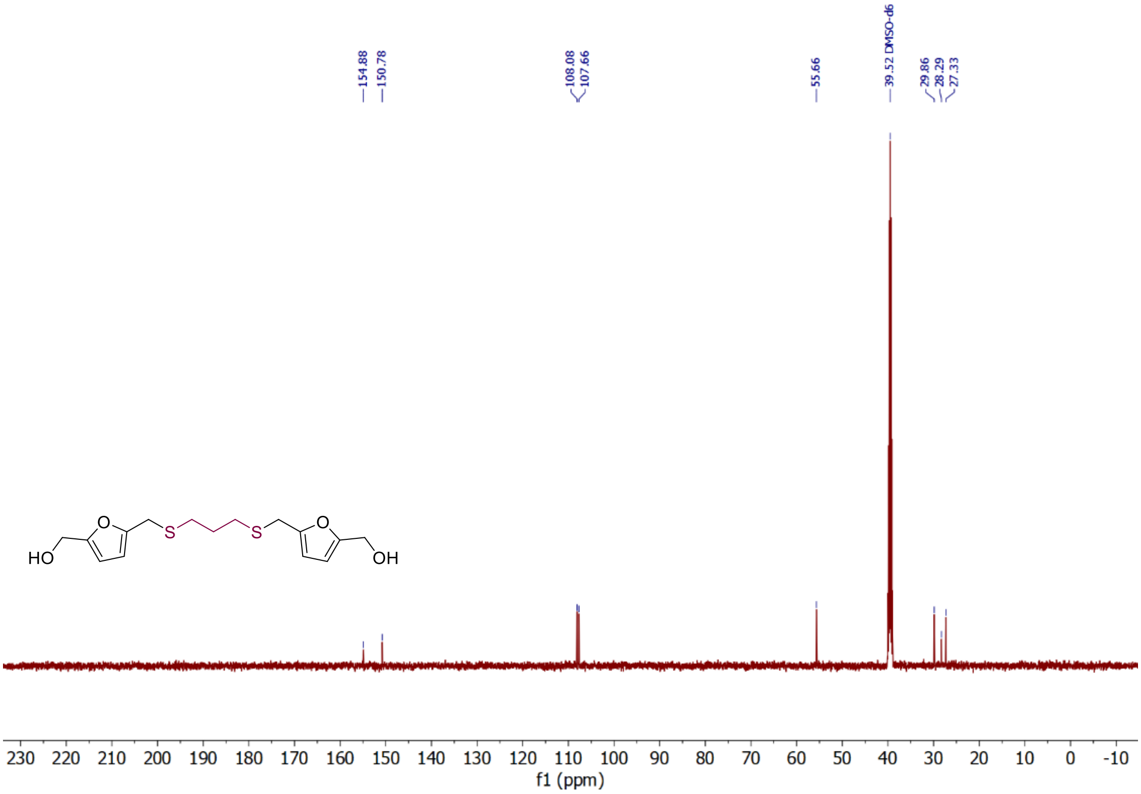

^{1}H NMR (500 MHz, DMSO-d_6) (((ethane-1,2-diylbis(sulfanediyl))bis(methylene))bis(furan-5,2-diyl))dimethanol (4b)

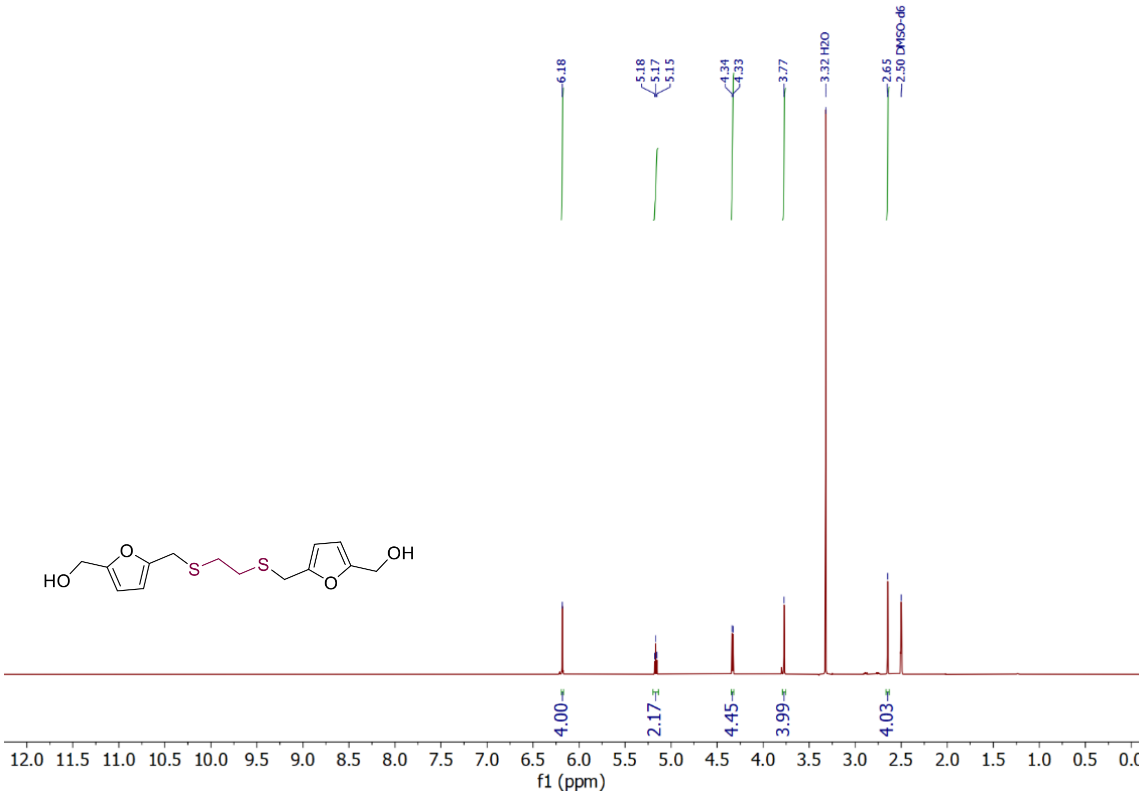

^{13}C NMR (126 MHz, DMSO-d_6) (((ethane-1,2-diylbis(sulfanediyl))bis(methylene))bis(furan-5,2-diyl))dimethanol (4b)

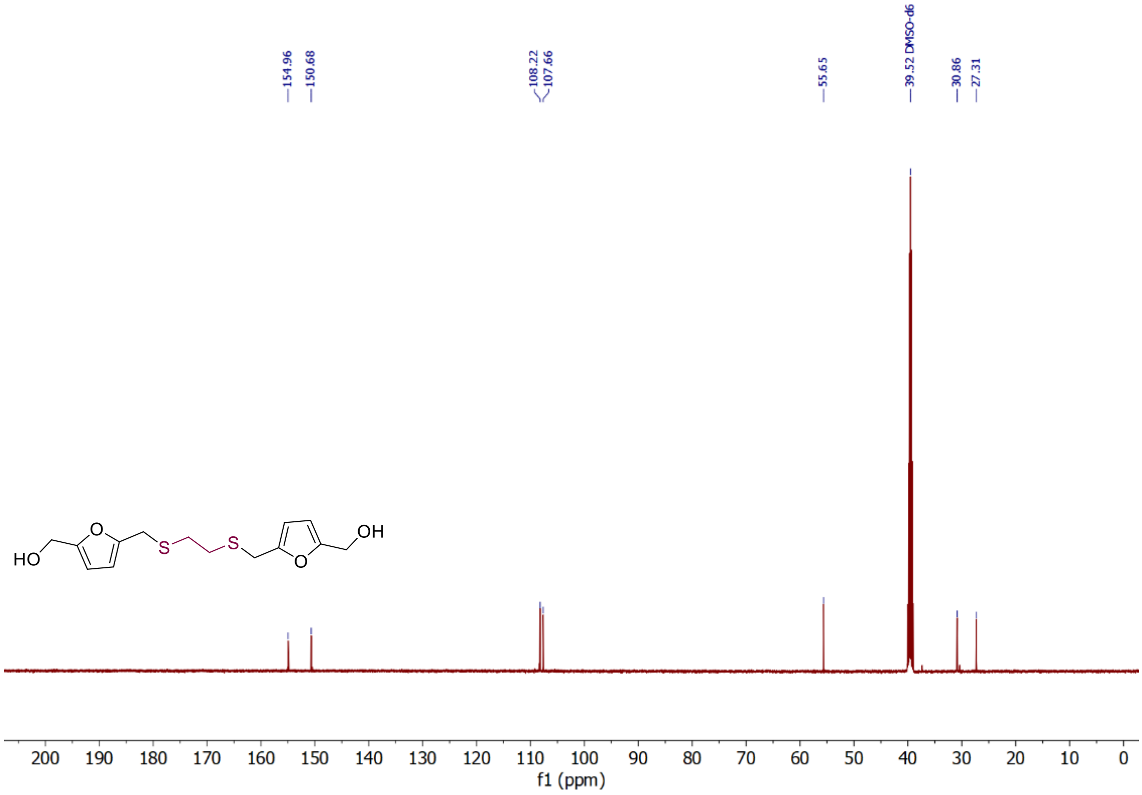

^{1}H NMR (500 MHz, DMSO-d_6)

((((thiobis(4,1-phenylene))bis(sulfanediyl))bis(methylene))bis(furan-5,2-diyl))dimethanol (4c)

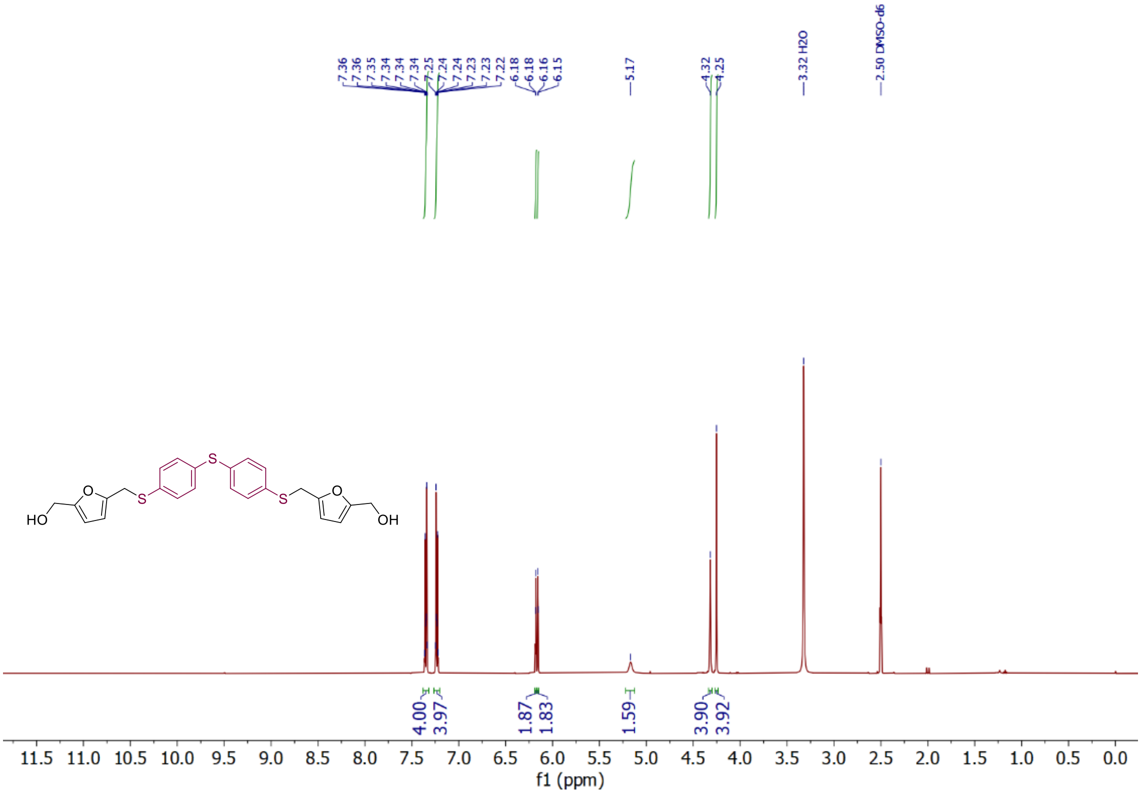

^{13}C NMR (126 MHz, DMSO-d_6)

((((thiobis(4,1-phenylene))bis(sulfanediyl))bis(methylene))bis(furan-5,2-diyl))dimethanol (4c)

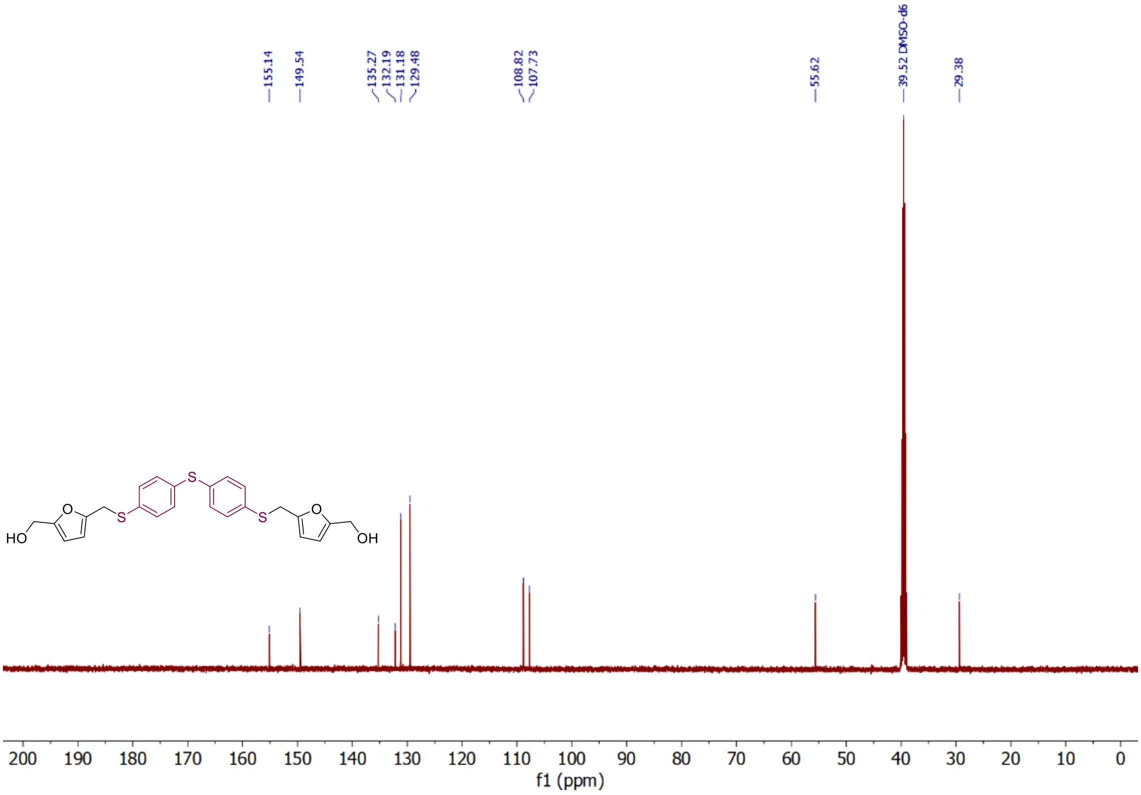

^{1}H NMR (500 MHz, DMSO-d_6) ((5,8-dioxa-2,11-dithiadodecane-1,12-diyl)bis(furan-5,2-diyl))dimethanol (4d)

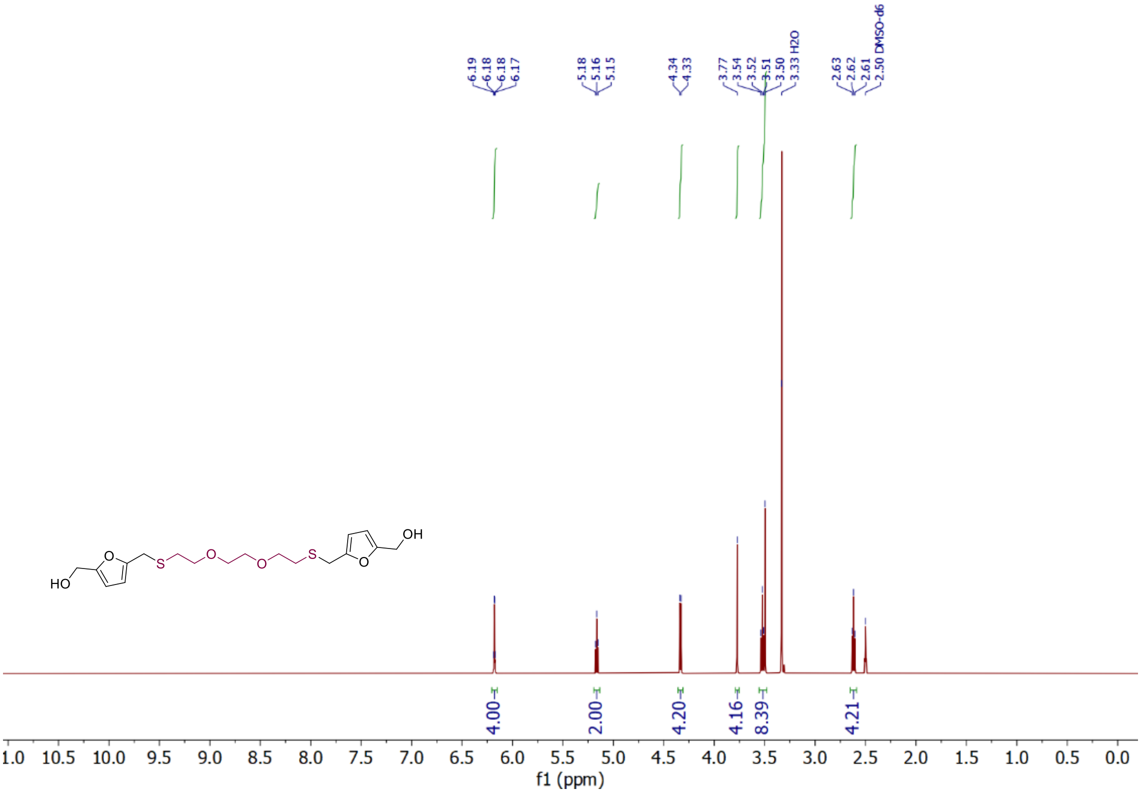

^{13}C NMR (126 MHz, DMSO-d_6) ((5,8-dioxa-2,11-dithiadodecane-1,12-diyl)bis(furan-5,2-diyl))dimethanol (4d)

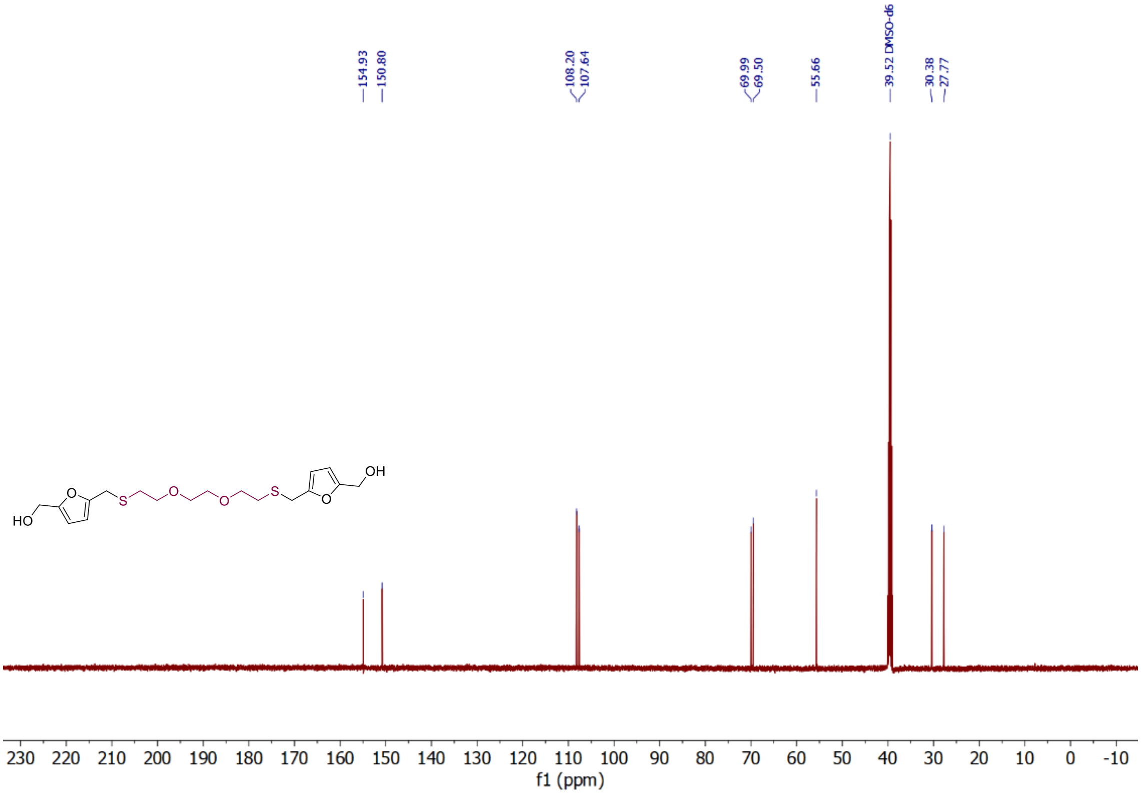

2.4 Synthesis of 4a from fructose using four reaction steps.

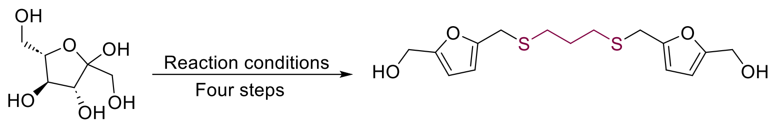

Fructose (55. 5 mmol, 10 g)

A 50 mL round bottom flask equipped with a distillation column was charged with 10 g of fructose (55.5 mmol) and 50 mL of DMSO. The formed solution reacted at 110°C for 6 h. Then, 100 mL of distilled water was added and the obtained mixture was extracted with ethyl acetate (6 x 100 mL). The organic phases were mixed and washed with a saturated solution of sodium chloride, dried over anhydrous sodium sulfate, and evaporated using reduced pressure providing the 5-hydroxymethylfurfural (HMF) as the intermediate, which was employed in the next step without further purifications.

In the next step, a 500 mL round bottom flask equipped with a distillation column was charged with the HMF obtained in the last step, 200 mL of dichloromethane (DCM), and 100 mL of commercial HCl (37% v/v). The obtained mixture was vigorously stirred and heated at 65°C for 3 h. Upon completion, the organic phase was separated from the aqueous phase, and this last one was extracted with DCM (3 x 100 mL). Finally, the organic layers were combined, and dried over anhydrous Na_2SO_4. The solvent was removed on a rotary evaporator providing the crude material, which was employed in the next step.

In the next step, a 50 mL round bottom flask was charged with the previous crude material, 1,3-propanedithiol (10.74 mmol), Et_3N (32.22 mmol), nBu_4NI (4.30 mmol), and 27 mL of 2-Me-THF. The formed solution reacted at room temperature for 20 h. Upon completion, a white solid was obtained, which was filtered and discarded. The solvent was evaporated under reduced pressure providing the dialdehyde (**3**). Then, $NaBH_4$ (25.78 mmol) and 50 mL of ethanol were added, and the mixture reacted at room temperature for 2.5 h. Finally, the solvent was evaporated and 50 mL of water was added. The aqueous phase was extracted with ethyl acetate (6 x 50 mL). Then, the organic layers were mixed, washed with a saturated solution of NaCl, and dried over Na_2SO_4. The solvent was evaporated under reduced pressure providing the desired diol **4a**, which was recrystallized using ethyl acetate/hexane (three times). The recrystallization provided a pure material with a 47% yield (1.657 g, 5.05 mmol).

Copies of the NMR spectral obtained for the analytical and the recrystallized samples = $CDCl_3$ was employed as the solvent. ^{1}H NMR (500 MHz, cdcl$_3$) δ 6.21 (d, *J* = 3.1 Hz, 2H), 6.12 (d, *J* = 3.0 Hz, 2H), 4.56 (d, *J* = 3.4 Hz, 4H), 3.68 (s, 4H), 2.59 (t, *J* = 7.2 Hz, 4H), 2.21 (s, 2H), 1.77 (p, *J* = 7.2 Hz, 2H).

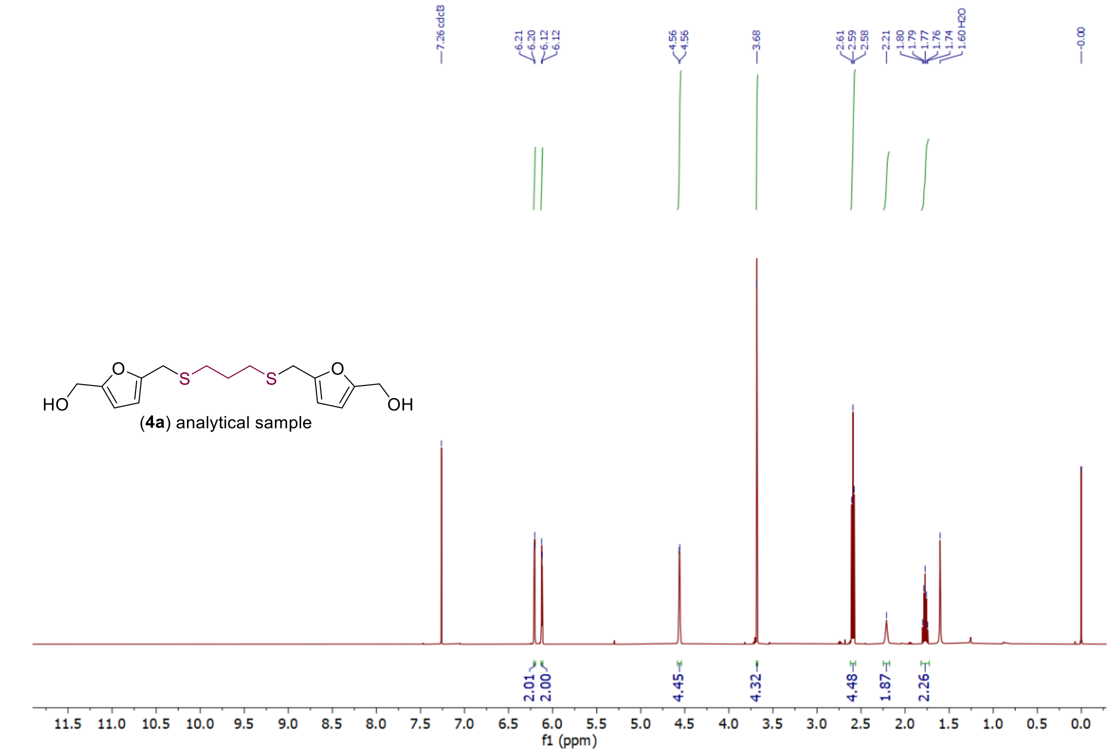

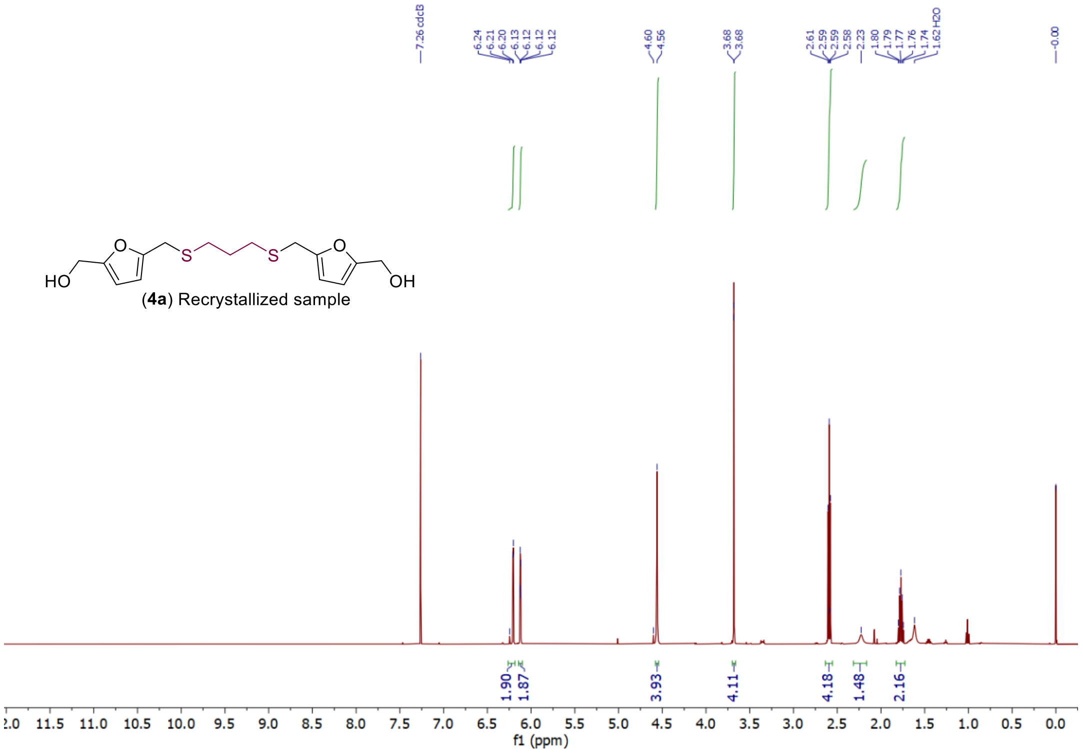

2.4 Meerwein–Ponndorf–Verley reduction reaction

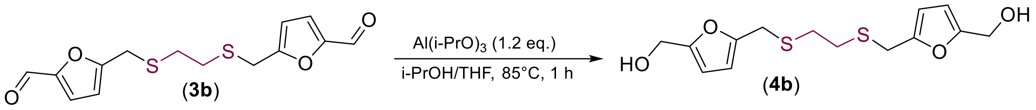

A 10 mL round-bottom flask fitted with a distillation column was charged with 0.5 mmol of the dialdehyde and 6 mL of a solvent mixture (7:2 isopropanol/THF). The mixture was stirred until dissolved. Then, 0.6 mmol of aluminum isopropoxide (1.2 eq.) was added, and the reaction was heated to 85°C for 1 hour. After completion, 5 mL of 1M HCl was added, and the solution was stirred for 15 minutes. Water was then added, and the aqueous phase was extracted twice with 5 mL of EtOAc. The combined organic phases were dried over Na_2SO_4, and the solvent was evaporated under reduced pressure. The crude product was purified by silica gel column chromatography using a hexane/EtOAc (1:1) eluent, yielding the corresponding diol in 97% yield (154 mg). The same procedure was repeated with 3.22 mmol (1.0 g) of the starting material. After extraction, the obtained solid was recrystallized using EtOAc/Hex, providing the desired diol **4b** in excellent yield (87%, 0.881 g).

(((ethane-1,2-diylbis(sulfanediyl))bis(methylene))bis(furan-5,2-diyl))dimethanol (4b) = ^{1}H NMR (500 MHz, $CDCl_3$) δ 6.21 (dt, *J* = 3.2, 0.5 Hz, 2H), 6.14 – 6.12 (m, 2H), 4.56 (s, 4H), 3.72 (s, 4H), 2.69 (s, 4H), 2.06 (s, 1H).

Copy of the ^{1}H NMR (500 MHz, $CDCl_3$) of the obtained diol after recrystallization

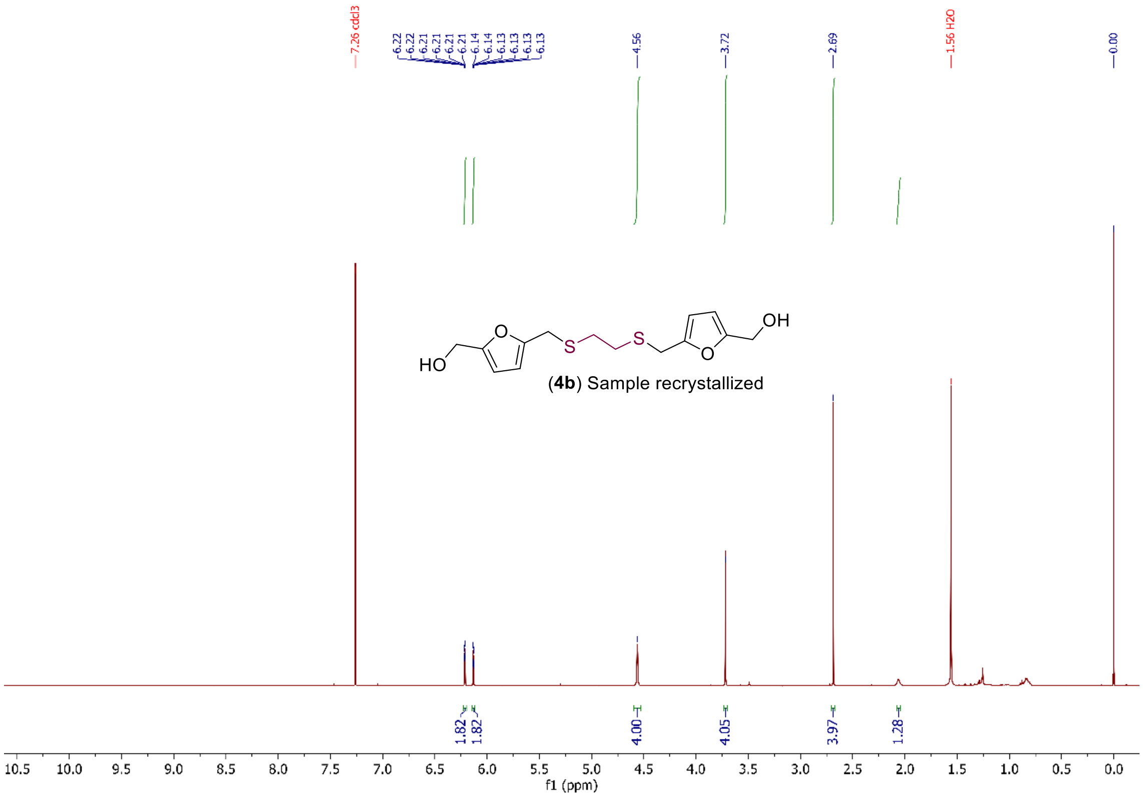

3. Synthesis of polyurethanes using diols (4) and commercial diisocyanates

A 10 mL round bottom flask with a distillation column was charged with 0.5 mmol of the corresponding diol (**4**), 20 mol% of DBU, and 5 mL of dry THF. Then, 0.5 mmol of the corresponding commercial diisocyanate was added. The formed mixture reacted at 40°C for 18 h. Upon completion, methanol was added and a white precipitate was obtained, which was filtered and washed with methanol providing the desired polyurethane.

Polyurethane (**5a**) = This product was obtained using diol **4a** and hexamethylene diisocyanate (HDI). The product was obtained as a white solid after precipitation with methanol (218 mg, 88%). **^{1}H NMR (500 MHz, DMSO-d_6) δ** 7.20 (t, *J* = 5.7 Hz, 2H), 6.37 (d, *J* = 3.1 Hz, 2H), 6.23 (d, *J* = 2.9 Hz, 2H), 4.90 (s, 4H), 3.73 (s, 4H), 2.95 (q, *J* = 6.6 Hz, 4H), 2.53 (d, *J* = 7.2 Hz, 4H), 1.74 (p, *J* = 7.1 Hz, 2H), 1.35 (t, *J* = 7.0 Hz, 4H), 1.22 (d, *J* = 6.6 Hz, 4H). **^{13}C NMR (126 MHz, DMSO-d_6) δ** 155.7, 152.22, 149.7, 111.12, 108.4, 57.4, 29.9, 29.3, 28.2, 27.2, 25.9. **IR: v (neat, ATR)/cm^{-1}** 3307, 2938, 2858, 1681, 1559, 1338, 1263, 1221, 1136, 1003, 987, 966, 796, 776.

Polyurethane (**5b**) = This product was obtained using diol **4a** and 1,4-diphenylene diisocyanate (PPDI). The product was obtained as a white solid after precipitation with methanol (205 mg, 84%). **^{1}H NMR (500 MHz, DMSO-d_6) δ** 9.61 (s, 2H), 7.31 (s, 4H), 6.43 (d, *J* = 3.2 Hz, 2H), 6.22 (d, *J* = 3.1 Hz, 2H), 5.01 (s, 4H), 3.71 (s, 4H), 2.50 (t, *J* = 7.2 Hz, 4H), 1.72 (p, *J* = 7.1 Hz, 2H). **^{13}C NMR (126 MHz, DMSO-d_6) δ** 153.0, 152.5, 149.1, 133.8, 118.8, 0111.7, 108.4, 57.7, 29.9, 28.2, 27.2. **IR: v (neat, ATR)/cm^{-1}** 3288, 1697, 1554, 1523, 1410, 1305, 1238, 1069, 1018, 943, 794, 765.

Polyurethane (5c) = This product was obtained using diol **4a** and 4,4′-methylenebis(phenyl isocyanate) (MDI). The product was obtained as a white solid after precipitation with methanol (218 mg, 72%). **^{1}H NMR (500 MHz, DMSO-d_6)** δ 9.67 (s, 2H), 7.35 (d, *J* = 8.1 Hz, 4H), 7.08 (d, *J* = 8.4 Hz, 4H), 6.45 (d, *J* = 3.1 Hz, 2H), 6.24 (d, *J* = 3.2 Hz, 2H), 5.04 (s, 4H), 3.73 (s, 6H), 2.53 (t, *J* = 7.1 Hz, 4H), 1.74 (p, *J* = 7.2 Hz, 2H). **^{13}C NMR (126 MHz, DMSO-d_6)** δ 153.0, 152.5, 149.1, 136.9, 135.6, 128.7, 118.3, 111.7, 108.4, 57.7, 29.9, 28.2, 27.2. **IR: v (neat, ATR)/cm^{-1}** 3298, 3124, 2914, 1700, 1596, 1530, 1433, 1411, 1309, 1220, 1048, 1016. 969, 942, 794, 766, 719.

Polyurethane (**5d**) = This product was obtained using diol **4b** and hexamethylene diisocyanate (HDI). The product was obtained as a white solid after precipitation with methanol (194 mg, 80%). **^{1}H NMR (500 MHz, DMSO-d_6)** δ 7.20 (t, *J* = 5.7 Hz, 2H), 6.37 (d, *J* = 3.2 Hz, 2H), 6.23 (d, *J* = 3.1 Hz, 2H), 4.89 (s, 4H), 3.77 (s, 4H), 2.94 (q, *J* = 6.6 Hz, 4H), 2.65 (s, 4H), 1.34 (q, *J* = 7.6 Hz, 4H), 1.21 (d, *J* = 6.0 Hz, 4H). **^{13}C NMR (126 MHz, DMSO-d_6)** δ 155.7, 152.1, 149.78, 111.2, 108.5, 57.4, 40.2, 30.9, 29.3, 27.2, 25.9. **IR: v (neat, ATR)/cm^{-1}** 3305, 2929, 2856, 1684, 1536, 1263, 1222, 1138, 1053, 987, 795, 777.

Polyurethane (**5e**) = This product was obtained using diol **4b** and 1,4-diphenylene diisocyanate (PPDI). The product was obtained as a white solid after precipitation with methanol (202 mg, 85%). **^{1}H NMR (500 MHz, DMSO-d_6)** δ 9.64 (s, 2H), 7.33 (s, 4H), 6.46 (d, *J* = 3.3 Hz, 2H), 6.26 (d, *J* = 3.2 Hz, 2H), 5.04 (s, 4H), 3.80 (s, 4H), 2.68 (s, 4H). **^{13}C NMR (126 MHz, DMSO-d_6)** δ 153.0, 152.4, 149.2, 133.8, 118.8, 111.7, 108.6, 57.7, 30.9, 27.1. **IR: v (neat, ATR)/cm^{-1}** 3297, 1695, 1554, 1522, 1410, 1302, 1232, 1067, 1016, 794, 768.

Polyurethane (5f) = This product was obtained using diol **4b** and 4,4′-methylenebis(phenyl isocyanate) (MDI). The product was obtained as a white solid after precipitation with methanol (204 mg, 72%). **^{1}H NMR (500 MHz, DMSO-d_6)** δ 9.67 (s, 2H), 7.34 (d, *J* = 8.1 Hz, 4H), 7.08 (d, *J* = 8.5 Hz, 4H), 6.45 (d, *J* = 3.2 Hz, 2H), 6.25 (d, *J* = 3.1 Hz, 2H), 5.03 (s, 4H), 3.78 (d, *J* = 6.4 Hz, 6H), 2.67 (s, 4H). **^{13}C NMR (126 MHz, DMSO-d_6)** δ 153.0, 152.4, 149.2, 136.9, 135.6, 128.9, 118.3, 111.7, 108.6, 57.7, 30.9, 27.1. **IR: v (neat, ATR)/cm^{-1}** 3307, 1700, 1597, 1533, 1519,1412, 1311, 1232, 1139,1050, 1015, 794, 765.

Polyurethane (**5g**) = This product was obtained using diol **4c** and hexamethylene diisocyanate (HDI). The product was obtained as a white solid after precipitation with methanol (220 mg, 69%). **^{1}H NMR (500 MHz, DMSO-d$_6$)** δ 7.33 (d, *J* = 8.2 Hz, 4H), 7.20 (dd, *J* = 13.3, 7.0 Hz, 6H), 6.34 (d, *J* = 3.1 Hz, 2H), 6.21 (d, *J* = 3.1 Hz, 2H), 4.87 (s, 4H), 4.24 (s, 4H), 2.93 (d, *J* = 6.7 Hz, 4H), 1.34 (s, 4H), 1.19 (s, 4H). ^{13}C NMR (126 MHz, **DMSO-d$_6$**) δ 155.7, 151.0, 149.9, 135.1, 132.3, 131.2, 129.6, 111.20, 109.1, 57.3, 40.2, 29.3, 25.9. **IR: v (neat, ATR)/cm^{-1}** 3306, 2934, 2857, 1681, 1533, 1473, 1252, 1220, 1132, 1097, 1049, 1005, 777, 743.

Polyurethane (**5h**) = This product was obtained using diol **4c** and 1,4-diphenylene diisocyanate (PPDI). The product was obtained as a white solid after precipitation with methanol (240 mg, 76%). **^{1}H NMR (500 MHz, DMSO-d$_6$)** δ 9.64 (s, 2H), 7.38 – 7.31 (m, 8H), 7.24 – 7.20 (m, 4H), 6.43 (d, *J* = 3.2 Hz, 2H), 6.25 (d, *J* = 3.0 Hz, 2H), 5.02 (s, 4H), 4.27 (s, 4H). **^{13}C NMR (126 MHz, DMSO-d$_6$)** δ 153.02, 151.30, 149.39, 135.06, 133.85, 132.34, 131.23, 129.56, 118.80, 111.74, 109.21, 57.67, 29.26. **IR: v (neat, ATR)/cm^{-1}** 3315, 2928, 1699, 1605, 1520, 1473, 1408, 1305, 1204, 1097, 1049, 1010, 797, 764.

Polyurethane (5i) = This product was obtained using diol **4c** and 4,4′-methylenebis(phenyl isocyanate) (MDI). The product was obtained as a white solid after precipitation with methanol (235 mg, 65%). **^{1}H NMR (500 MHz, DMSO-d$_6$)** δ 9.67 (s, 2H), 7.34 (dq, *J* = 9.6, 2.5 Hz, 8H), 7.24 – 7.19 (m, 4H), 7.10 – 7.05 (m, 4H), 6.43 (d, *J* = 3.2 Hz, 2H), 6.24 (d, *J* = 3.2 Hz, 2H), 5.02 (s, 4H), 4.26 (s, 4H), 3.77 (s, 2H). **^{13}C NMR (126 MHz, DMSO-d$_6$)** δ 152.95, 151.30, 149.33, 136.85, 135.57, 135.03, 132.32, 131.18, 129.55, 128.86, 118.33, 111.73, 109.17, 57.65, 29.25. **IR: v (neat, ATR)/cm^{-1}** 3304, 1698, 1598, 1518, 1473, 1388, 1309, 1223, 1203, 1097, 1048, 1013, 795, 770.

Polyurethane (**5j**) = This product was obtained using diol **4d** and hexamethylene diisocyanate (HDI). The product was obtained as a white solid after precipitation with methanol (200 mg, 70%). **^{1}H NMR (500 MHz, DMSO-d$_6$)** δ 7.21 (s, 2H), 6.37 (d, *J* = 3.1 Hz, 2H), 6.23 (d, *J* = 3.0 Hz, 2H), 4.89 (s, 4H), 3.77 (d, *J* = 6.1 Hz, 4H), 3.55 – 3.46 (m, 8H), 2.94 (t, *J* = 6.6 Hz, 4H), 2.62 (t, *J* = 6.6 Hz, 4H), 1.35 (s, 4H), 1.21 (s, 4H). **^{13}C NMR (126 MHz, DMSO-d$_6$)** δ 155.7, 152.2, 149.7, 111.1, 108.5, 70.0, 69.5, 57.3, 40.2, 30.5, 29.3, 27.6, 25.9. **IR: v (neat, ATR)/cm^{-1}** 3307, 2936, 2858, 1680, 1536, 1263, 1222, 1114, 1005, 986, 966, 940, 794, 776.

Polyurethane (**5k**) = This product was obtained using diol **4d** and 1,4-diphenylene diisocyanate (PPDI). The product was obtained as a white solid after precipitation with methanol (210 mg, 75%). **^{1}H NMR (500 MHz, DMSO-d$_6$)** δ 9.64 (s, 2H), 7.34 (s, 4H), 6.45 (d, *J* = 3.2 Hz, 2H), 6.26 (d, *J* = 3.2 Hz, 2H), 5.04 (s, 4H), 3.79 (s, 4H), 3.53 – 3.45 (m, 8H), 2.62 (t, *J* = 6.7 Hz, 4H). **^{13}C NMR (126 MHz, DMSO-d$_6$)** δ 153.1, 152.6, 149.2, 133.9, 118.8, 111.7, 108.6, 70.0, 69.5, 57.7, 30.5, 27.6. **IR: v (neat, ATR)/cm^{-1}** 3290, 2919, 2860, 1693, 1552, 1519, 1408, 1304, 1209, 1108, 1067, 1046, 1014, 792, 763.

Polyurethane (5l) = This product was obtained using diol **4d** and 4,4′-methylenebis(phenyl isocyanate) (MDI). The product was obtained as a white solid after precipitation with methanol (215 mg, 66%). **^{1}H NMR (500 MHz, DMSO-d$_6$)** δ 9.68 (s, 2H), 7.34 (dd, *J* = 8.4, 3.1 Hz, 4H), 7.08 (qd, *J* = 7.4, 4.5 Hz, 5H), 6.49 – 6.42 (m, 2H), 6.25 (d, *J* = 3.2 Hz, 2H), 5.04 (s, 4H), 3.79 (s, 4H), 3.55 – 3.41 (m, 8H), 2.61 (t, *J* = 6.6 Hz, 4H). **^{13}C NMR (126 MHz, DMSO-d$_6$)** δ 153.0, 152.6, 149.1, 136.9, 135.6, 128.9, 128.9, 128.8, 118.3, 111.7, 108.6, 70.0, 69.5, 57.7, 30.5, 27.6. **IR: v (neat, ATR)/cm^{-1}** 3300, 2924, 2856, 1700, 1598, 1528, 1512, 1411, 1310, 1215, 1109, 1041, 1015, 794, 765.

4. Synthesis of polyurethanes using 0.5 h of reaction

A 10 mL round bottom flask with a distillation column was charged with 0.5 mmol of the corresponding diol (**4**), 20 mol% of DBU, and 5 mL of dry THF. Then, 0.5 mmol of the corresponding commercial diisocyanate was added. The formed mixture reacted at 40°C for 0.5

h. Upon completion, methanol was added and a white precipitate was obtained, which was filtered and washed with methanol providing the desired polyurethane.

Polyurethane (**5a**) = The product was obtained as a white solid after precipitation with methanol (223 mg, 90%). **^{1}H NMR (500 MHz, DMSO-d$_6$)** δ 7.20 (t, *J* = 5.7 Hz, 2H), 6.37 (d, *J* = 3.1 Hz, 2H), 6.23 (d, *J* = 3.0 Hz, 2H), 4.90 (s, 4H), 3.73 (s, 4H), 2.95 (q, *J* = 6.6 Hz, 4H), 2.53 (t, *J* = 7.2 Hz, 4H), 1.74 (p, *J* = 7.3 Hz, 2H), 1.36 (t, *J* = 6.7 Hz, 4H), 1.21 (s, 4H). **^{13}C NMR (126 MHz, DMSO-d$_6$)** δ 155.7, 152.2, 149.7, 111.1, 108.4, 57.4, 29.9, 29.3, 28.2, 27.2, 25.9.

Polyurethane (**5b**) = The product was obtained as a white solid after precipitation with methanol (199 mg, 82%). **^{1}H NMR (500 MHz, DMSO-d$_6$)** δ 9.64 (s, 2H), 7.34 (s, 4H), 6.46 (d, *J* = 3.2 Hz, 2H), 6.25 (d, *J* = 3.1 Hz, 2H), 5.04 (s, 4H), 3.74 (s, 4H), 2.53 (t, *J* = 7.1 Hz, 4H), 1.75 (p, *J* = 7.1 Hz, 2H). **^{13}C NMR (126 MHz, DMSO-d$_6$)** δ 153.0, 152.5, 149.1, 133.8, 118.8, 111.7, 108.42, 57.7, 29.9, 28.2, 27.2.

Polyurethane (5c) = The product was obtained as a white solid after precipitation with methanol (200 mg, 69%). **^{1}H NMR (400 MHz, DMSO-d$_6$)** δ 9.67 (s, 2H), 7.34 (d, *J* = 8.1 Hz, 4H), 7.13 – 7.03 (m, 4H), 6.45 (d, *J* = 3.2 Hz, 2H), 6.24 (d, *J* = 3.2 Hz, 2H), 5.04 (s, 4H), 3.80 – 3.70 (m, 6H), 2.53 (d, *J* = 7.2 Hz, 4H), 1.74 (p, *J* = 7.3 Hz, 2H). **^{13}C NMR (101 MHz, DMSO-d$_6$)** δ 153.0, 152.5, 149.1, 136.9, 135.6, 128.9, 118.3, 111.7, 108.4, 57.7, 29.9, 28.2, 27.2.

Polyurethane (**5d**) = The product was obtained as a white solid after precipitation with methanol (198 mg, 82%). **^{1}H NMR (500 MHz, DMSO-d$_6$)** δ 7.20 (s, 2H), 6.38 (d, *J* = 3.1 Hz, 2H), 6.23 (d, *J* = 3.0 Hz, 2H), 4.90 (s, 4H), 3.78 (s, 4H), 2.95 (q, *J* = 6.5 Hz, 5H), 2.66 (s, 4H), 1.35 (s, 4H), 1.21 (s, 4H). **^{13}C NMR (126 MHz, DMSO-d$_6$)** δ 155.7, 152.1, 149.7, 111.1, 108.5, 57.3, 30.9, 29.3, 27.2, 25.9.

Polyurethane (**5e**) = The product was obtained as a white solid after precipitation with methanol (210 mg, 89%). **^{1}H NMR (400 MHz, DMSO-d$_6$)** δ 9.63 (s, 2H), 7.34 (s, 4H), 6.46 (d, *J* = 3.2 Hz, 2H), 6.26 (d, *J* = 3.2 Hz, 2H), 5.04 (s, 4H), 3.80 (s, 4H), 2.68 (s, 4H). **^{13}C NMR (101 MHz, DMSO-d$_6$)** δ 153.0, 152.4, 149.2, 133.8, 118.8, 111.7, 108.6, 57.7, 30.9, 27.2.

Polyurethane (5f) = The product was obtained as a white solid after precipitation with methanol (198 mg, 70%). **^{1}H NMR (400 MHz, DMSO-d$_6$)** δ 9.67 (s, 2H), 7.34 (d, *J* = 8.2 Hz, 4H), 7.08 (d, *J* = 8.6 Hz, 4H), 6.45 (d, *J* = 3.3 Hz, 2H), 6.25 (d, *J* = 3.2 Hz, 2H), 5.04 (s, 4H), 3.79 (s, 6H), 2.67 (s, 4H). **^{13}C NMR (101 MHz, DMSO-d$_6$)** δ 152.9, 152.4, 149.2, 136.9, 135.6, 128.9, 118.4, 111.7, 108.6, 57.7, 30.9, 27.2.

Polyurethane (**5g**) =. The product was obtained as a white solid after precipitation with methanol (230 mg, 72%). **^{1}H NMR (500 MHz, DMSO-d$_6$)** δ 7.34 (dd, *J* = 8.1, 2.4 Hz, 4H), 7.22 (dt, *J* = 8.3, 4.1 Hz, 6H), 6.35 (d, *J* = 3.1 Hz, 2H), 6.22 (d, *J* = 3.1 Hz, 2H), 4.88 (s, 4H), 4.25 (s, 4H), 2.93 (d, *J* = 6.8 Hz, 5H), 1.34 (s, 4H), 1.20 (s, 4H). **^{13}C NMR (101 MHz, DMSO-d$_6$)** δ 155.6, 150.9, 149.9, 135., 132.3, 131.2, 129.6, 111.2, 109.1, 57.3, 29.3, 25.9.

Polyurethane (**5h**) =. The product was obtained as a white solid after precipitation with methanol (215 mg, 67%). **^{1}H NMR (500 MHz, DMSO-d$_6$)** δ 9.64 (s, 2H), 7.38 – 7.30 (m, 8H), 7.25 – 7.19 (m, 4H), 6.43 (d, *J* = 3.3 Hz, 2H), 6.25 (d, *J* = 2.6 Hz, 2H), 5.02 (s, 4H), 4.27 (s, 4H). **^{13}C NMR (126 MHz, DMSO-d$_6$)** δ 153.0, 151.3, 149.4, 135.1, 133.8, 132.3, 131.2, 129.6, 118.8, 111.7, 109.2, 57.7, 29.2.

Polyurethane (5i) = The product was obtained as a white solid after precipitation with methanol (250 mg, 69%). **^{1}H NMR (400 MHz, DMSO-d$_6$)** δ 9.67 (s, 2H), 7.41 – 7.29 (m, 9H), 7.26 – 7.17 (m, 4H), 7.08 (d, *J* = 8.1 Hz, 4H), 6.43 (d, *J* = 3.1 Hz, 2H), 6.24 (d, *J* = 3.1 Hz, 2H), 5.03 (s, 4H), 4.27 (s, 4H), 3.77 (s, 2H). **^{13}C NMR (101 MHz, DMSO-d$_6$)** δ 152.9, 151.3, 149.4, 136.9, 135.6, 135.1, 132.4, 131.2, 129.6, 128.9, 118.4, 111.7, 109.2, 57.7, 29.3.

Polyurethane (**5j**) = The product was obtained as a white solid after precipitation with methanol (210 mg, 73%). **^{1}H NMR (400 MHz, DMSO-d$_6$)** δ 7.20 (d, *J* = 6.4 Hz, 2H), 6.37 (d, *J* = 3.1 Hz, 2H),

6.23 (d, J = 3.1 Hz, 2H), 4.90 (s, 4H), 3.78 (d, J = 4.8 Hz, 4H), 3.57 – 3.47 (m, 8H), 2.95 (d, J = 6.5 Hz, 4H), 2.62 (t, J = 6.6 Hz, 4H), 1.35 (s, 4H), 1.21 (s, 4H). **^{13}C NMR (101 MHz, DMSO-d_6)** δ 155.7, 152.2, 149.7, 111.1, 108.5, 69.9, 69.5, 57.3, 30.5, 29.3, 27.6, 25.9.

Polyurethane (**5k**) = The product was obtained as a white solid after precipitation with methanol (223 mg, 79%). **^{1}H NMR (400 MHz, DMSO-d_6)** δ 9.63 (s, 2H), 7.34 (s, 4H), 6.45 (d, J = 3.1 Hz, 2H), 6.26 (d, J = 3.2 Hz, 2H), 5.04 (s, 4H), 3.79 (d, J = 2.7 Hz, 4H), 3.57 – 3.42 (m, 8H), 2.65 – 2.58 (m, 4H). **^{13}C NMR (101 MHz, DMSO-d_6)** δ 153.03, 152.53, 149.15, 133.84, 118.77, 111.63, 108.55, 69.96, 69.47, 57.71, 30.48, 27.63.

Polyurethane (5l) = The product was obtained as a white solid after precipitation with methanol (200 mg, 61%). **^{1}H NMR (400 MHz, DMSO-d_6)** δ 9.67 (s, 2H), 7.34 (d, J = 8.1 Hz, 4H), 7.08 (d, J = 8.9 Hz, 4H), 6.45 (d, J = 3.2 Hz, 2H), 6.25 (d, J = 3.3 Hz, 2H), 5.04 (s, 4H), 3.79 (s, 6H), 3.54 – 3.42 (m, 8H), 2.61 (t, J = 6.5 Hz, 4H). **^{13}C NMR (101 MHz, DMSO-d_6)** δ 152.97, 152.54, 149.11, 136.86, 135.56, 128.84, 118.31, 111.64, 108.52, 69.95, 69.46, 57.71, 30.48, 27.62.

5. **Copies of the RMN spectral of the polyurethanes (5)**

^{1}H NMR (500 MHz, DMSO-d$_6$) Polyurethane (5a) obtained using 18 h of reaction

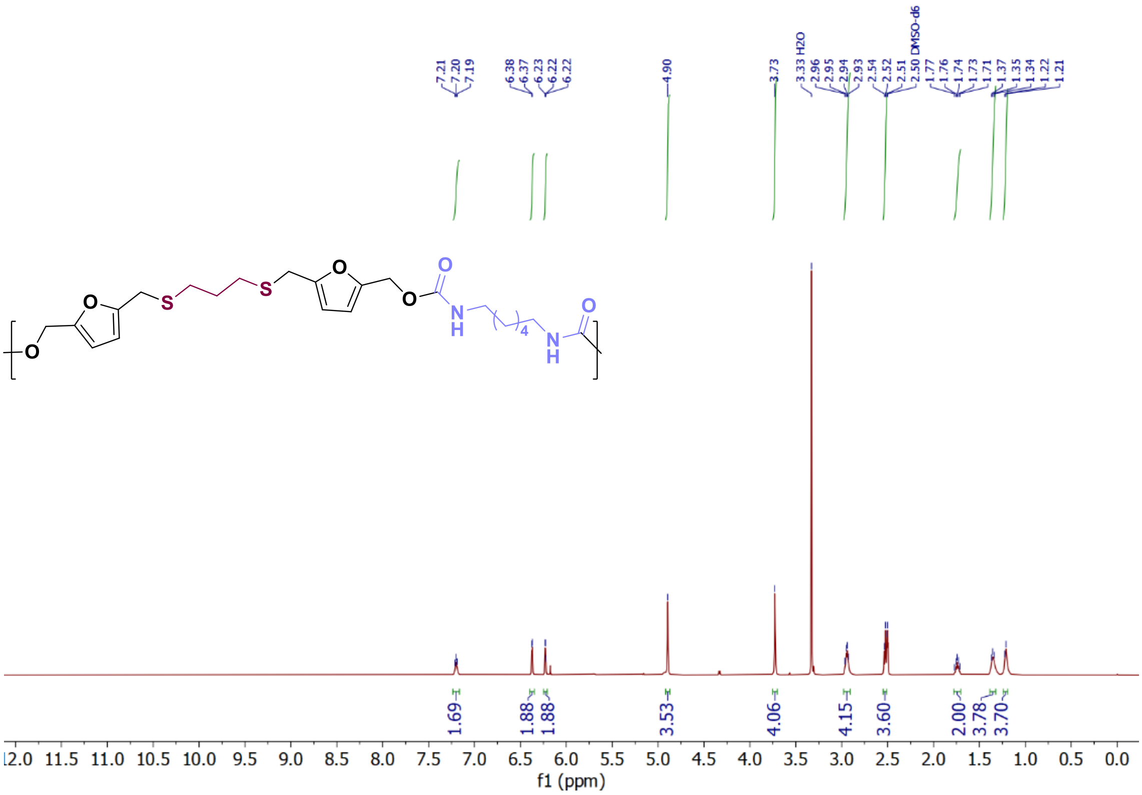

^{1}H NMR (500 MHz, DMSO-d$_6$) Polyurethane (5a) obtained using 0.5 h of reaction

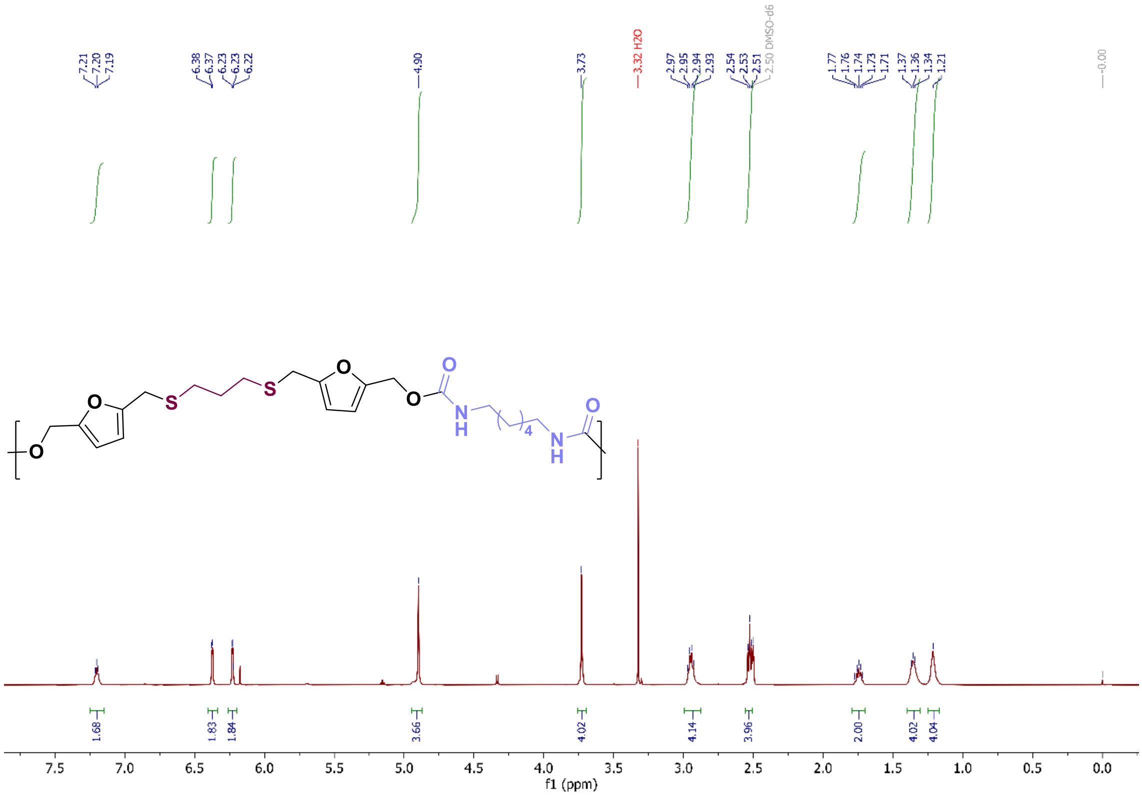

^{13}C NMR (126 MHz, DMSO-d$_6$) Polyurethane (5a) obtained using 18 h of reaction

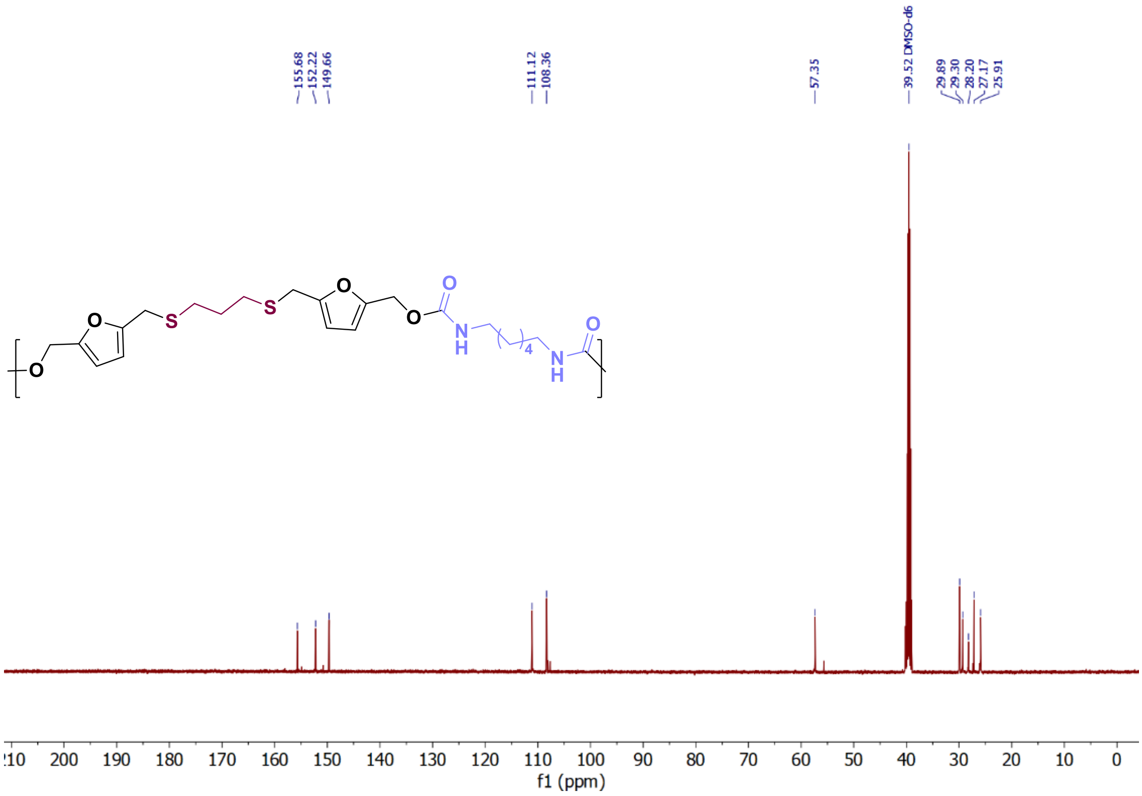

^{13}C NMR (126 MHz, DMSO-d$_6$) Polyurethane (5a) obtained using 0.5 h of reaction

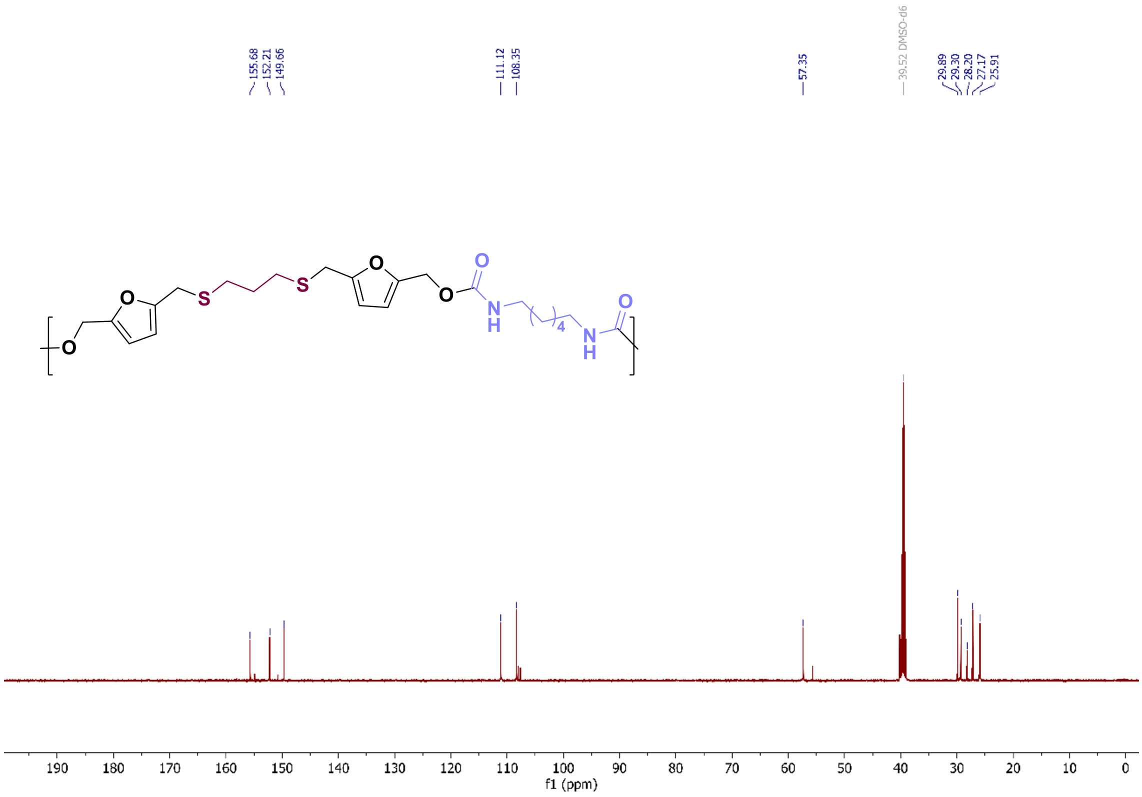

^1H NMR (500 MHz, DMSO-d_6) Polyurethane (5b) obtained using 18 h of reaction

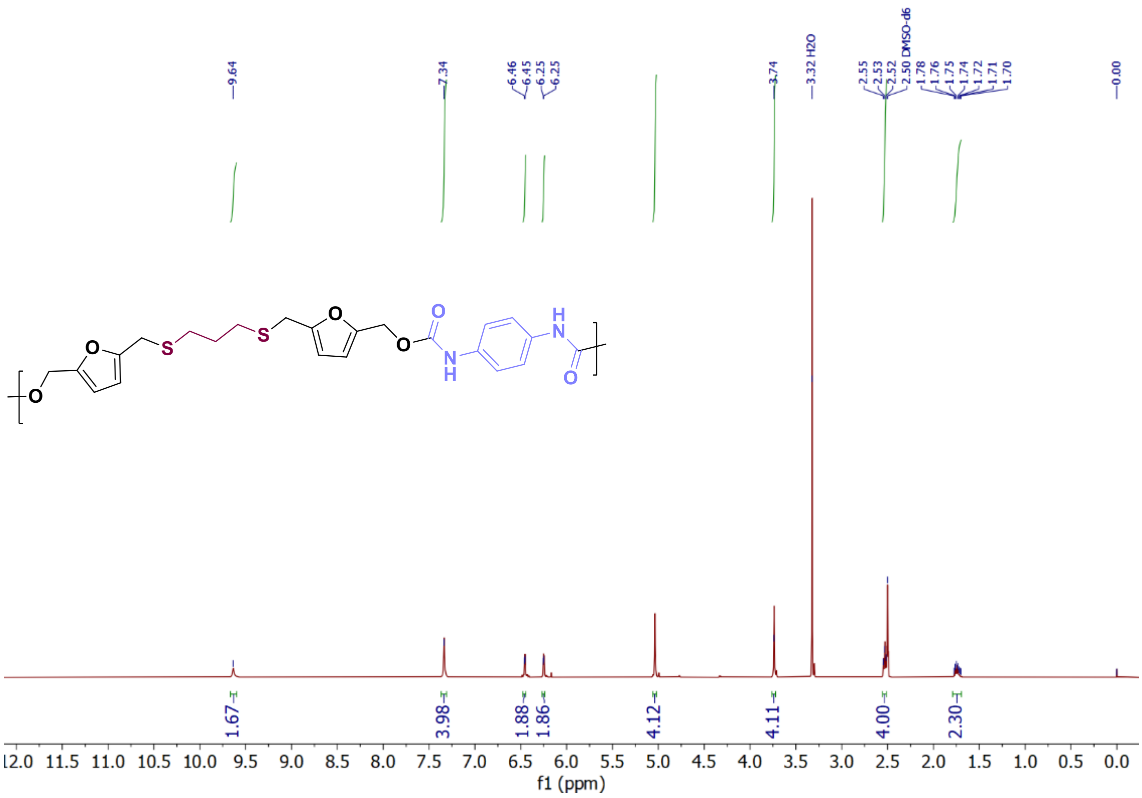

^1H NMR (500 MHz, DMSO-d_6) Polyurethane (5b) obtained using 0.5 h of reaction

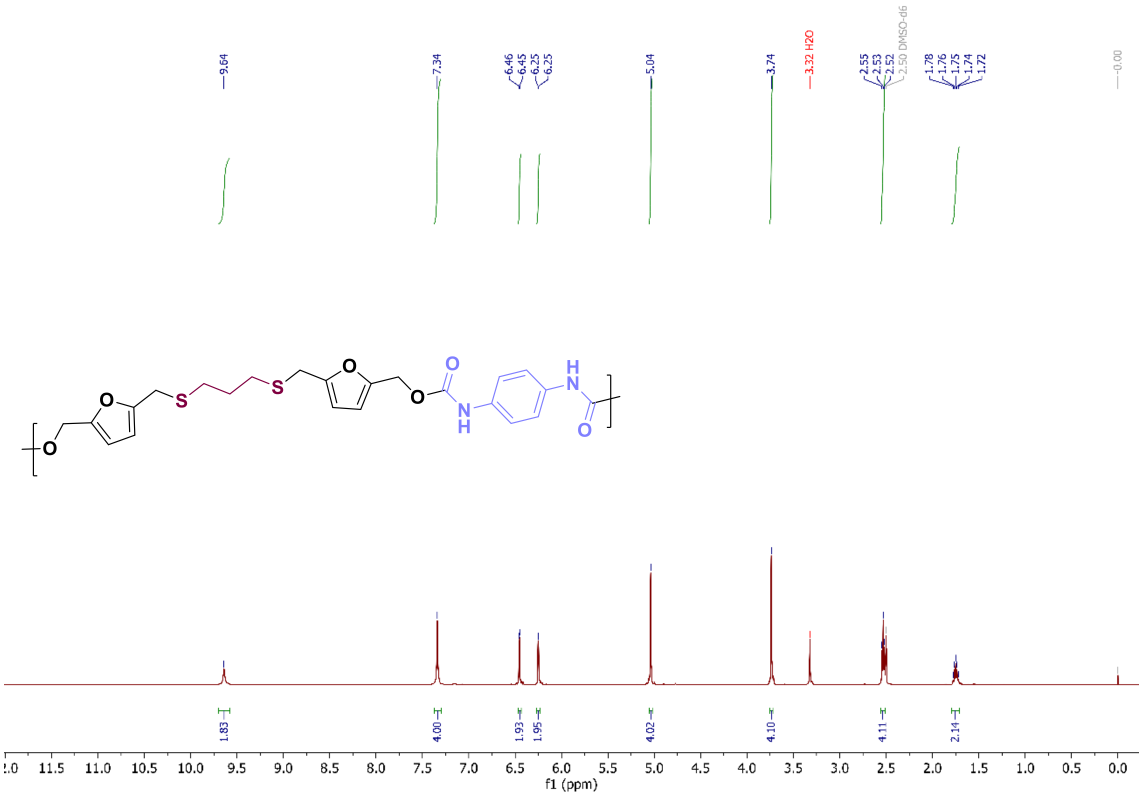

^{13}C NMR (126 MHz, DMSO-d$_6$) Polyurethane (5b) obtained using 18 h of reaction

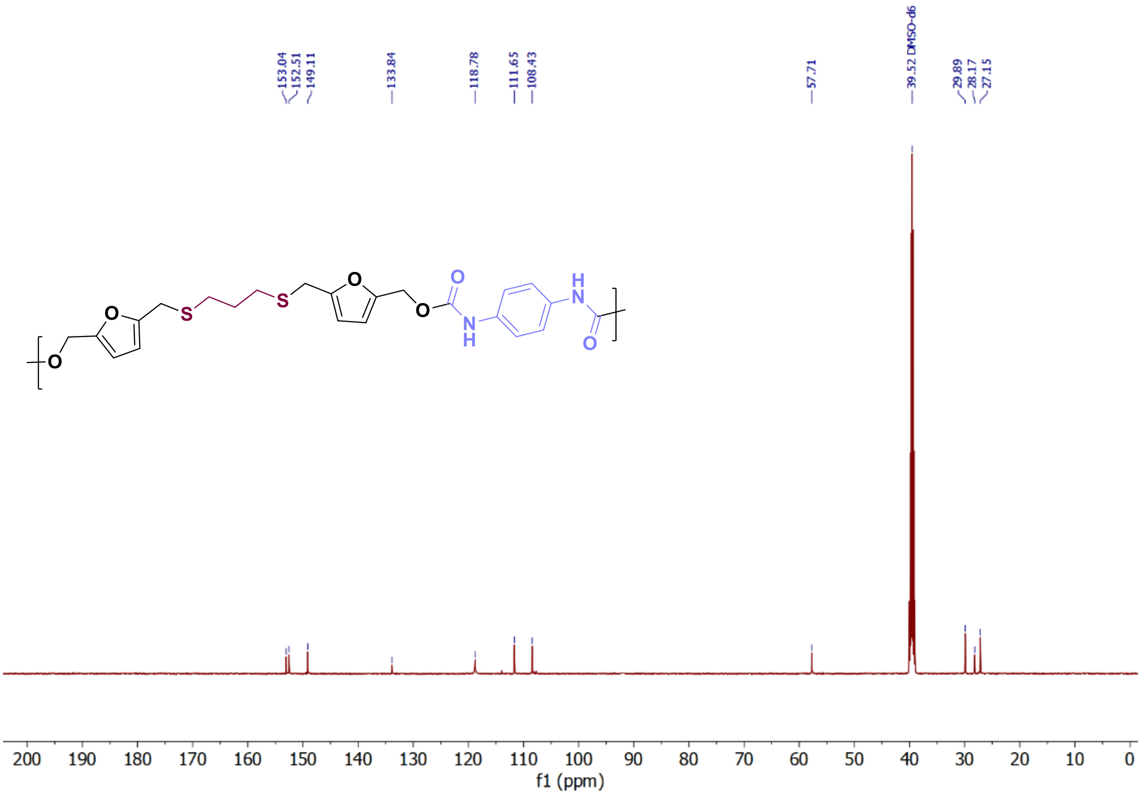

^{13}C NMR (126 MHz, DMSO-d$_6$) Polyurethane (5b) obtained using 18 h of reaction

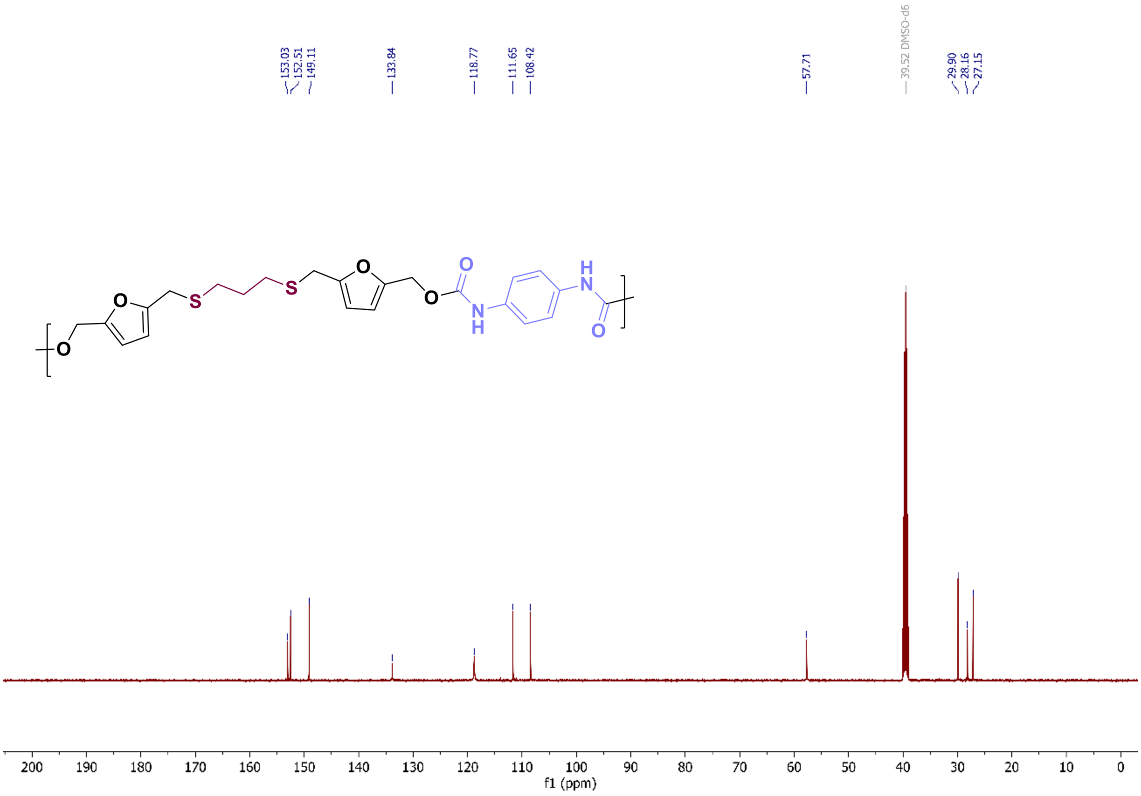

^{1}H NMR (500 MHz, DMSO-d$_6$) Polyurethane (5c) obtained using 18 h of reaction

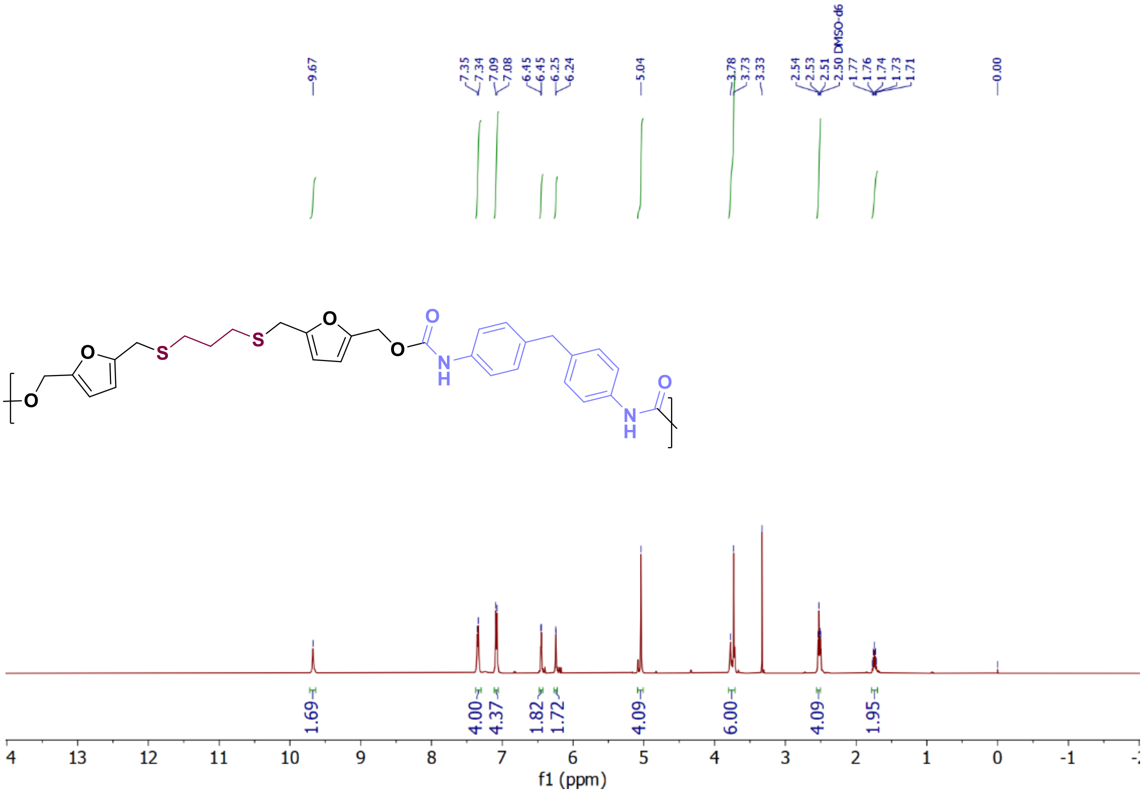

^{1}H NMR (400 MHz, DMSO-d$_6$) Polyurethane (5c) obtained using 0.5 h of reaction

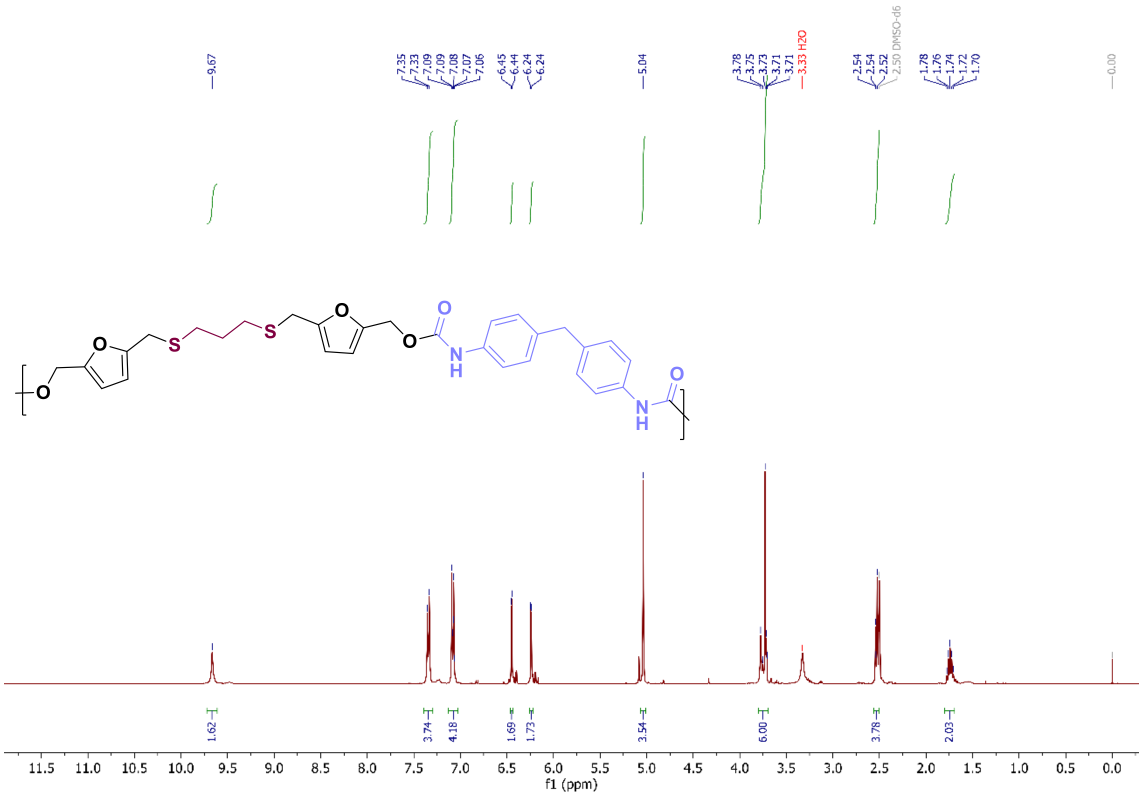

^{13}C NMR (126 MHz, DMSO-d_6) Polyurethane (5c) obtained using 18 h of reaction

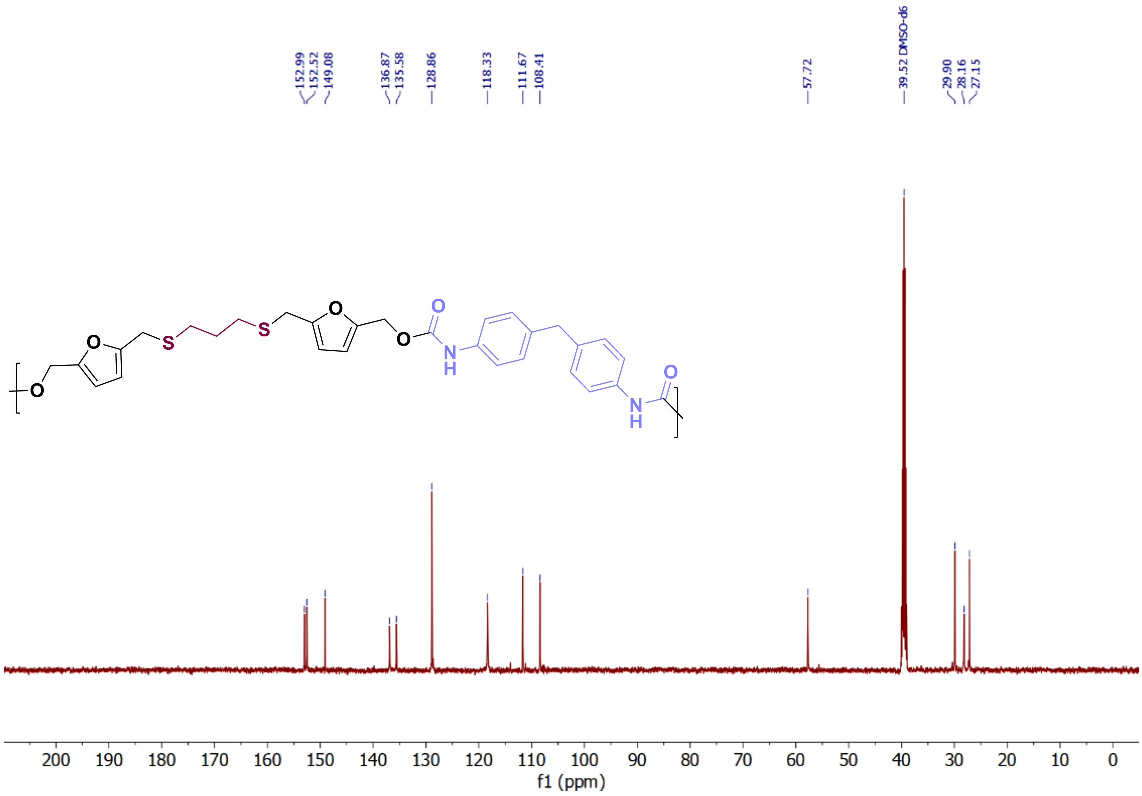

^{13}C NMR (101 MHz, DMSO-d_6) Polyurethane (5c) obtained using 0.5 h of reaction

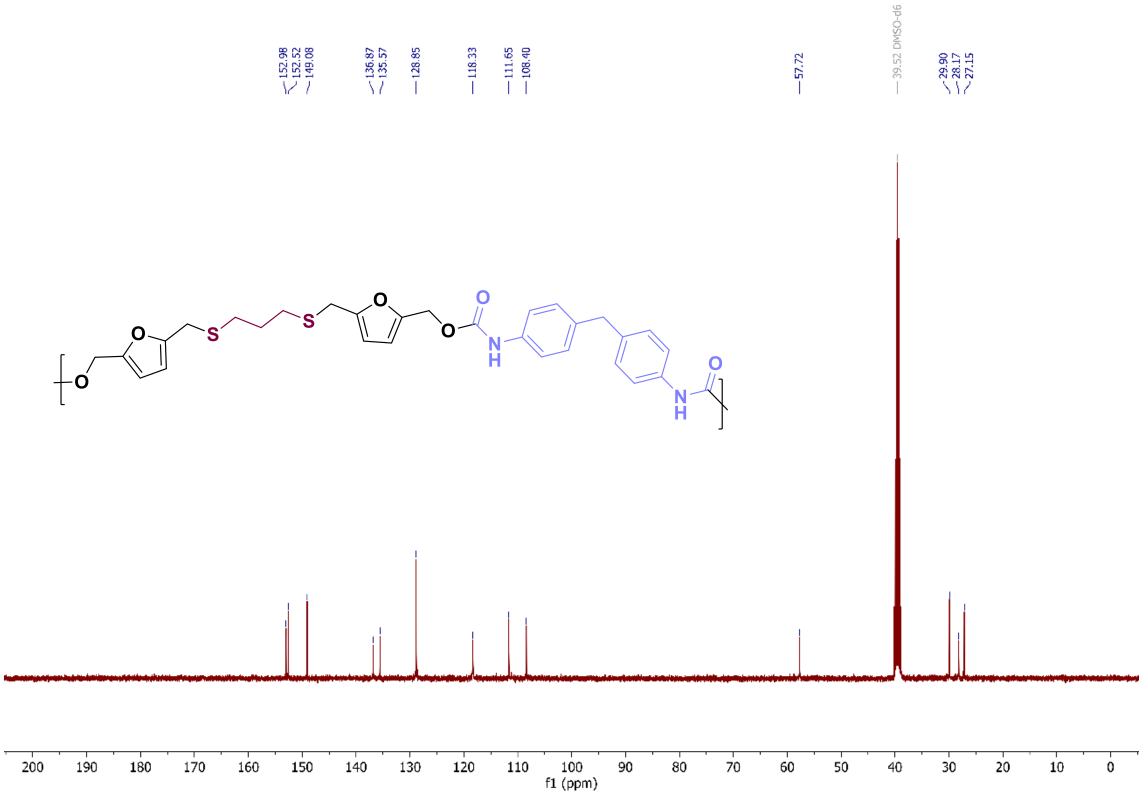

^1H NMR (500 MHz, DMSO-d_6) Polyurethane (**5d**) obtained using 18 h of reaction

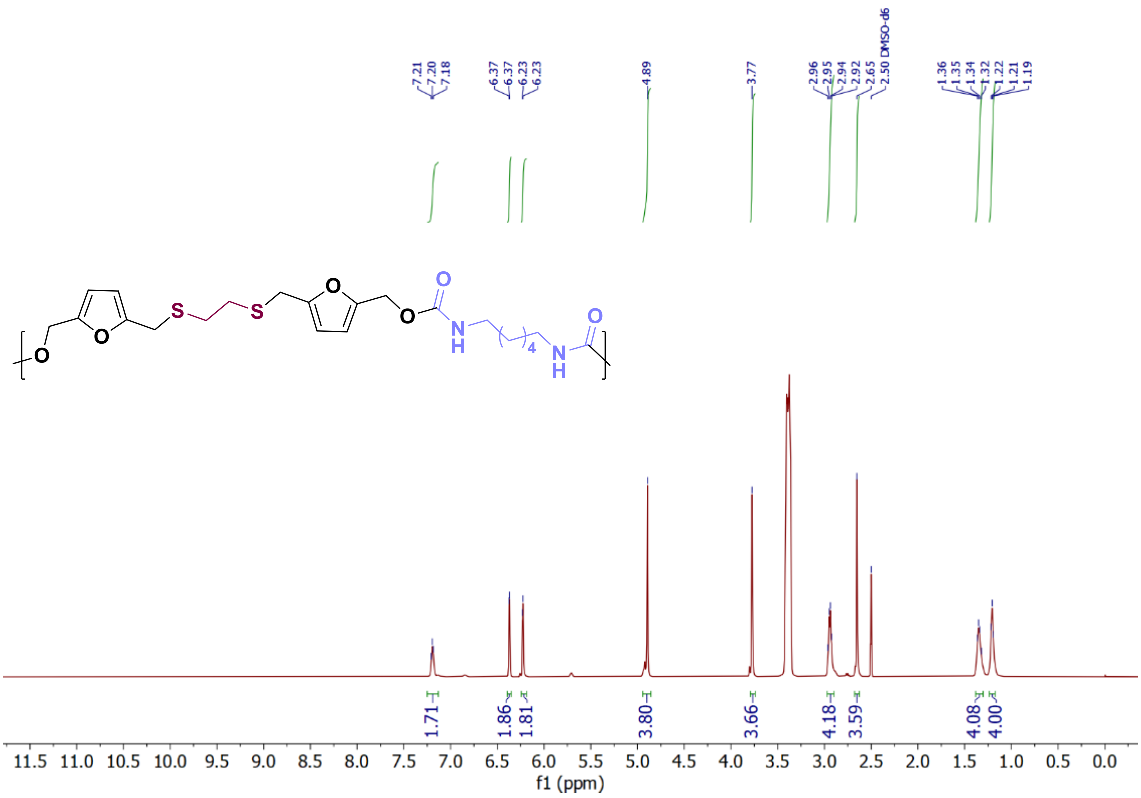

^1H NMR (500 MHz, DMSO-d_6) Polyurethane (**5d**) obtained using 0.5 h of reaction

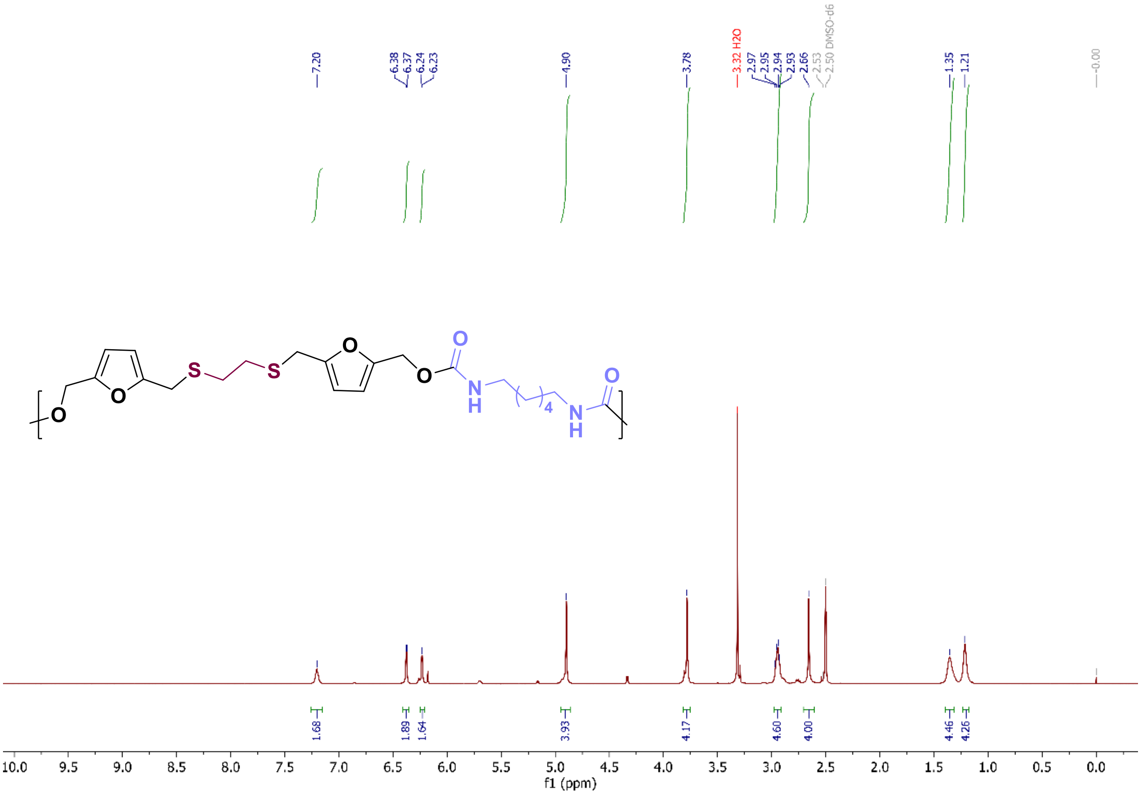

^{13}C NMR (126 MHz, DMSO-d_6) Polyurethane (5d) obtained using 18 h of reaction

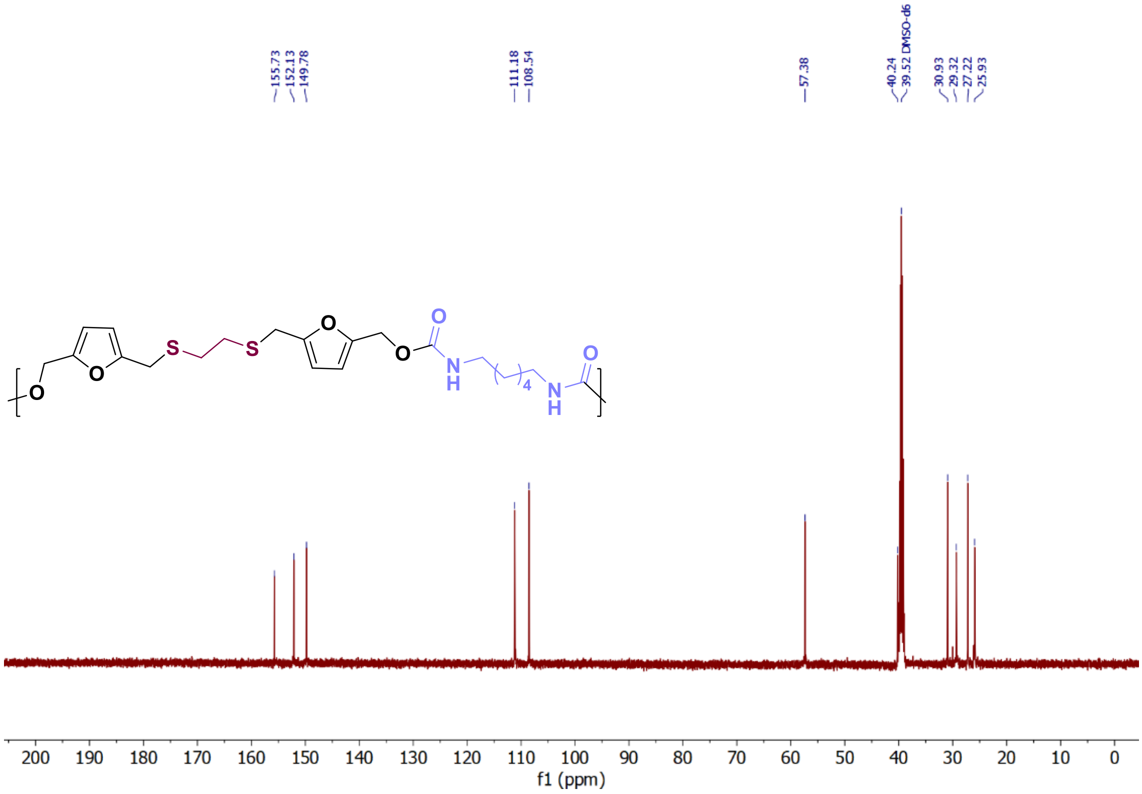

^{13}C NMR (126 MHz, DMSO-d_6) Polyurethane (5d) obtained using 0.5 h of reaction

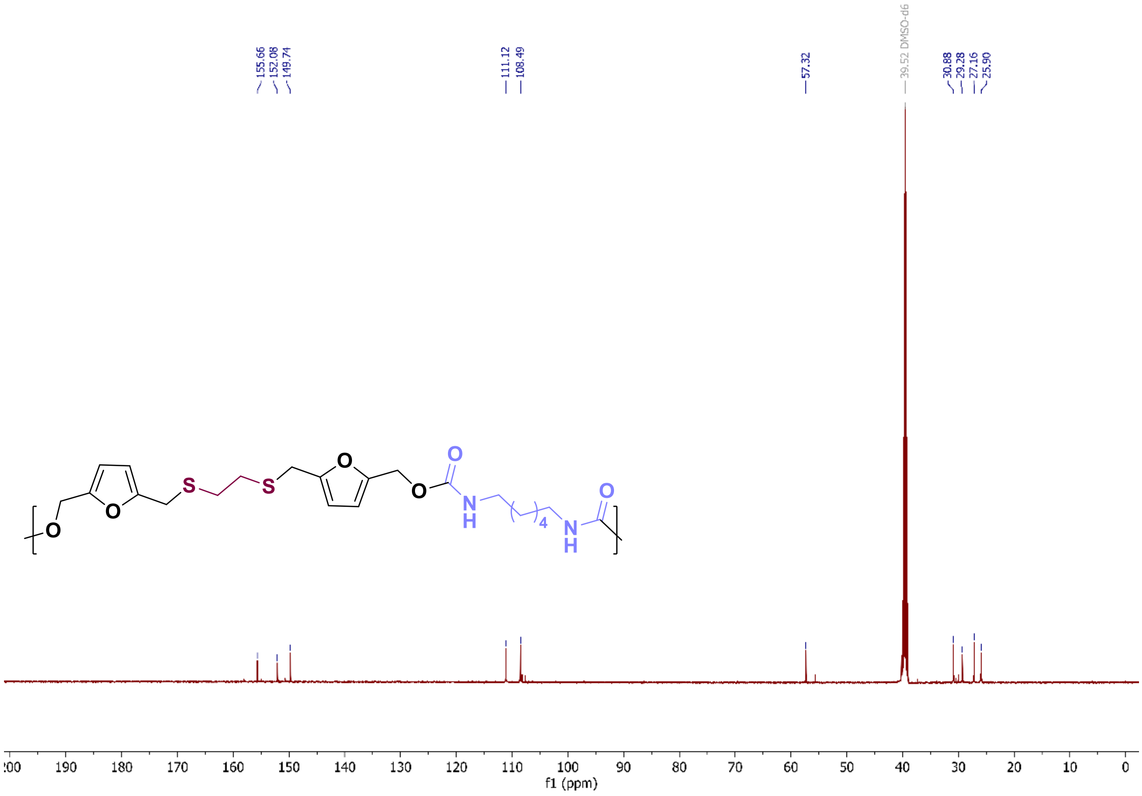

^{1}H NMR (500 MHz, DMSO-d$_6$) Polyurethane (5e) obtained using 18 h of reaction

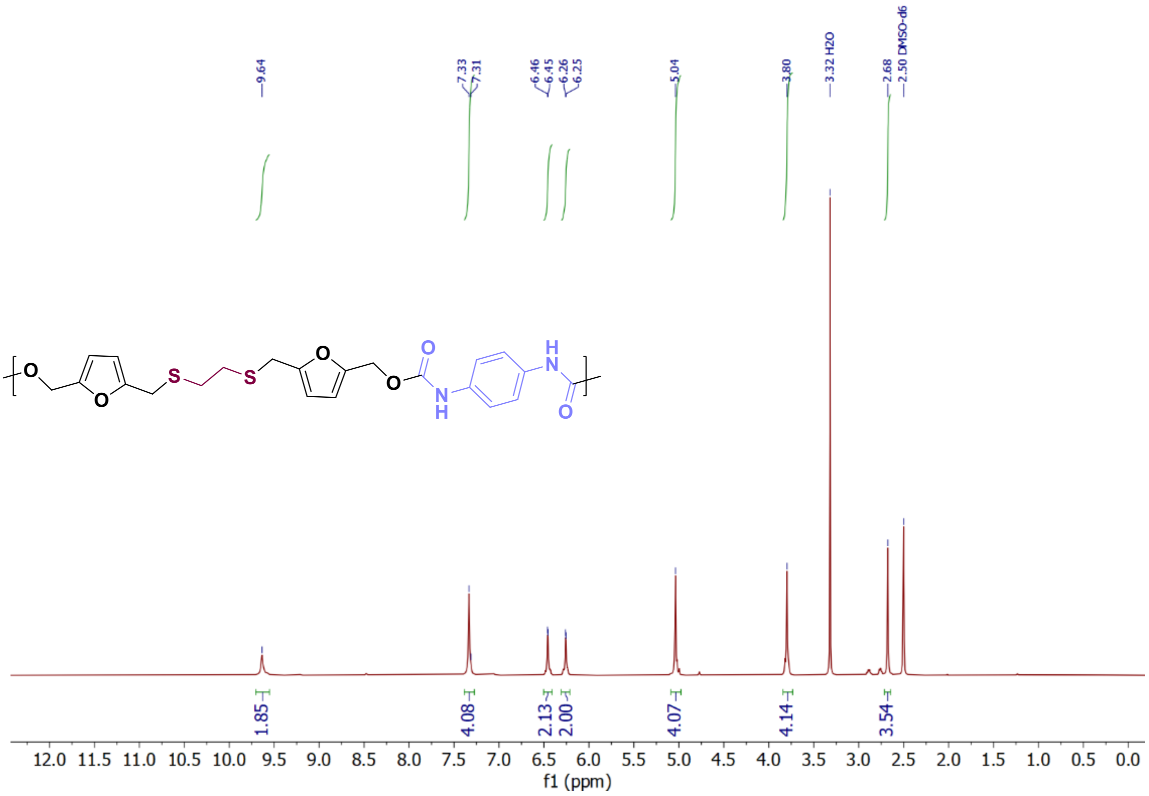

^{1}H NMR (400 MHz, DMSO-d$_6$) Polyurethane (5e) obtained using 0.5 h of reaction

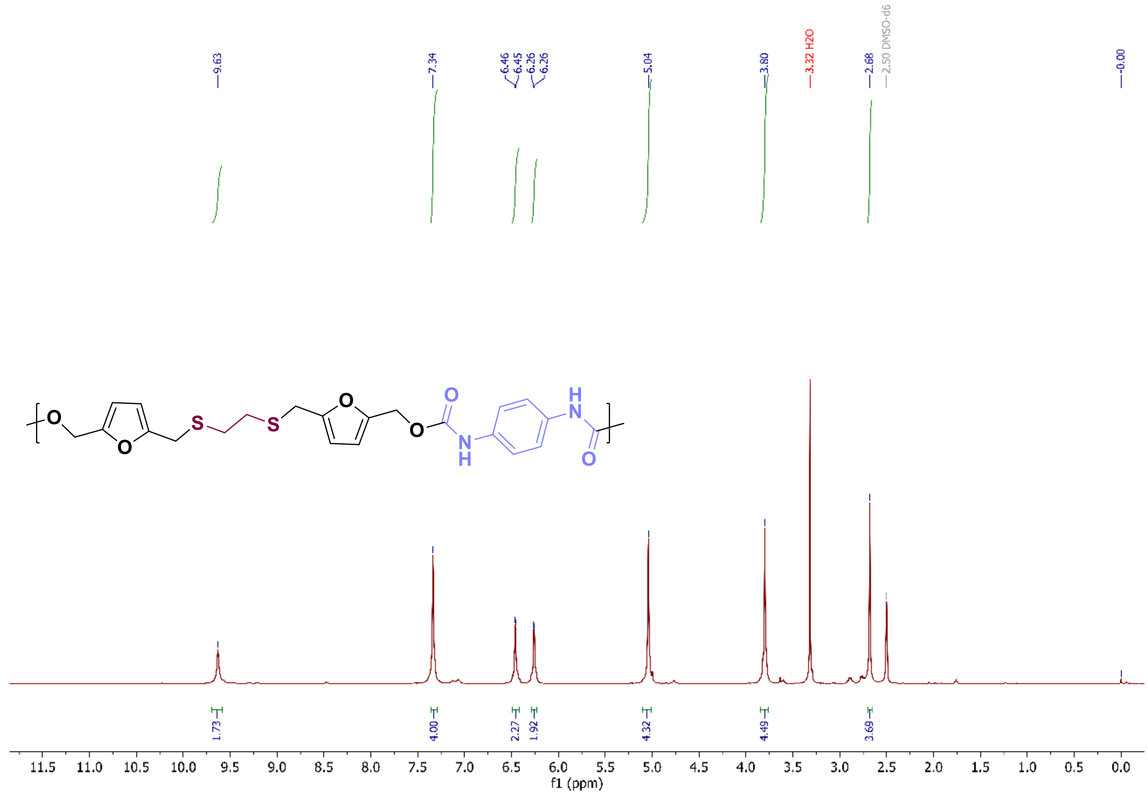

^{13}C NMR (126 MHz, DMSO-d_6) Polyurethane (5e) obtained using 18 h of reaction

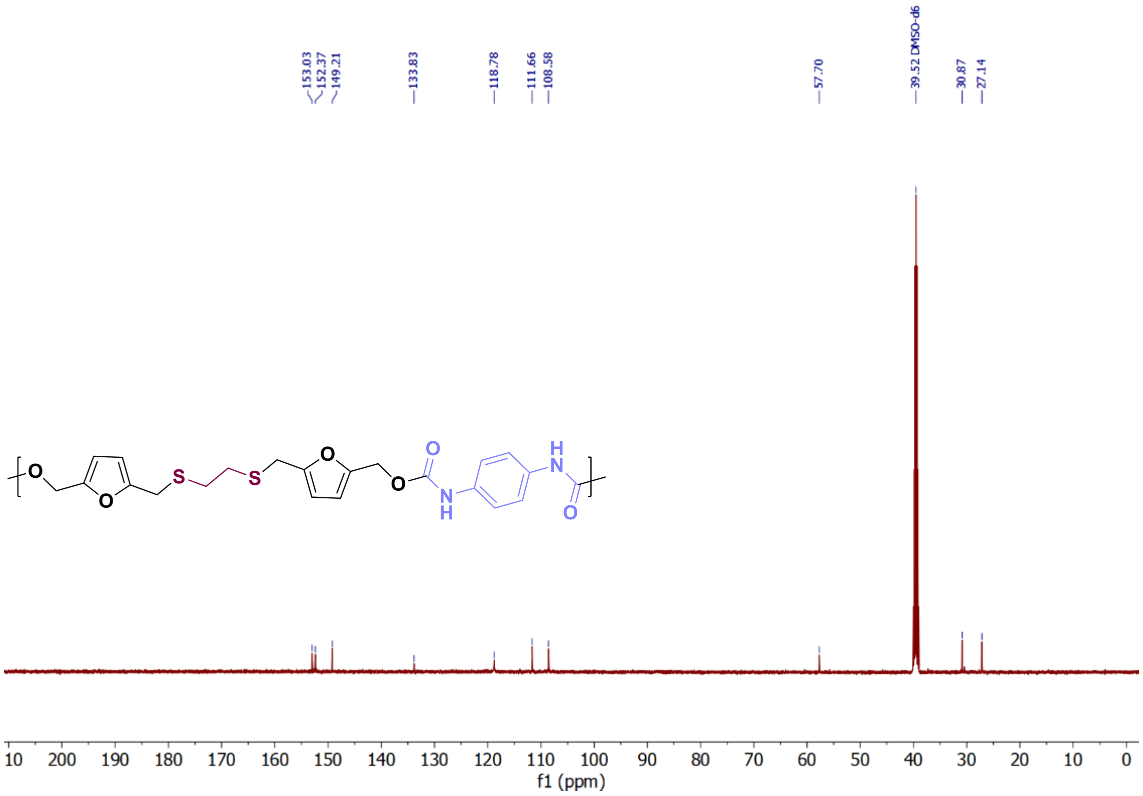

^{13}C NMR (101 MHz, DMSO-d_6) Polyurethane (5e) obtained using 0.5 h of reaction

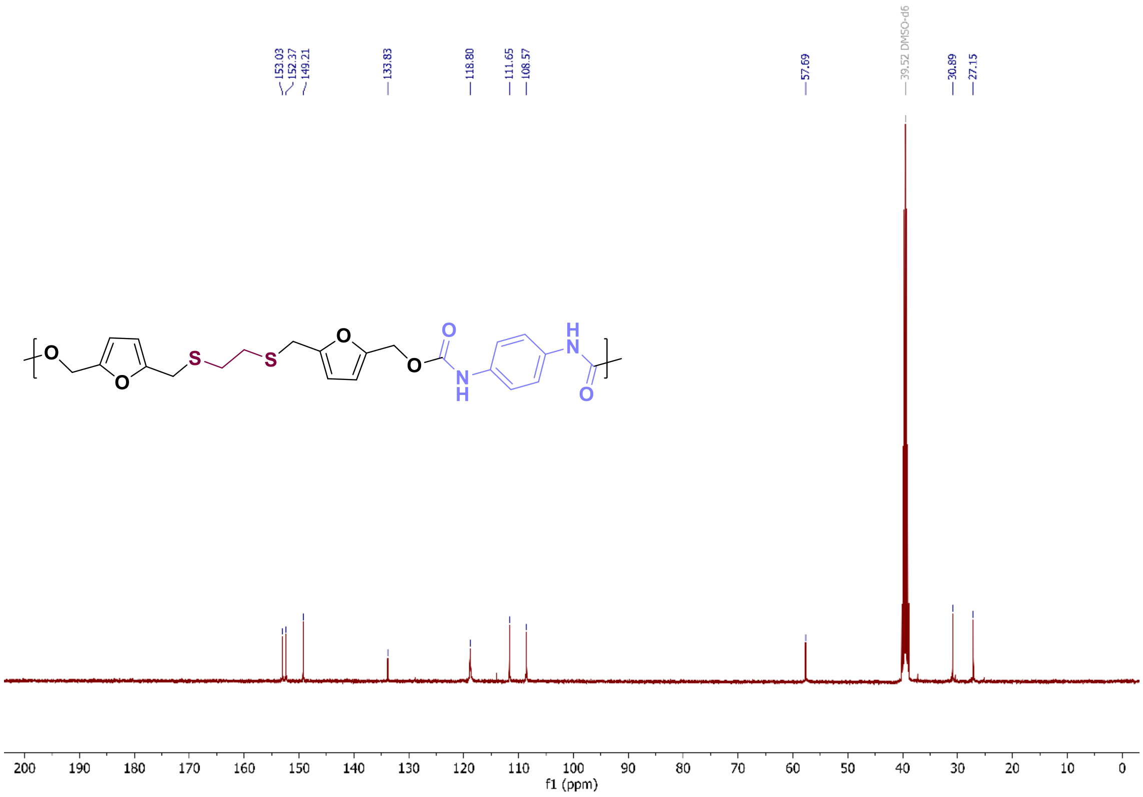

^{1}H NMR (500 MHz, DMSO-d$_6$) Polyurethane (5f) obtained using 18 h of reaction

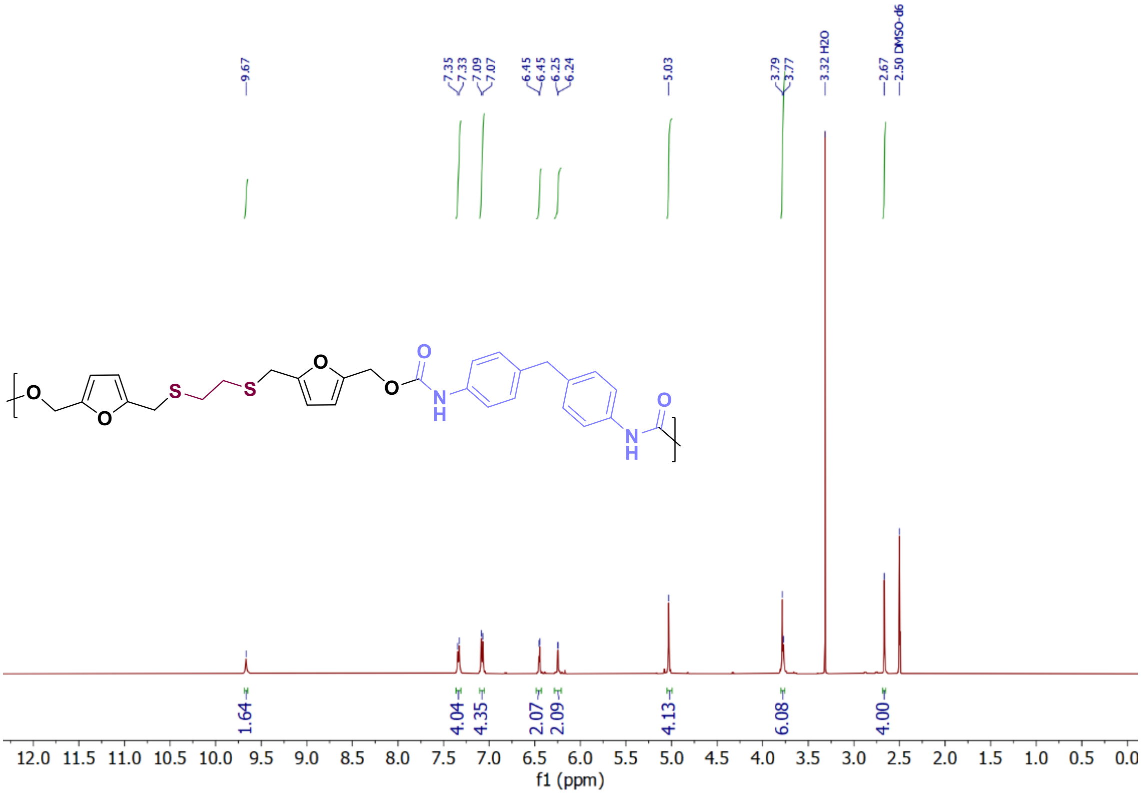

^{1}H NMR (400 MHz, DMSO-d$_6$) Polyurethane (5f) obtained using 0.5 h of reaction

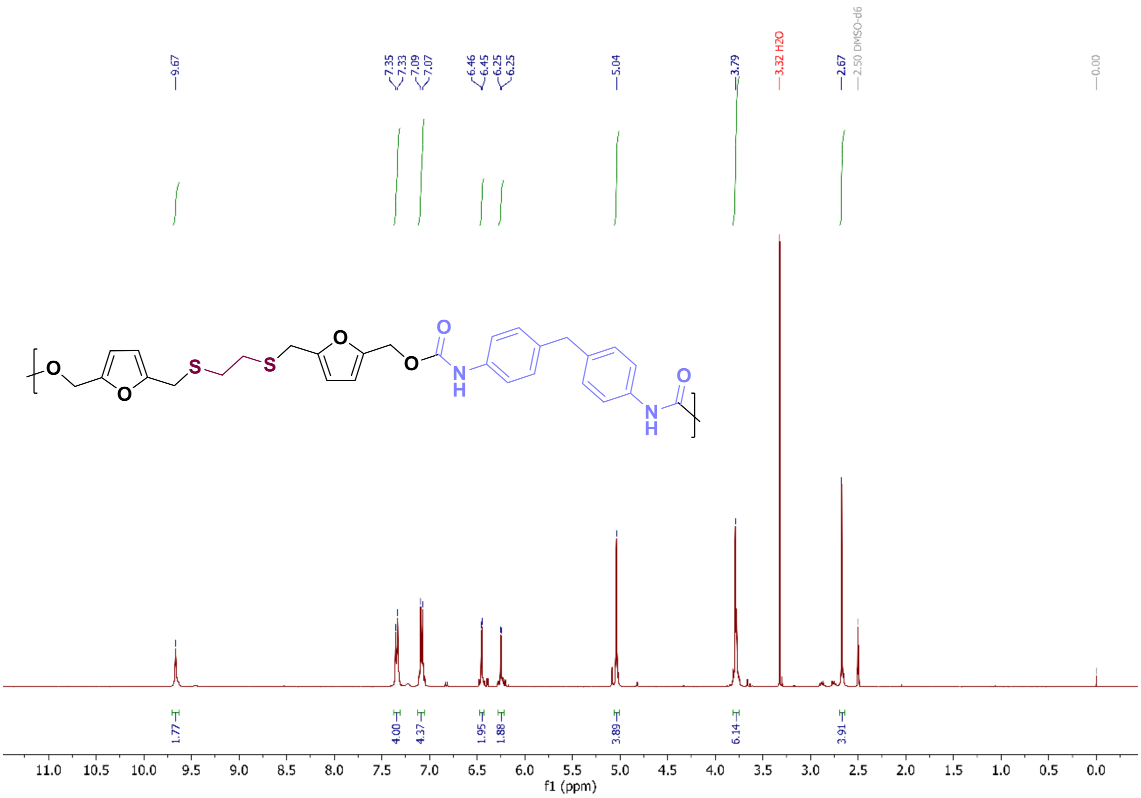

^{13}C NMR (126 MHz, DMSO-d_6) Polyurethane (5f) obtained using 18 h of reaction

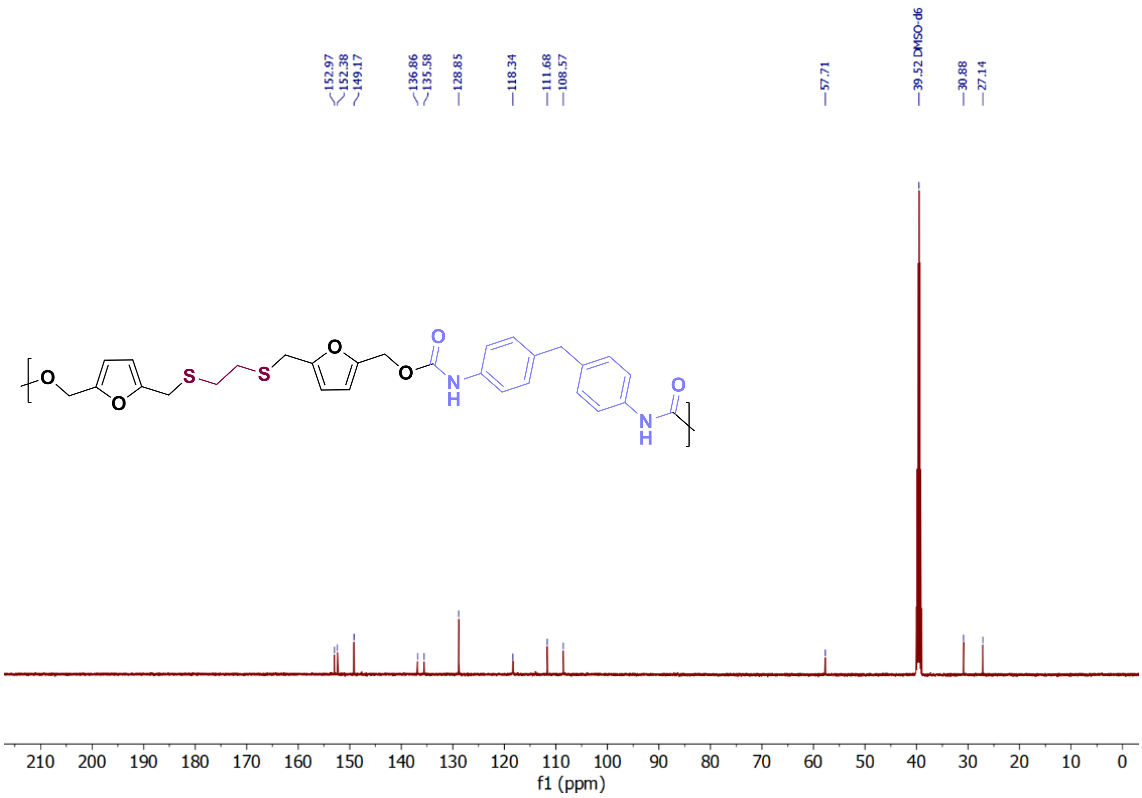

^{13}C NMR (101 MHz, DMSO-d_6) Polyurethane (5f) obtained using 0.5 h of reaction

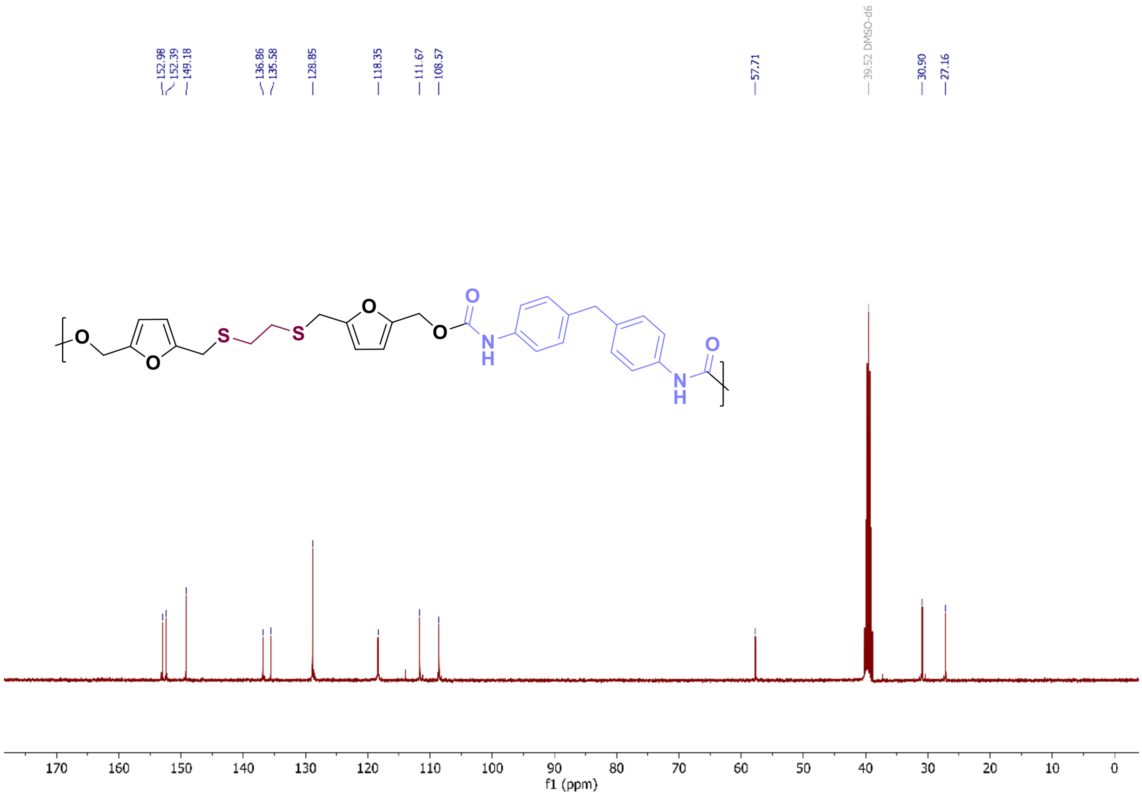

^{1}H NMR (500 MHz, DMSO-d$_6$) Polyurethane (5g) obtained using 18 h of reaction

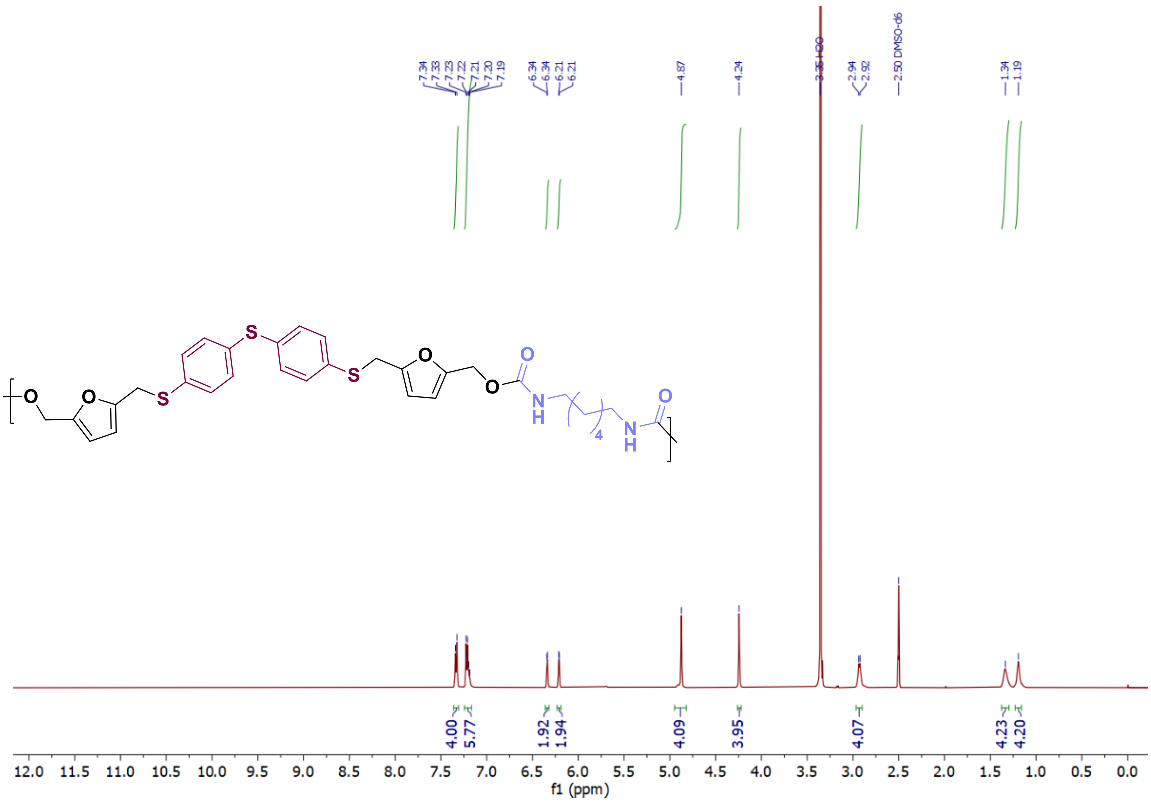

^{1}H NMR (500 MHz, DMSO-d$_6$) Polyurethane (5g) obtained using 0.5 h of reaction

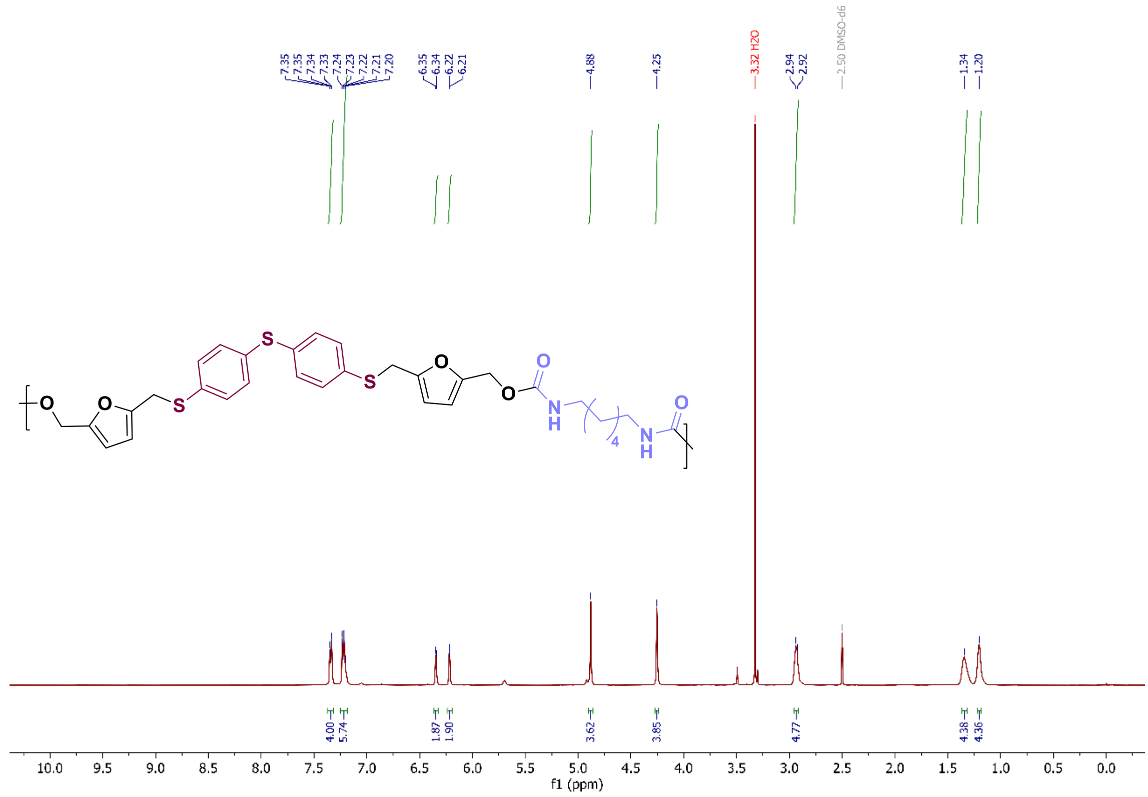

^{13}C NMR (126 MHz, DMSO-d_6) Polyurethane (5g) obtained using 18 h of reaction

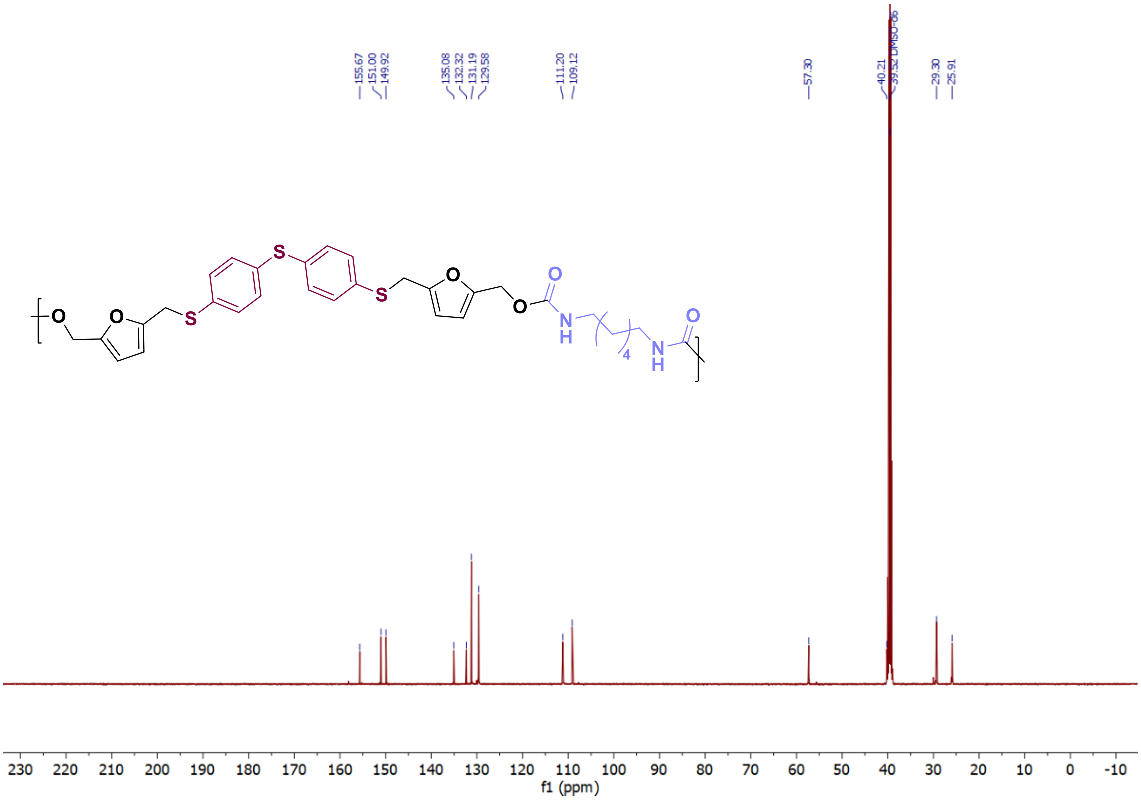

^{13}C NMR (126 MHz, DMSO-d_6) Polyurethane (5g) obtained using 0.5 h of reaction

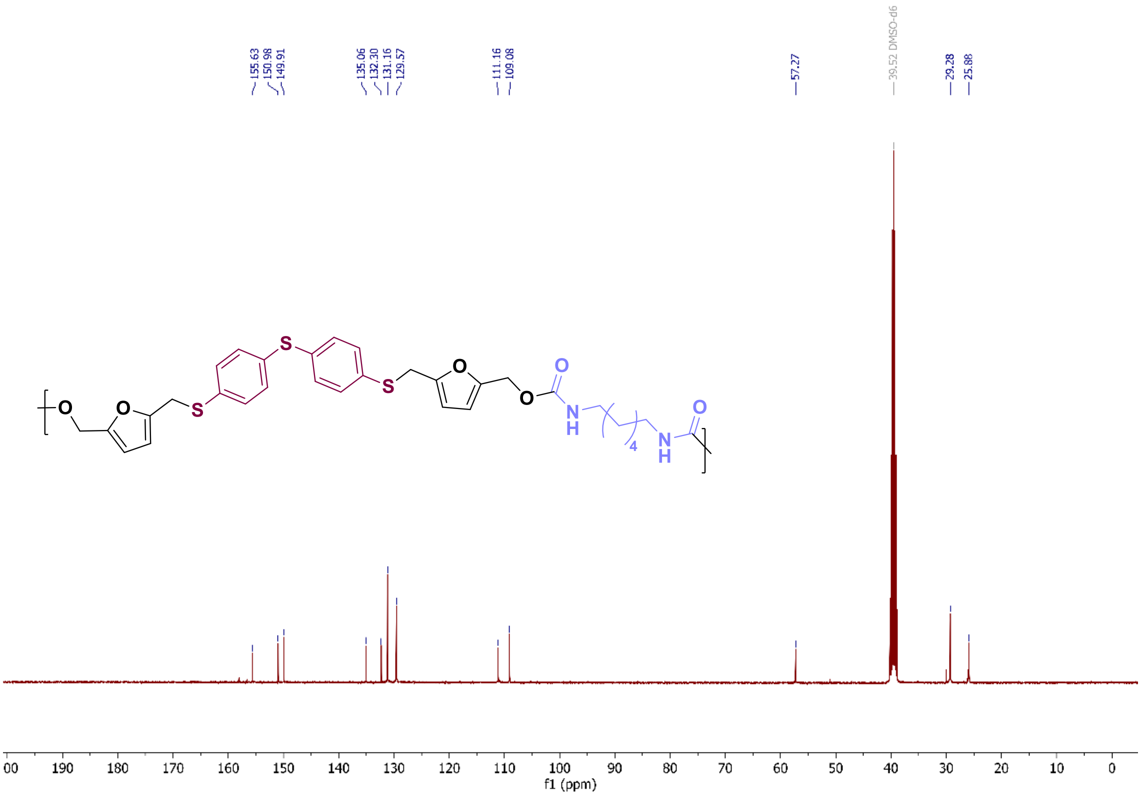

^{1}H NMR (500 MHz, DMSO-d$_6$) Polyurethane (5h) obtained using 18 h of reaction

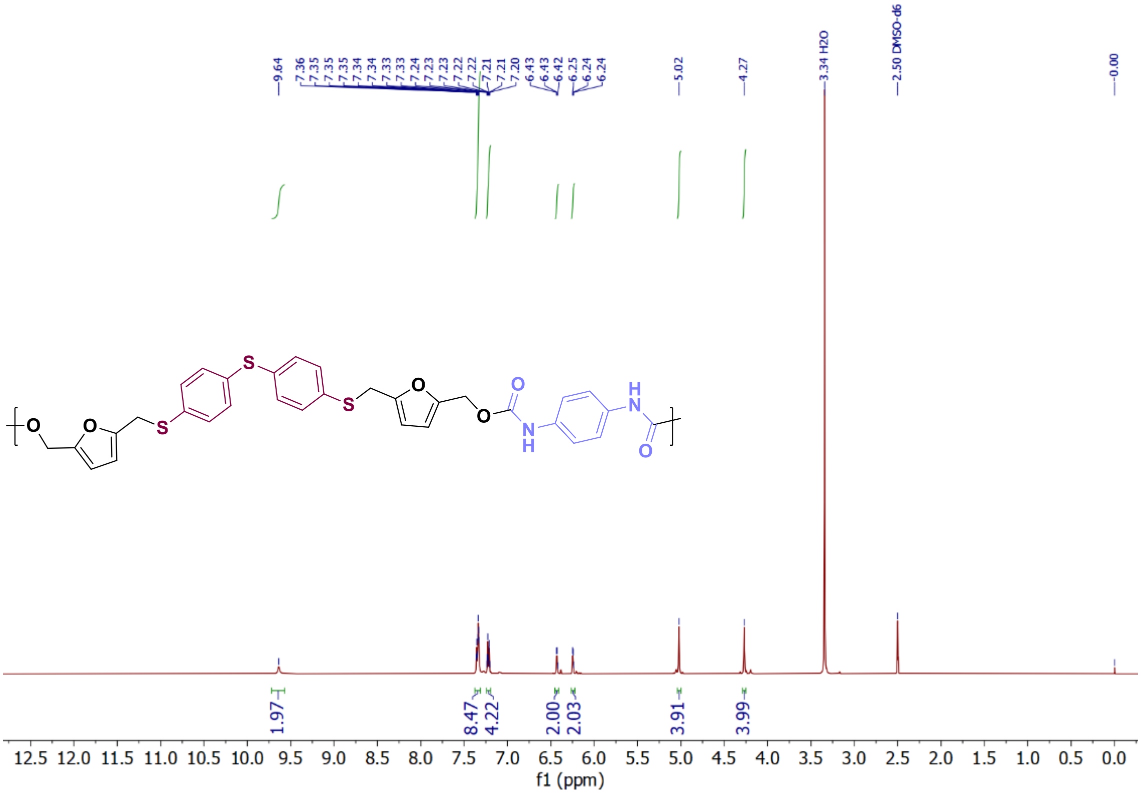

^{1}H NMR (500 MHz, DMSO-d$_6$) Polyurethane (5h) obtained using 0.5 h of reaction

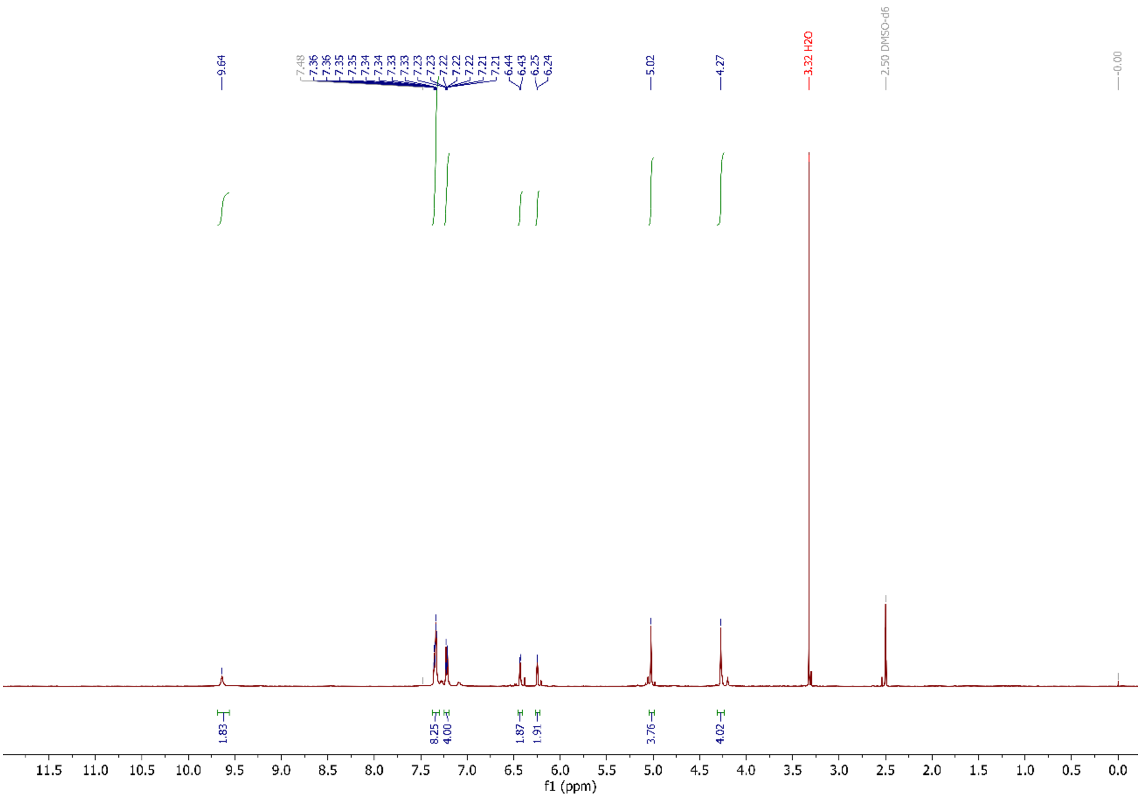

^{13}C NMR (126 MHz, DMSO-d_6) Polyurethane (5h) obtained using 18 h of reaction

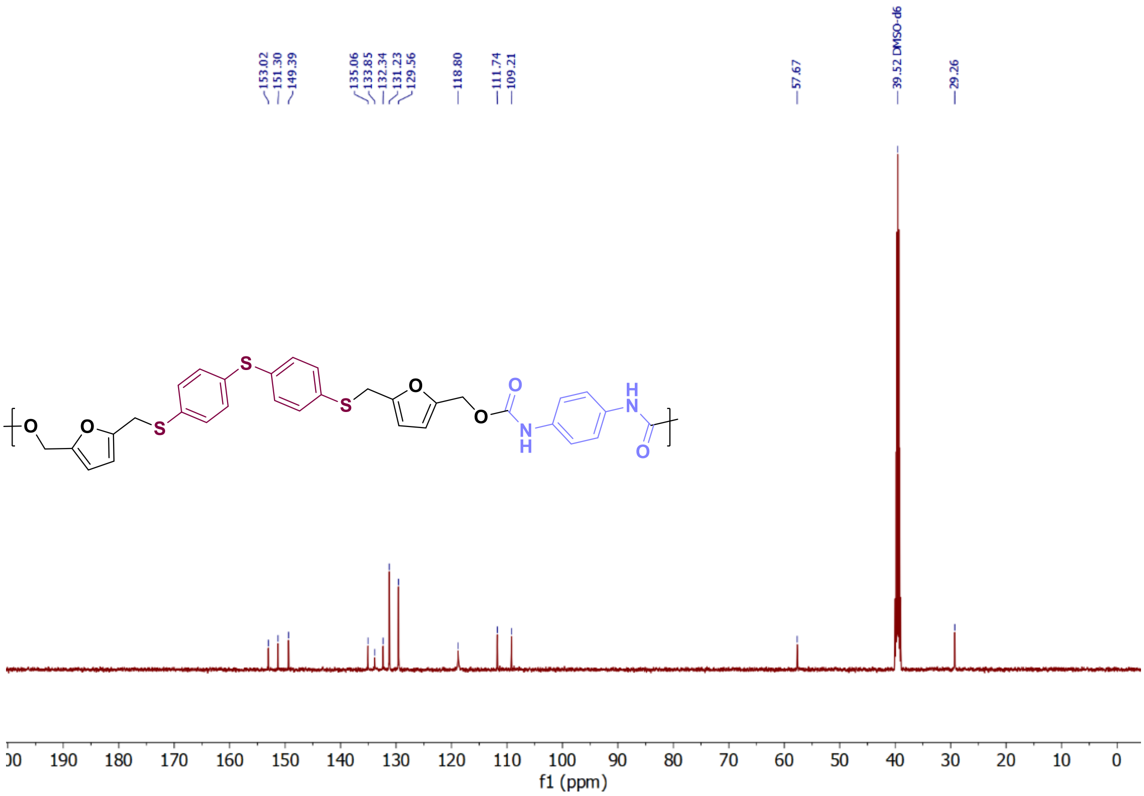

^{13}C NMR (126 MHz, DMSO-d_6) Polyurethane (5h) obtained using 0.5 h of reaction

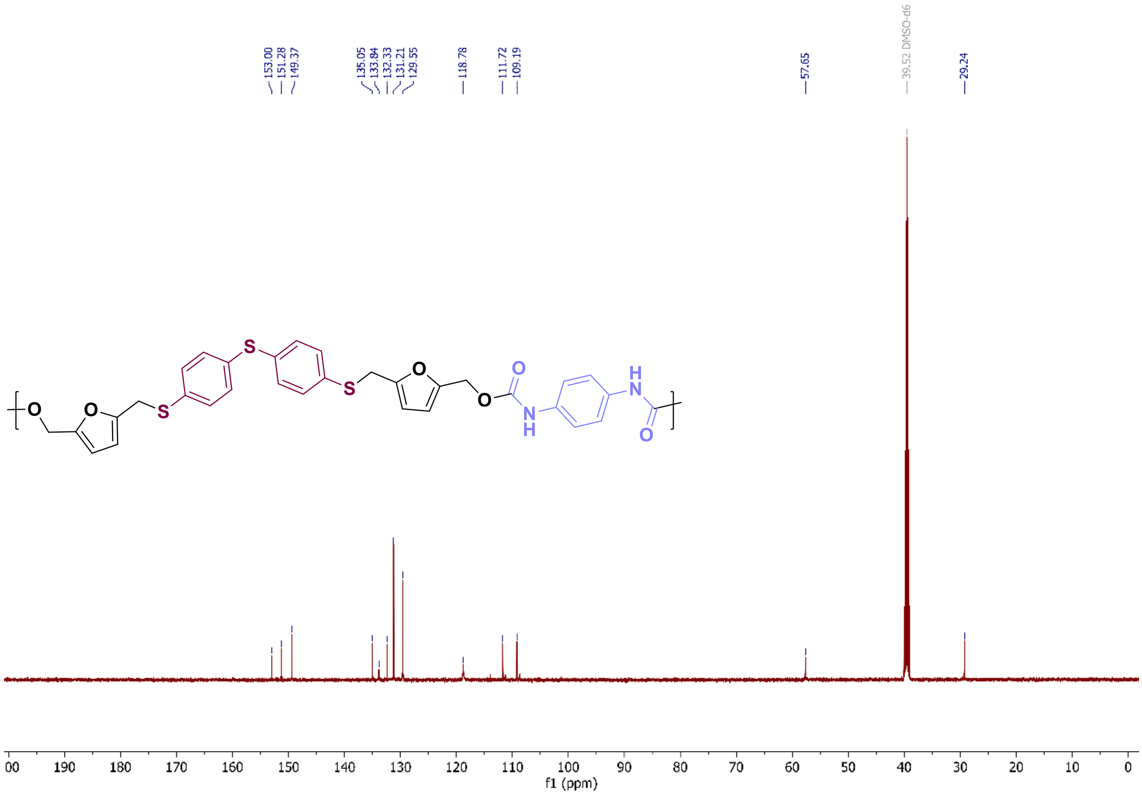

^{1}H NMR (500 MHz, DMSO-d$_6$) Polyurethane (5i) obtained using 18 h of reaction

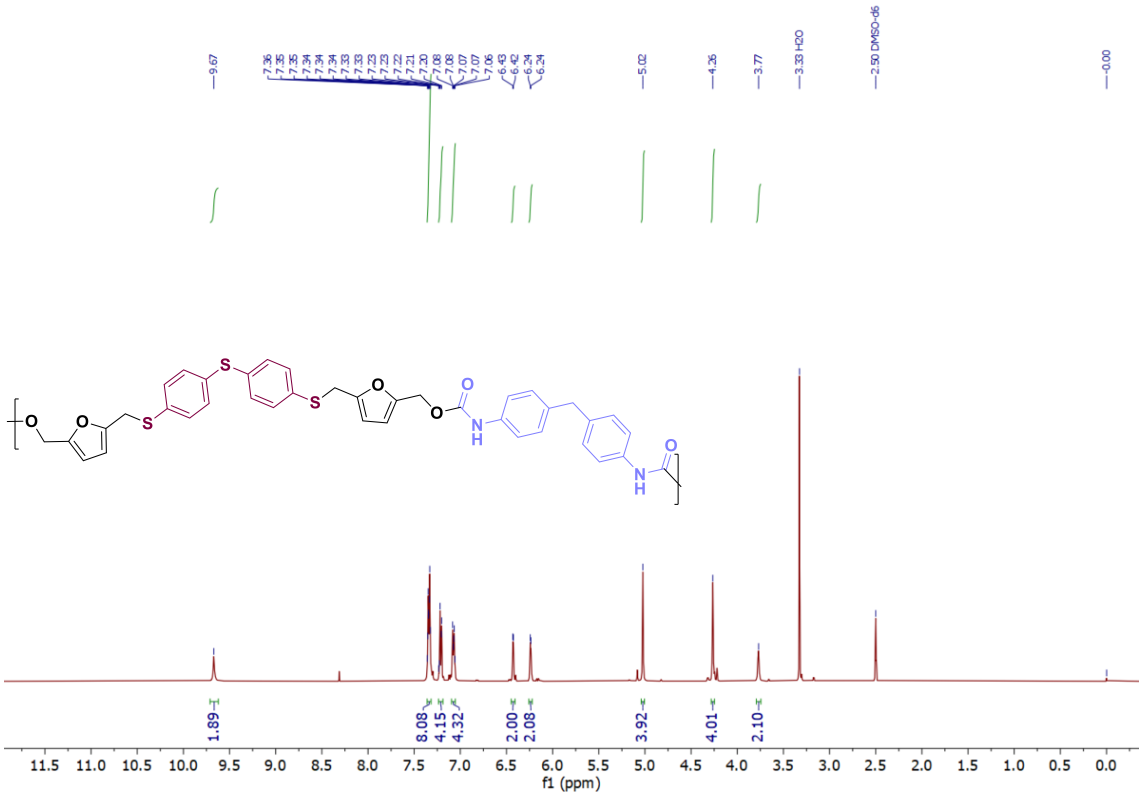

^{1}H NMR (400 MHz, DMSO-d$_6$) Polyurethane (5i) obtained using 0.5 h of reaction

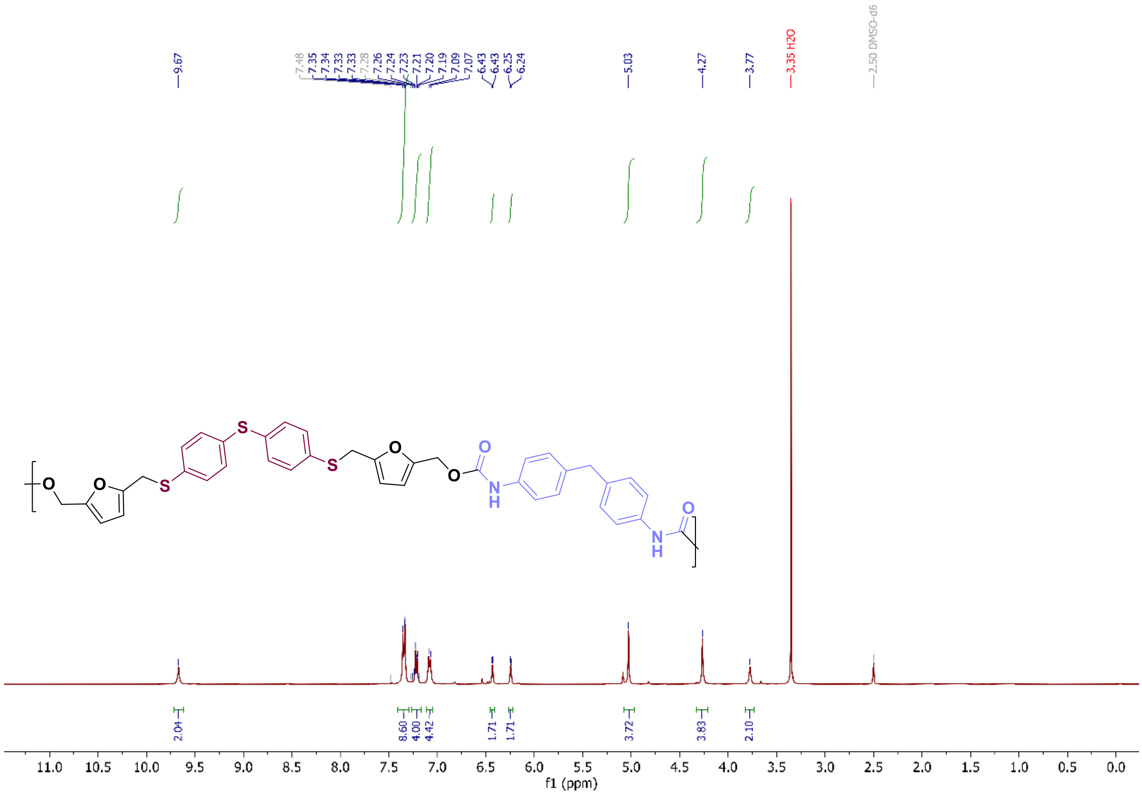

^{13}C NMR (126 MHz, DMSO-d_6) Polyurethane (5i) obtained using 18 h of reaction

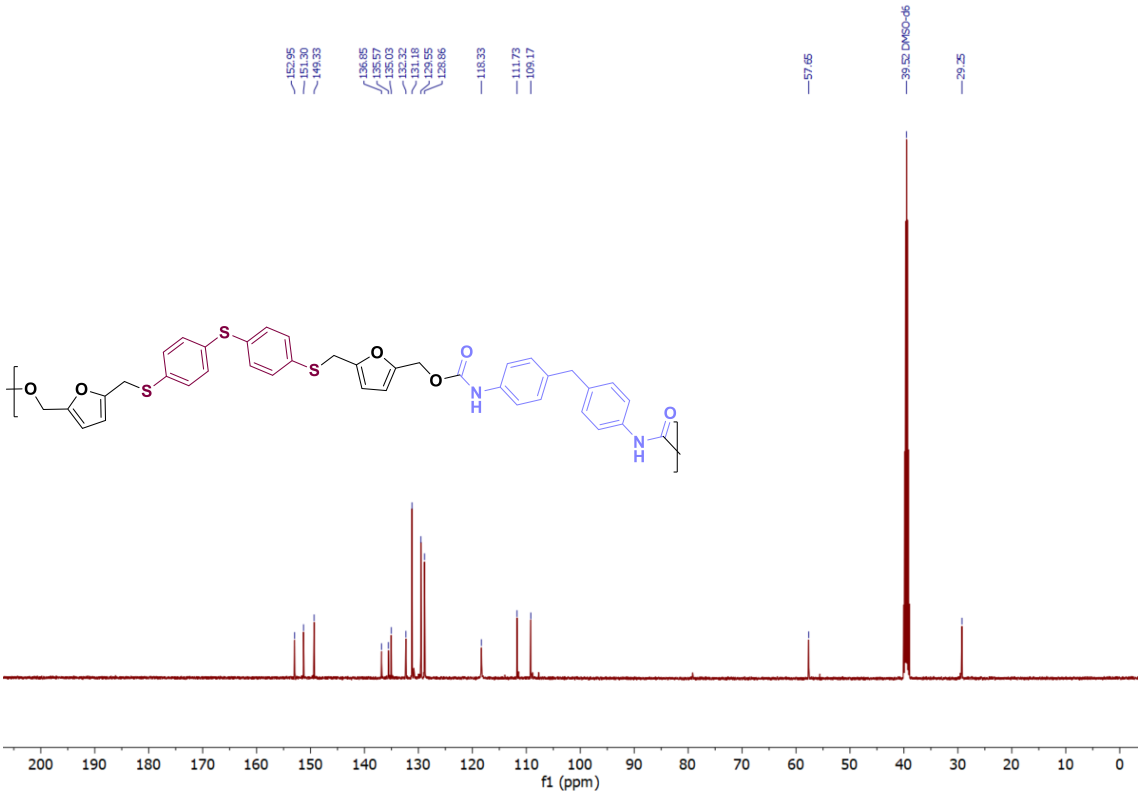

^{13}C NMR (101 MHz, DMSO-d_6) Polyurethane (5i) obtained using 0.5 h of reaction

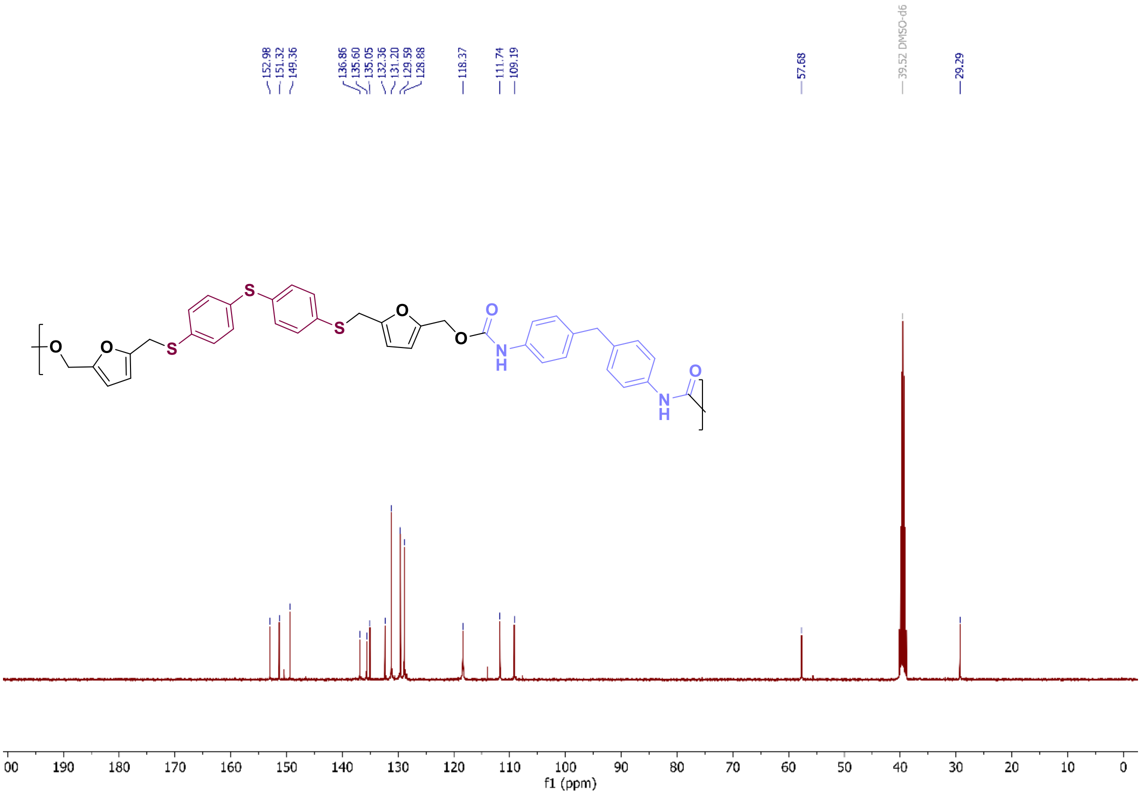

^{1}H NMR (500 MHz, DMSO-d_6) Polyurethane (5j) obtained using 18 h of reaction

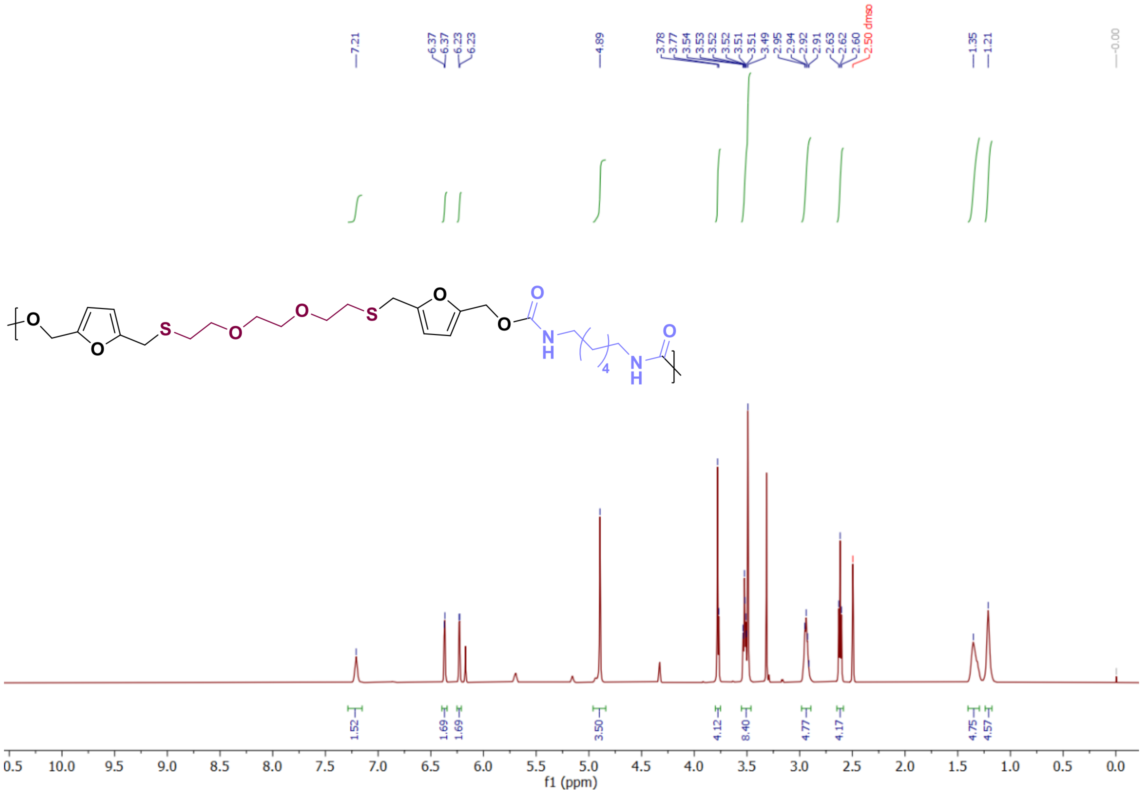

^{1}H NMR (400 MHz, DMSO-d_6) Polyurethane (5j) obtained using 0.5 h of reaction

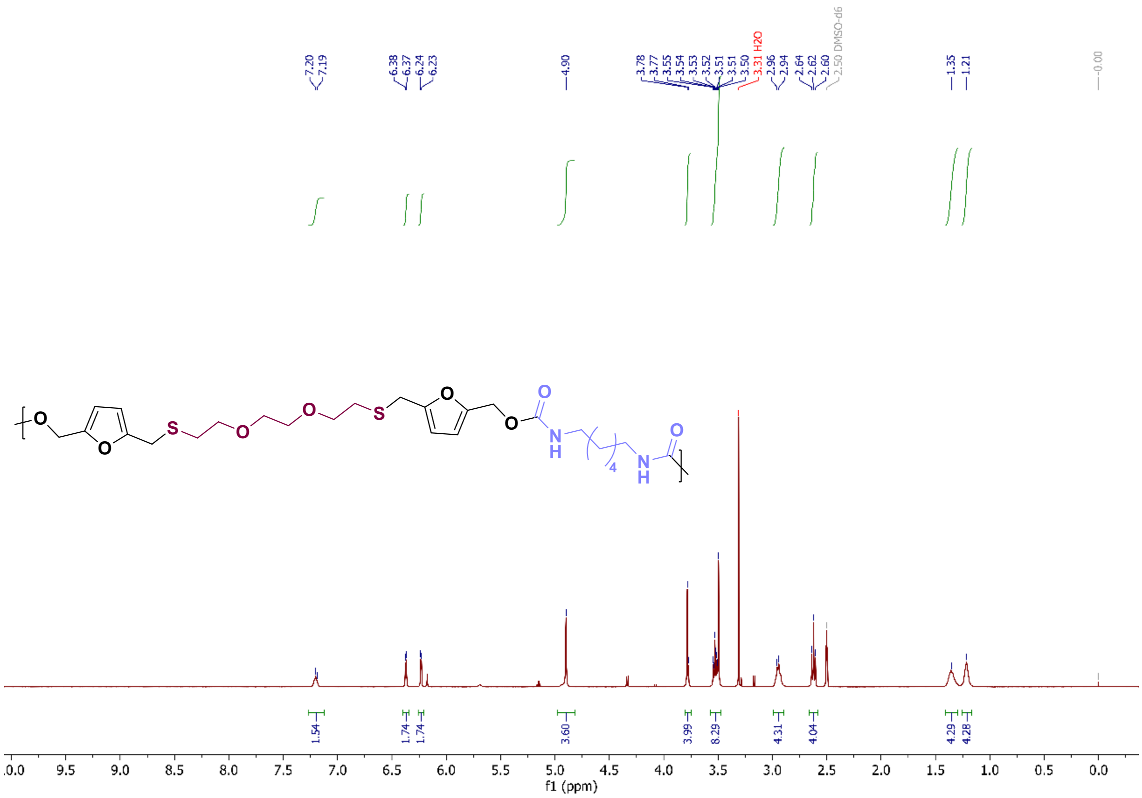

^{13}C NMR (126 MHz, DMSO-d$_6$) Polyurethane (5j) obtained using 18 h of reaction

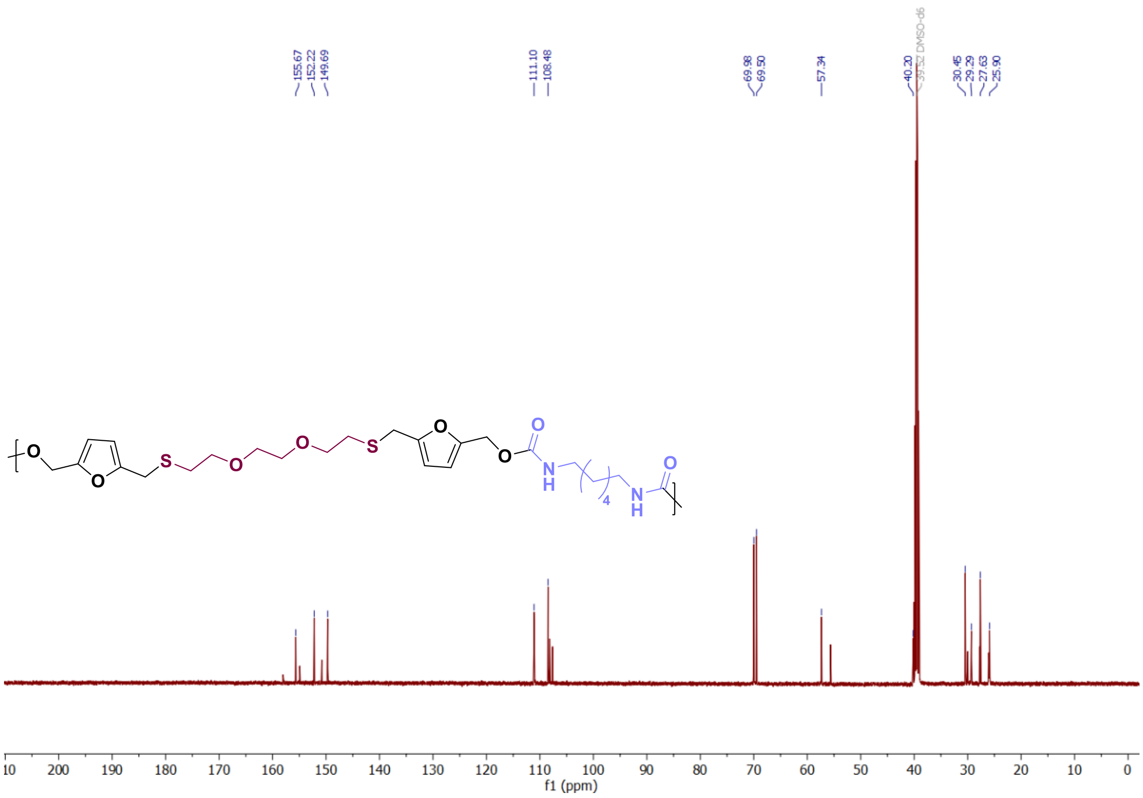

^{13}C NMR (101 MHz, DMSO-d$_6$) Polyurethane (5j) obtained using 0.5 h of reaction

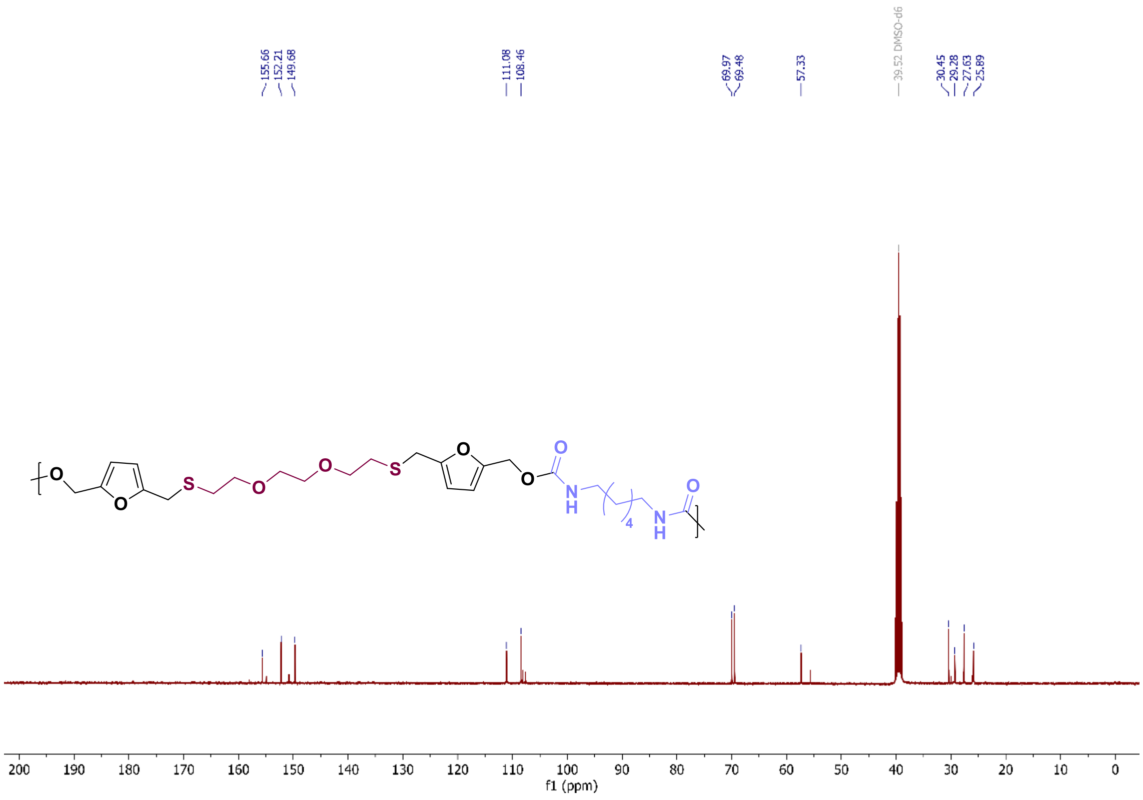

^{1}H NMR (500 MHz, DMSO-d_6) Polyurethane (5k) obtained using 18 h of reaction

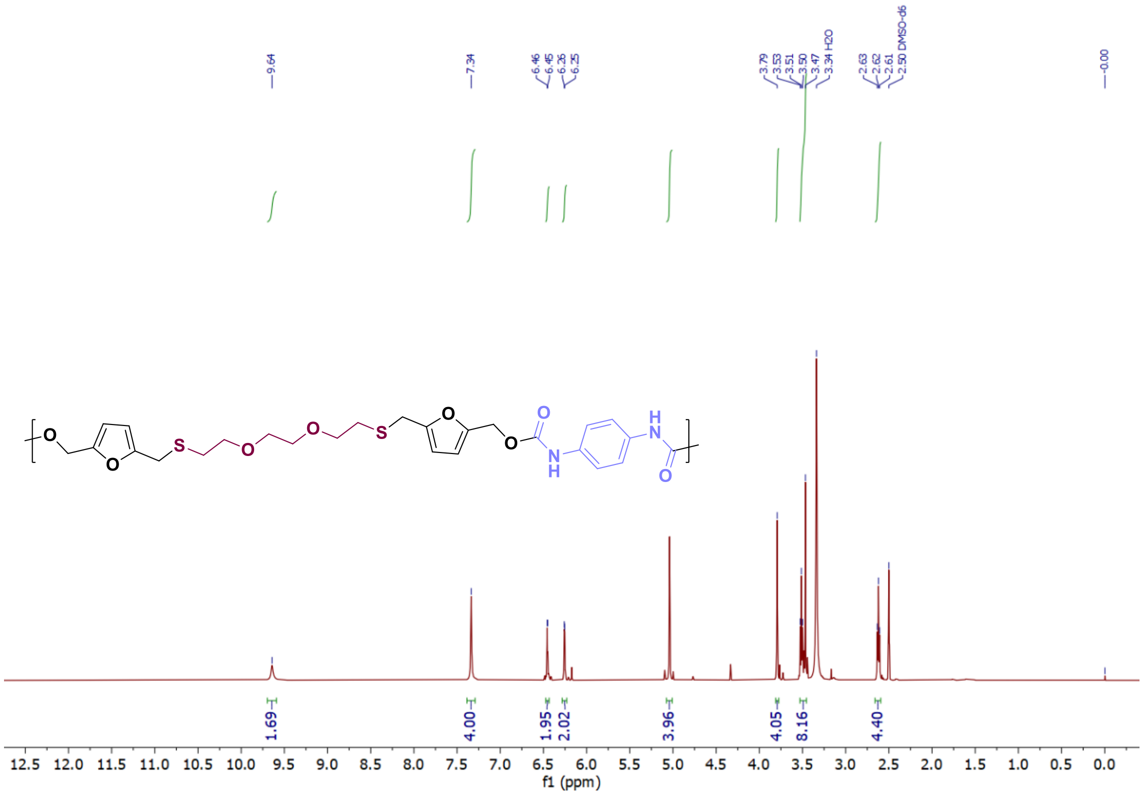

^{1}H NMR (400 MHz, DMSO-d_6) Polyurethane (5k) obtained using 0.5 h of reaction

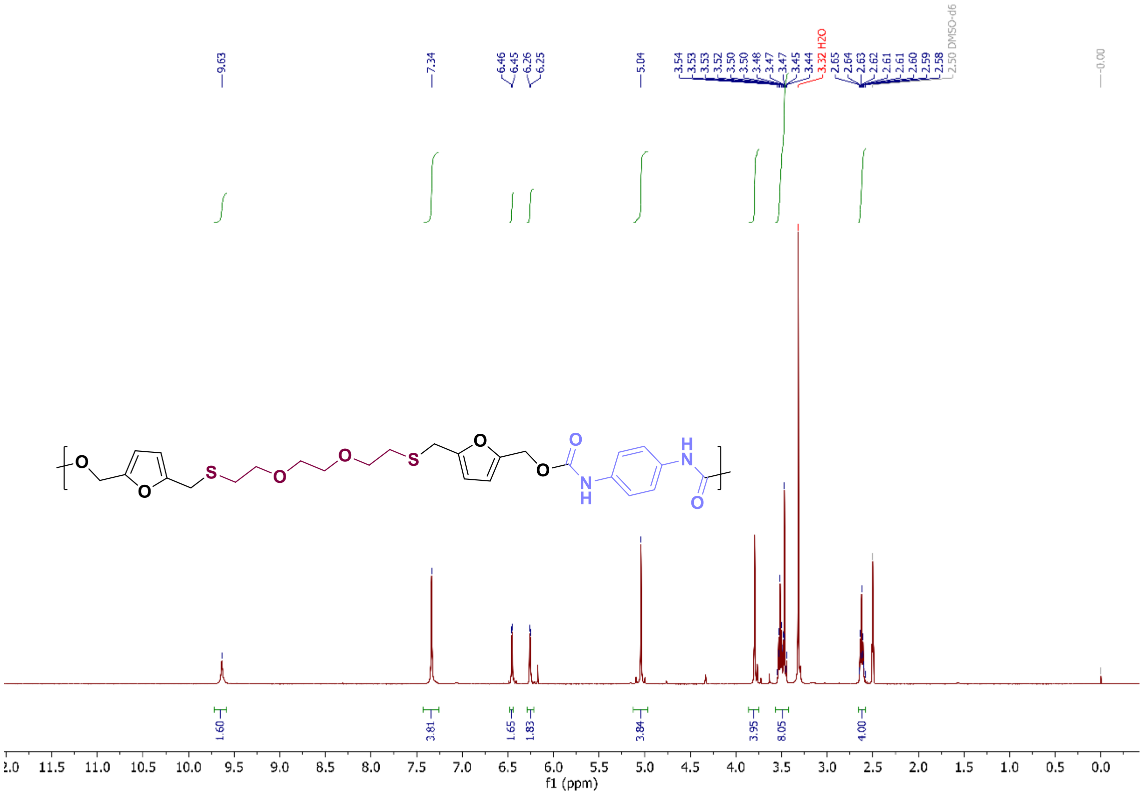

^{13}C NMR (126 MHz, DMSO-d_6) Polyurethane (5k) obtained using 18 h of reaction

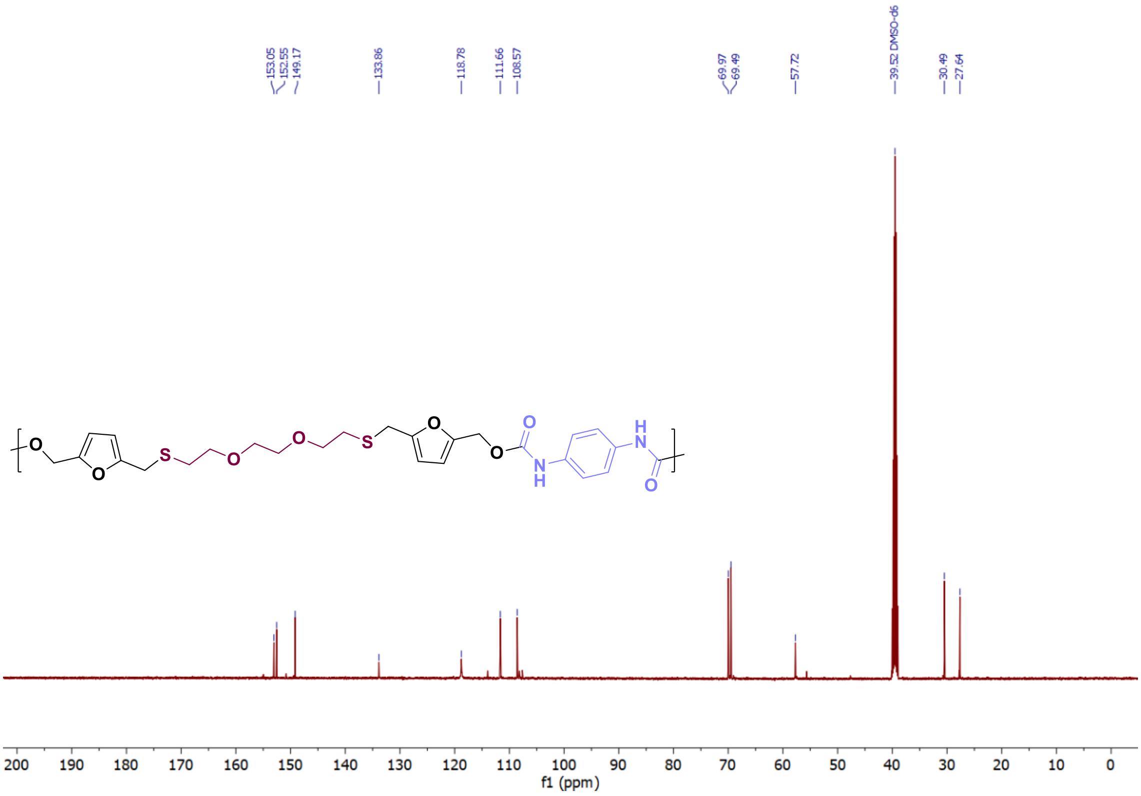

^{13}C NMR (101 MHz, DMSO-d_6) Polyurethane (5k) obtained using 0.5 h of reaction

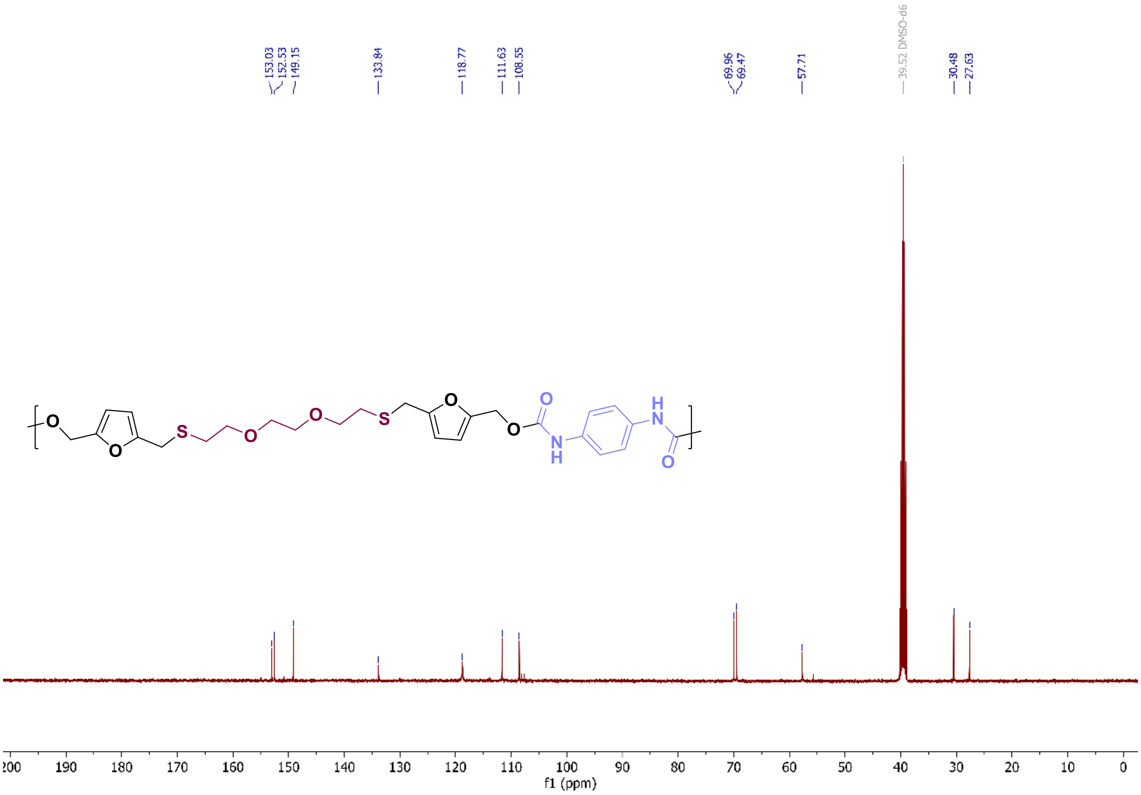

^1H NMR (500 MHz, DMSO-d$_6$) Polyurethane (5l) obtained using 18 h of reaction

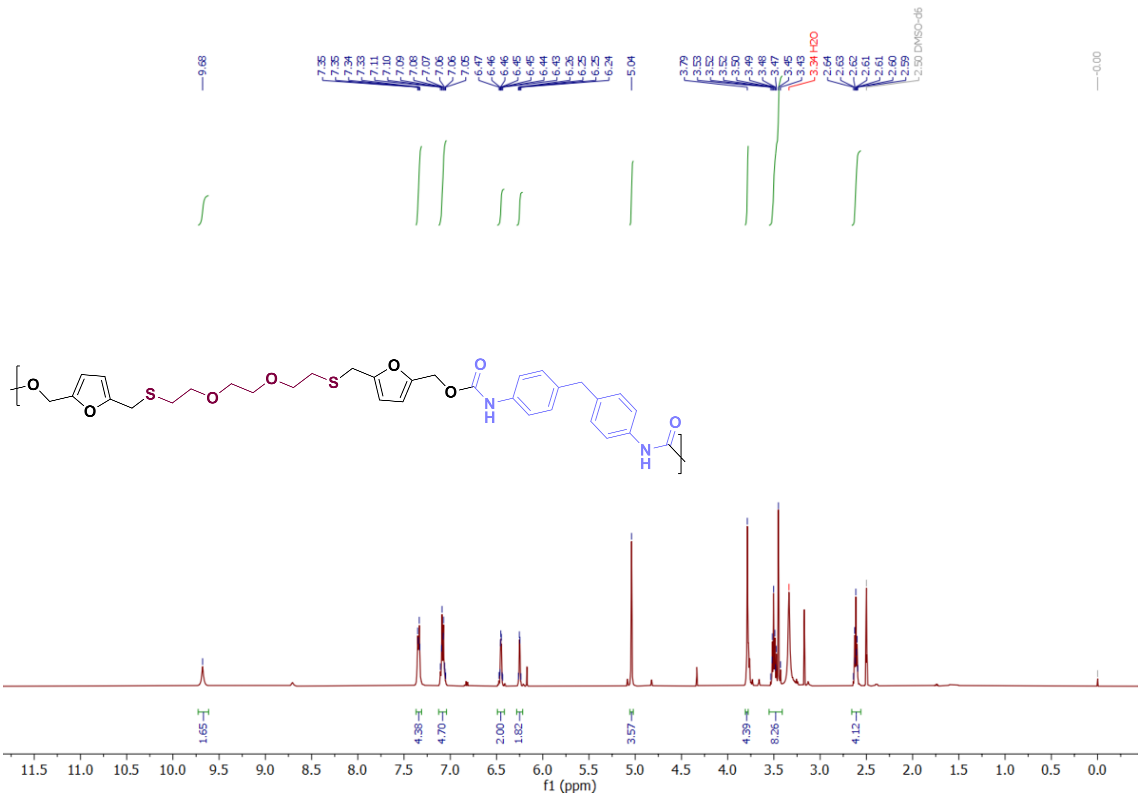

^1H NMR (400 MHz, DMSO-d$_6$) Polyurethane (5l) obtained using 0.5 h of reaction

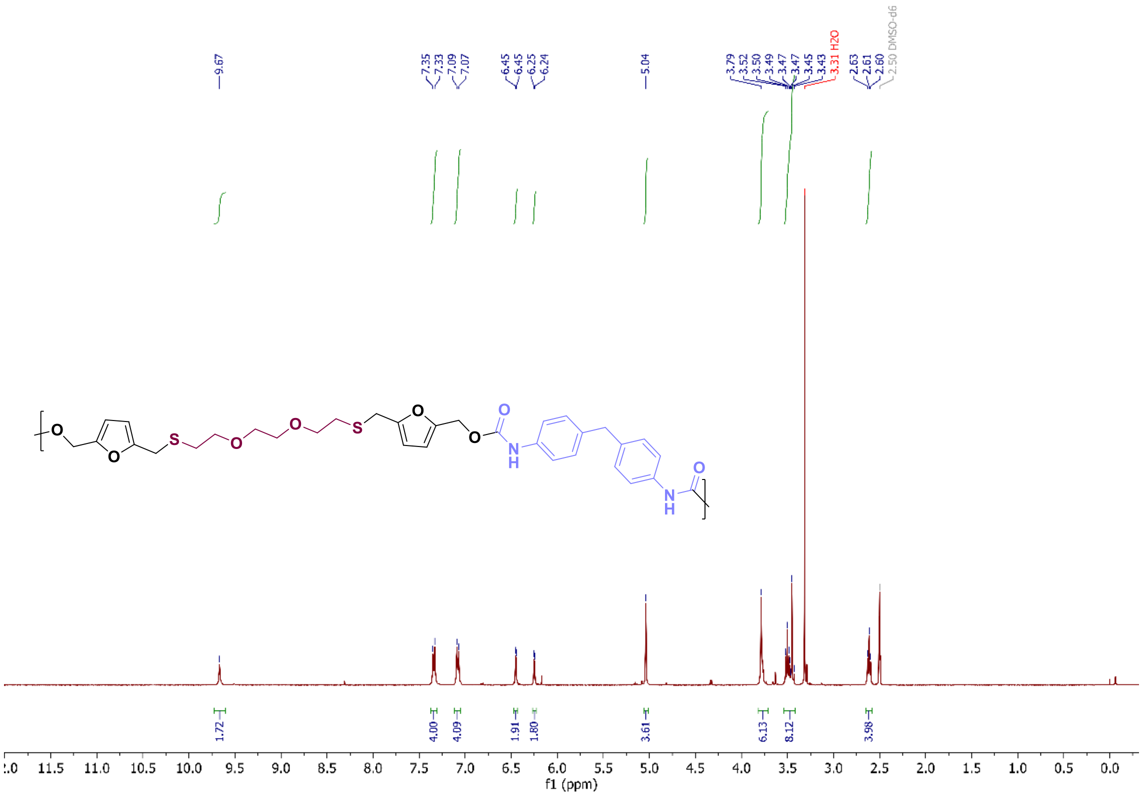

^{13}C NMR (126 MHz, DMSO-d$_6$) Polyurethane (5l) obtained using 18 h of reaction

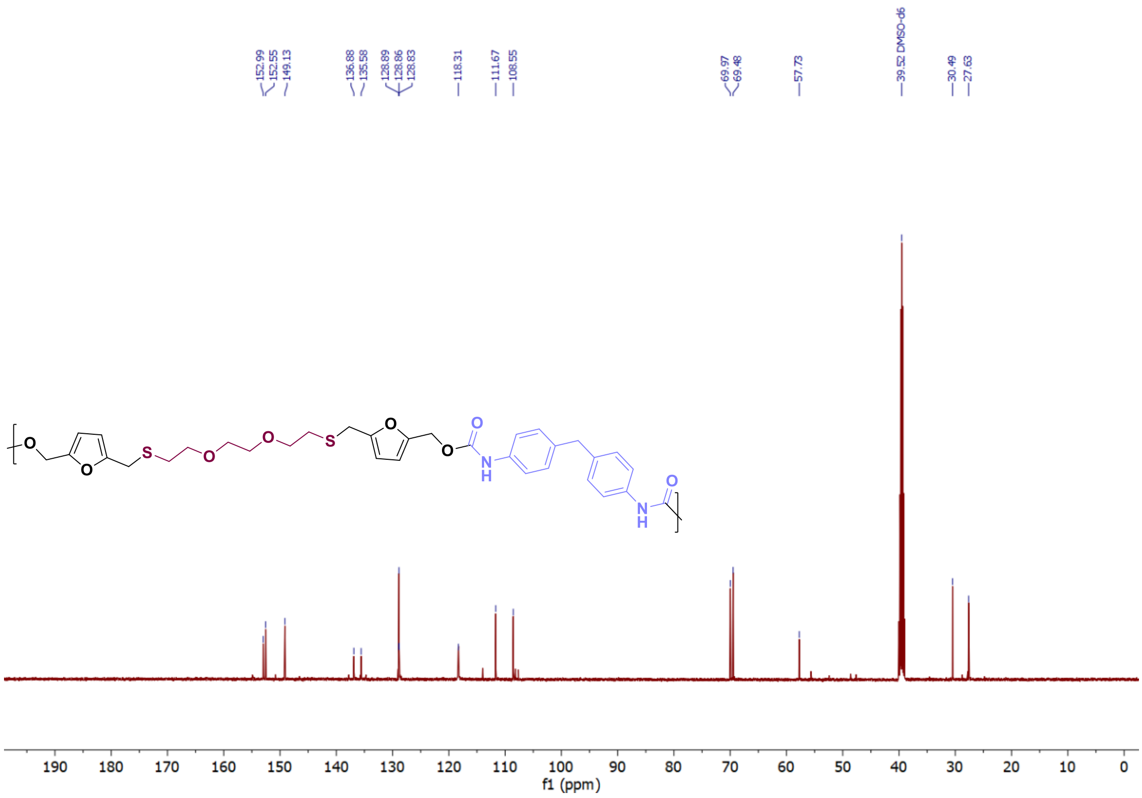

^{13}C NMR (101 MHz, DMSO-d$_6$) Polyurethane (5l) obtained using 18 h of reaction

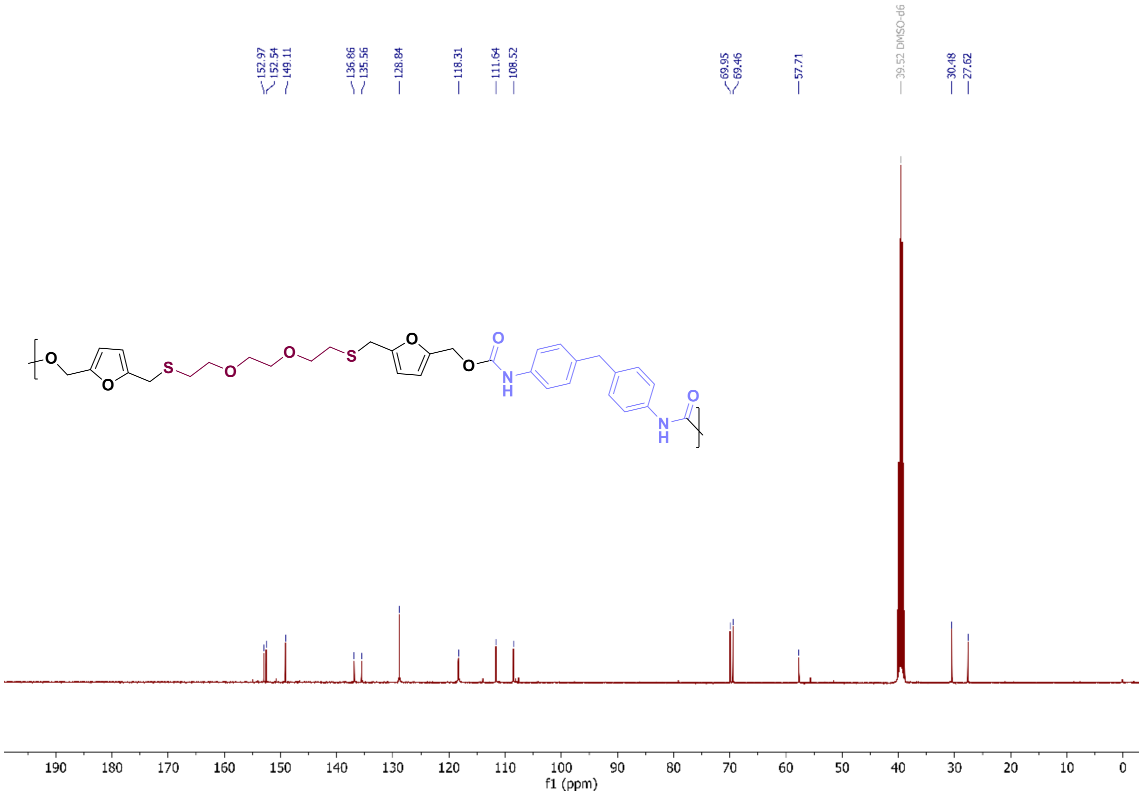

6. **Copies of the infrared spectra obtained for the polyurethanes (5).**

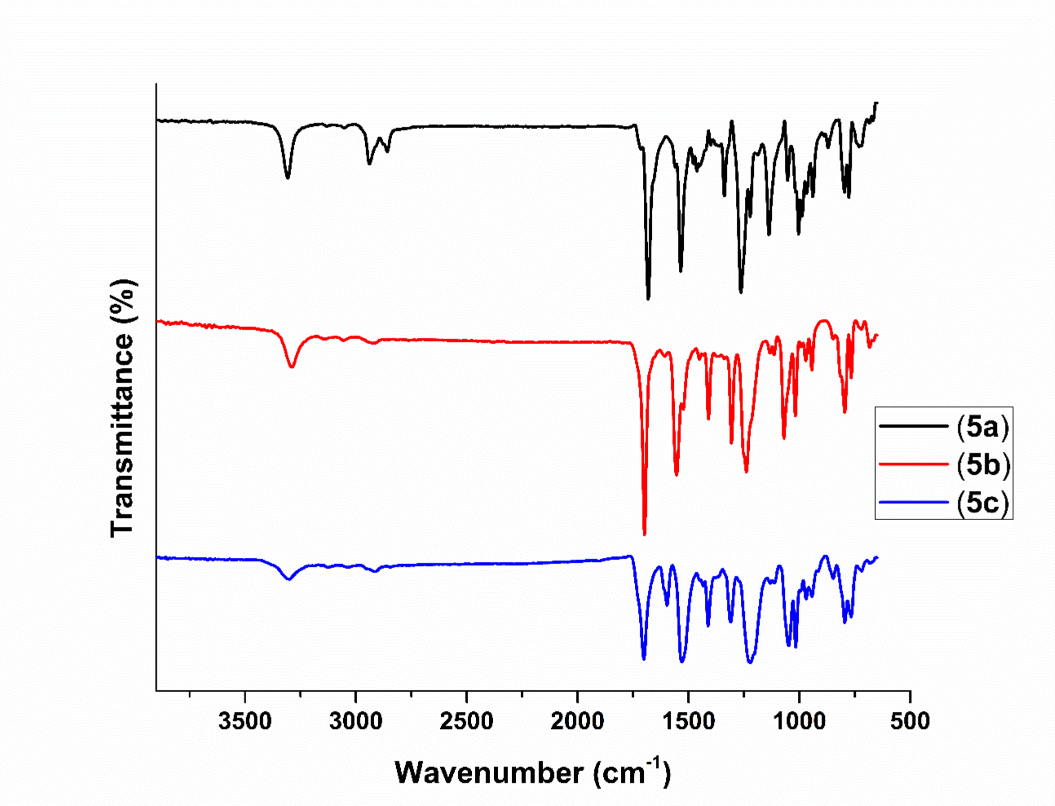

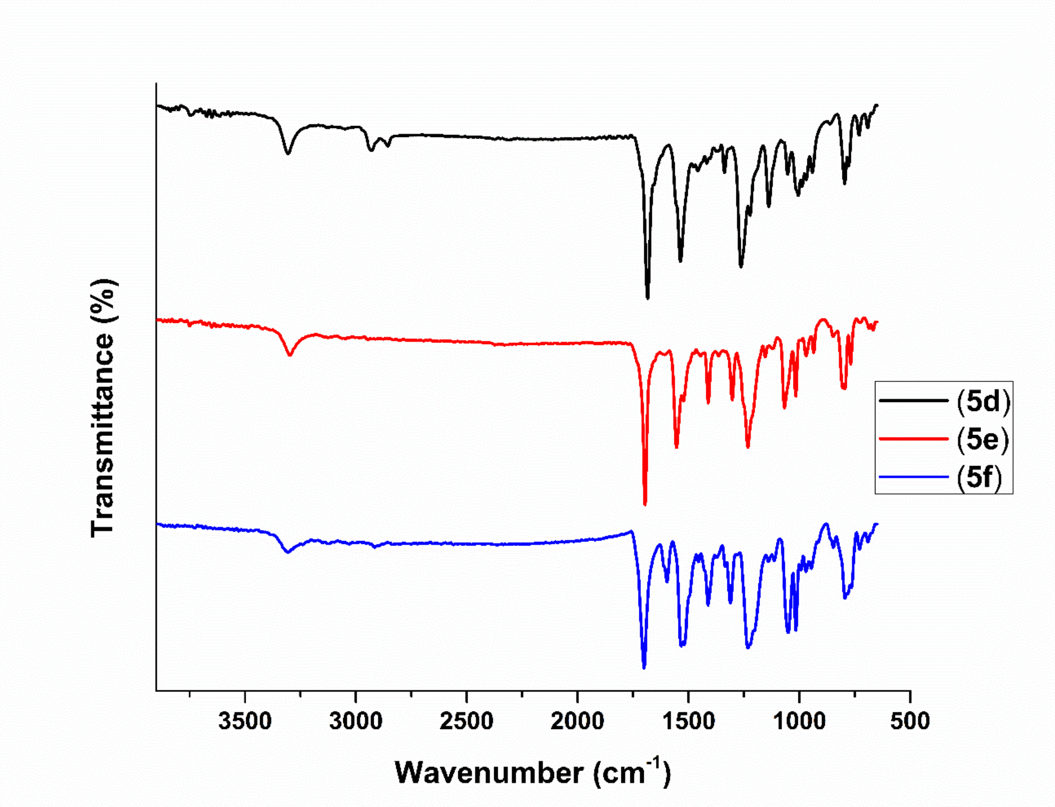

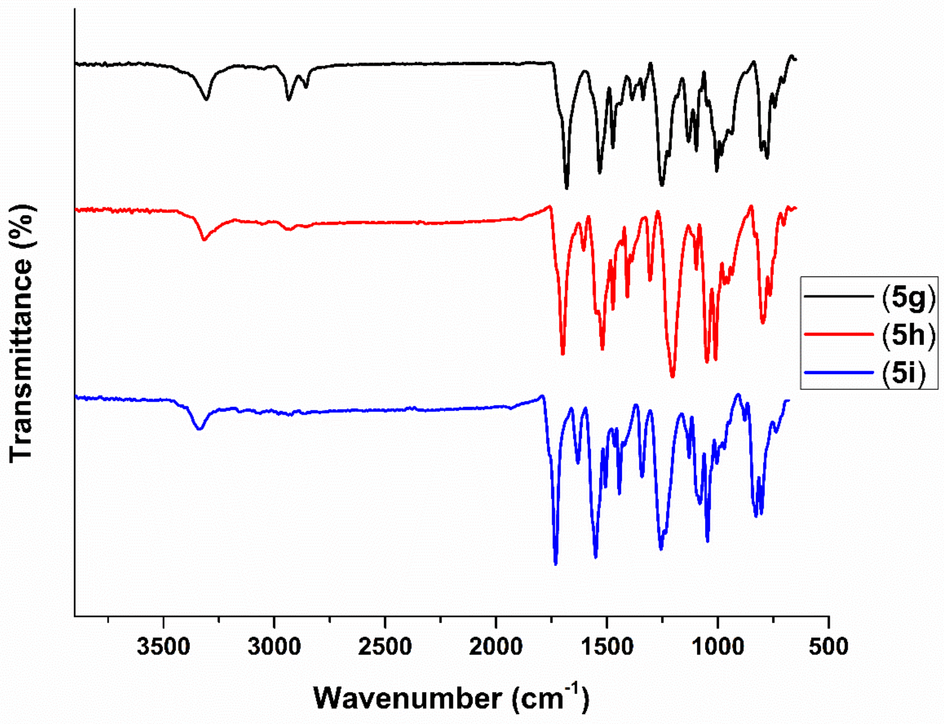
Transmittance (%)
3500
3000
2500
2000
1500
1000
500
Wavenumber (cm^{-1})
(5g)
(5h)
(5i)

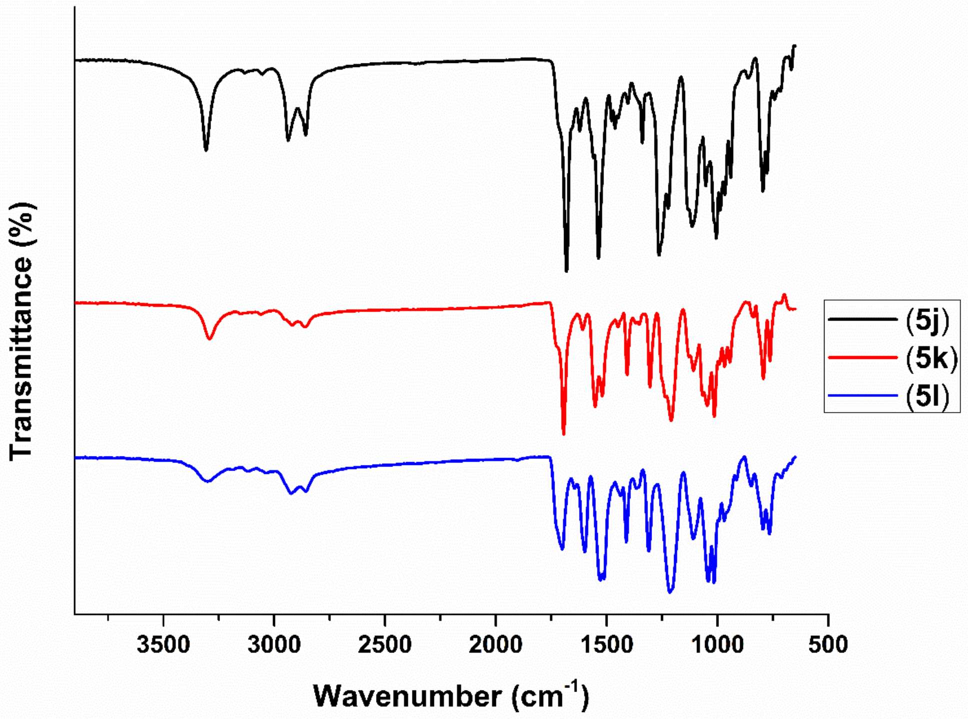
Transmittance (%)
3500
3000
2500
2000
1500
1000
500
Wavenumber (cm^{-1})
(5j)
(5k)
(5l)

7. Copies of the TGA thermograms and DTG curves obtained for the polyurethanes (5).

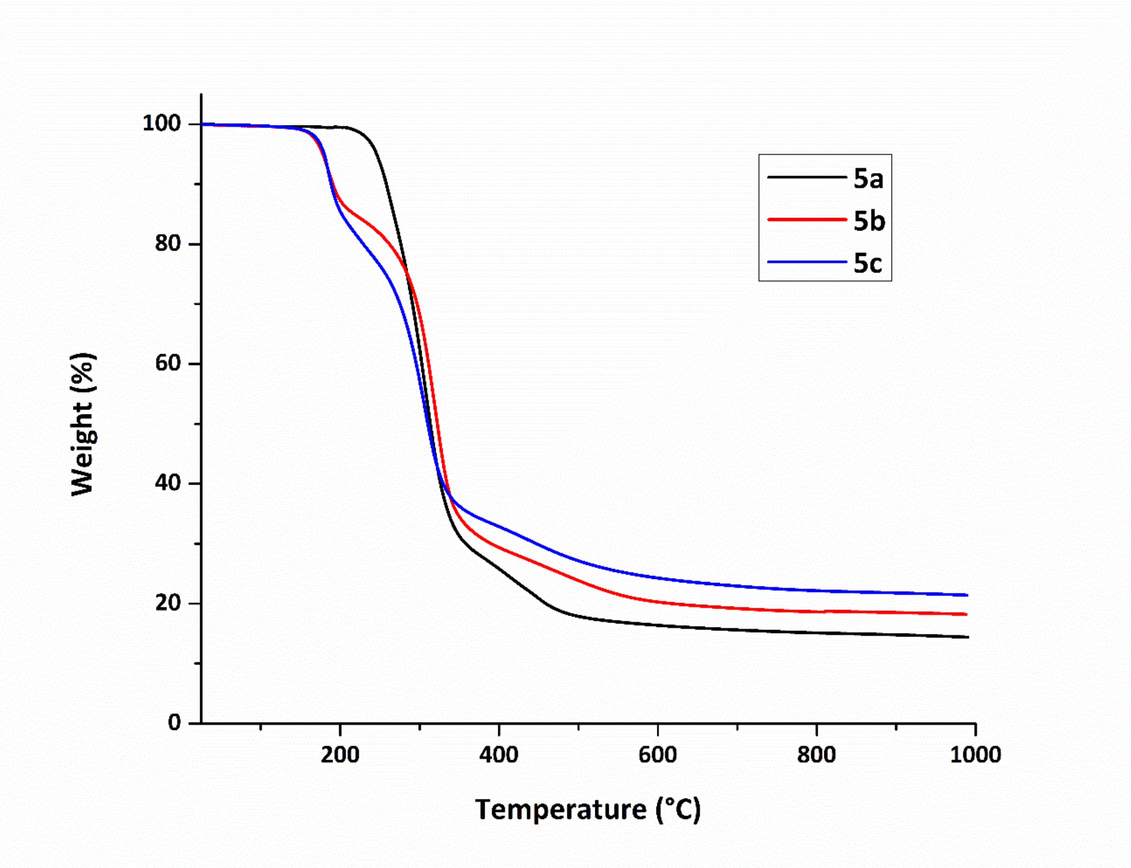

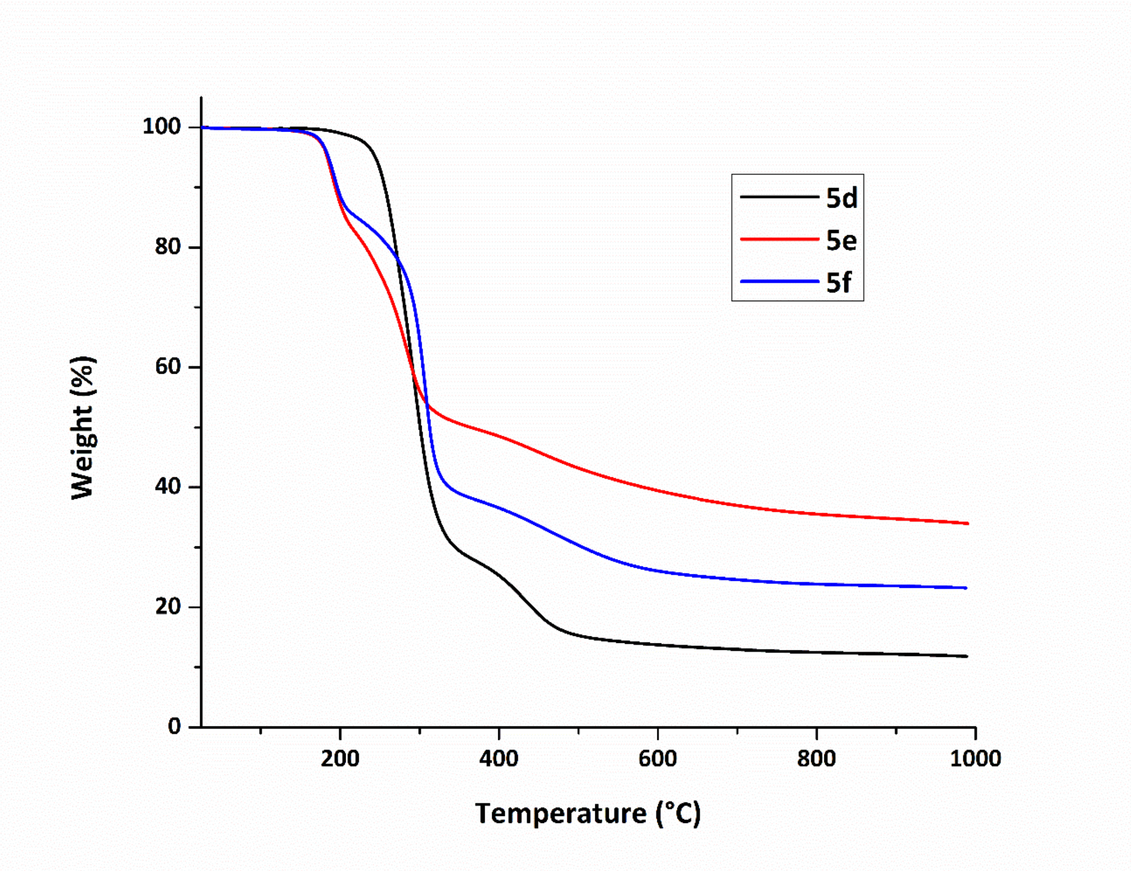

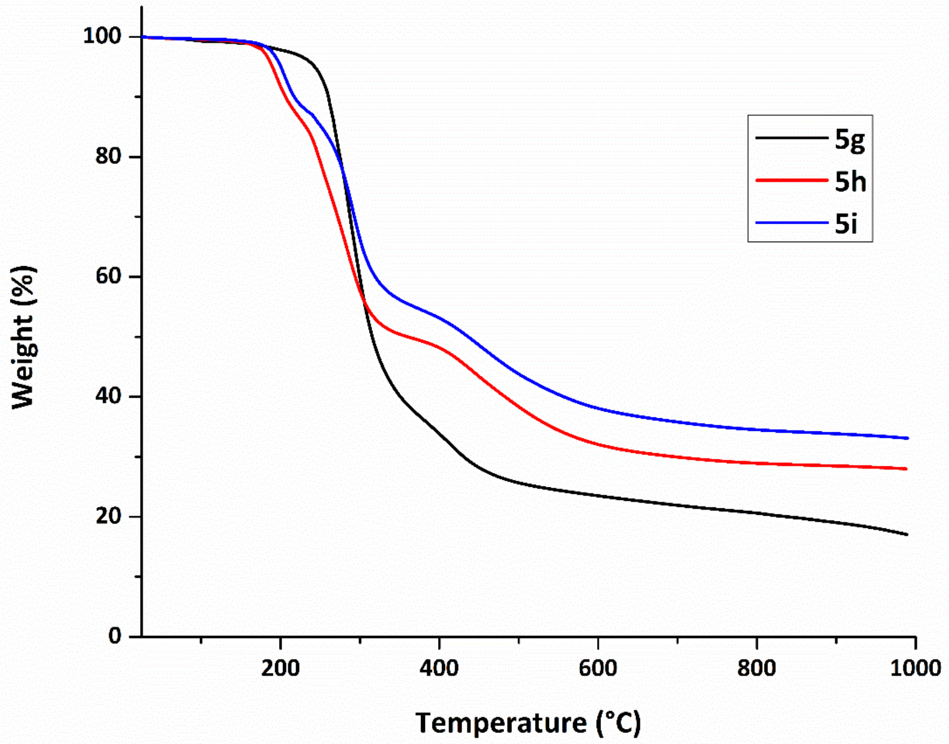
100
80
60
40
20
0
Weight (%)
200
400
600
800
1000
Temperature (°C)
5g
5h
5i

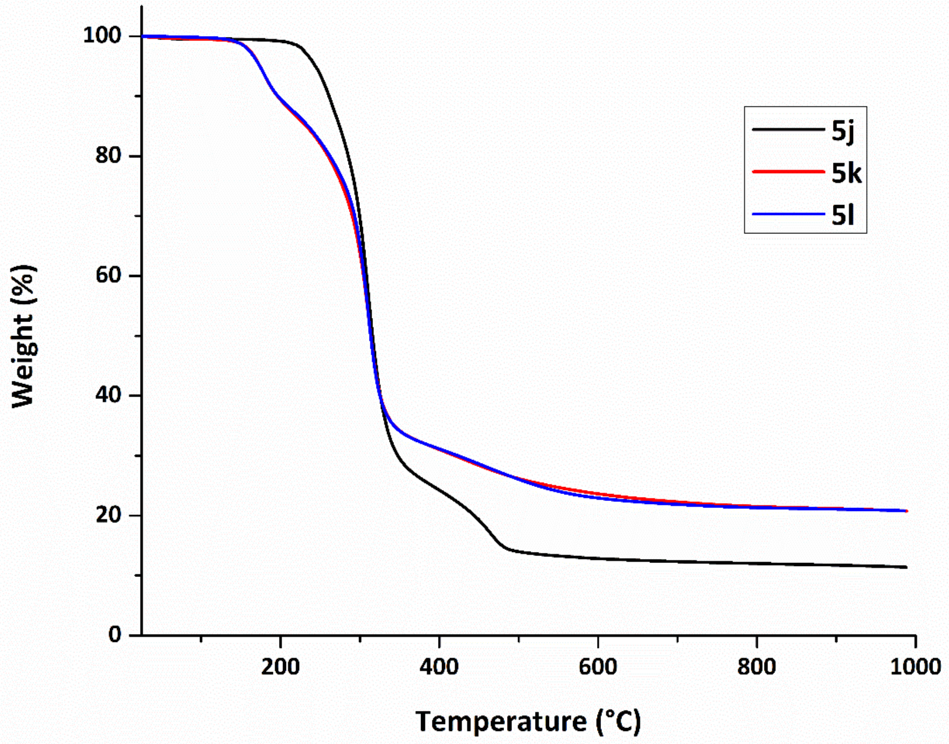
100
80
60
40
20
0
Weight (%)
200
400
600
800
1000
Temperature (°C)
5j
5k
5l

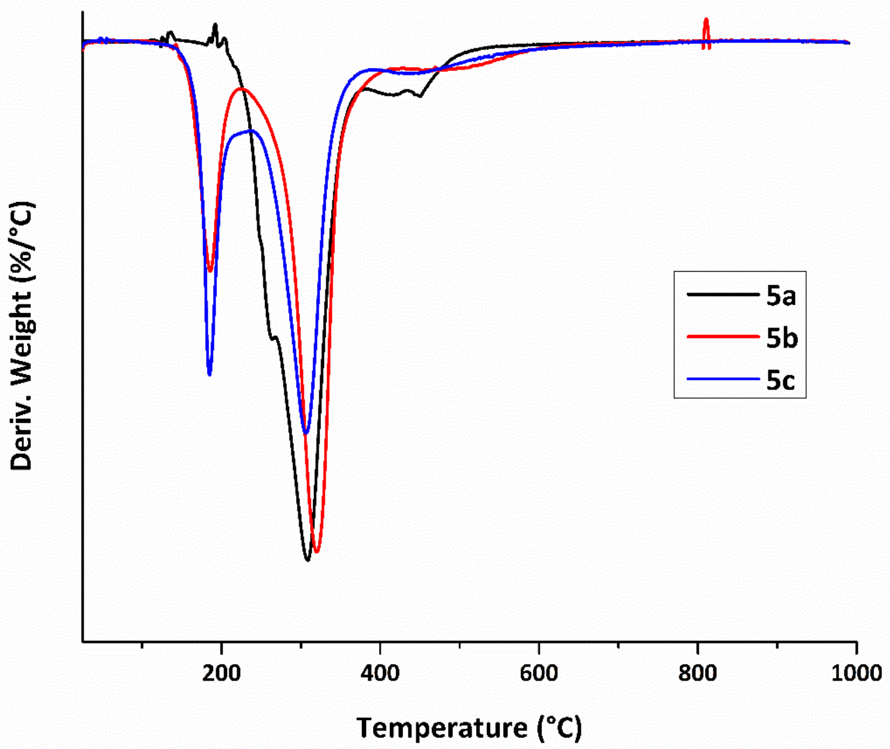

Deriv. Weight (%/°C)
5a
5b
5c
200
400
600
800
1000
Temperature (°C)

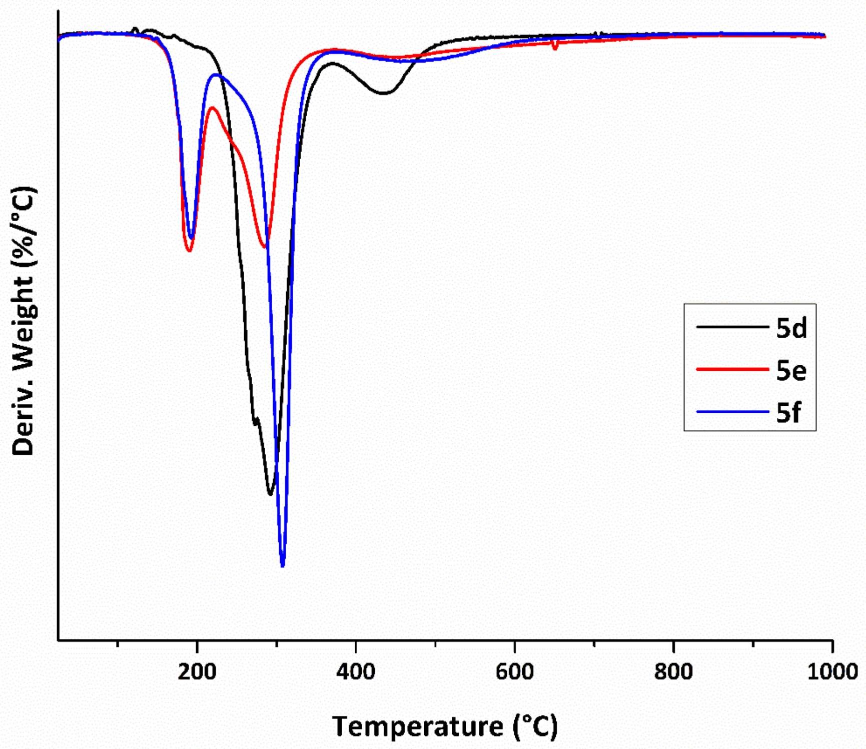

Deriv. Weight (%/°C)
5d
5e
5f
200
400
600
800
1000
Temperature (°C)

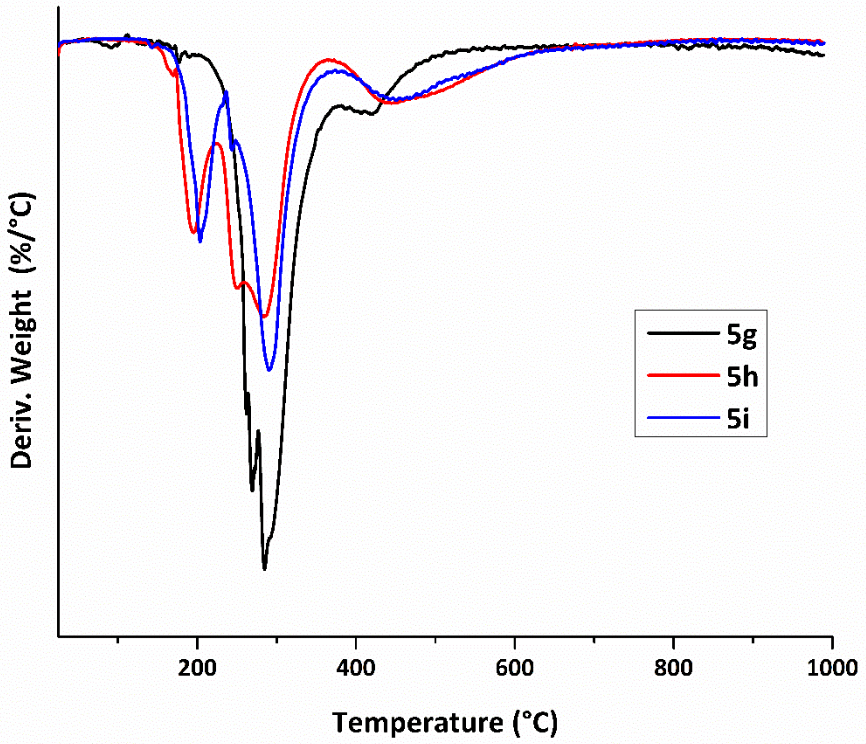
Deriv. Weight (%/°C)
200
400
600
800
1000
Temperature (°C)
5g
5h
5i

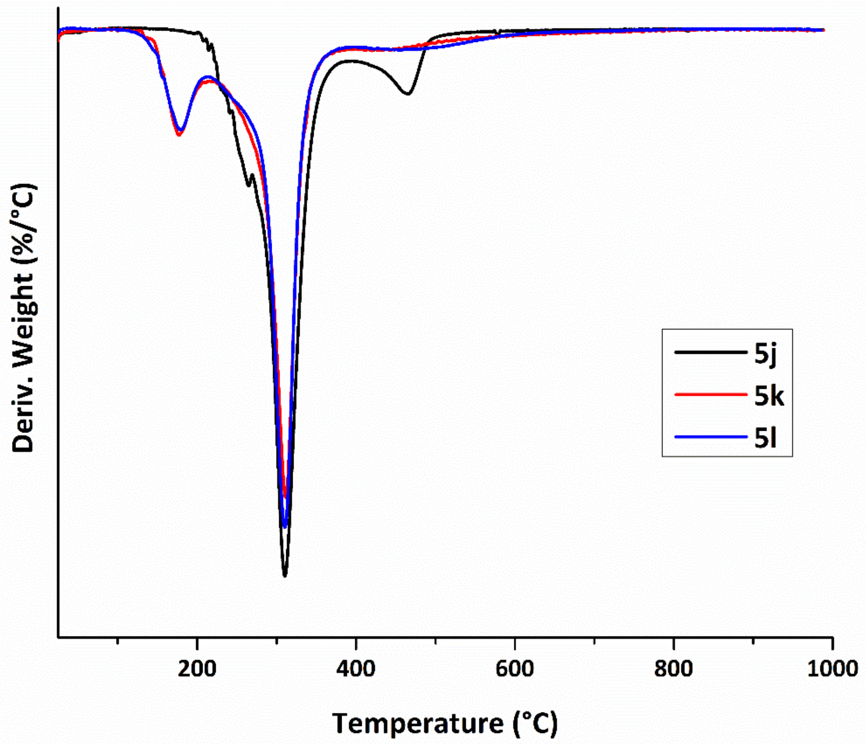
Deriv. Weight (%/°C)
200
400
600
800
1000
Temperature (°C)
5j
5k
5l

8. **Copies of the Debye plots obtained for each PU synthesized using 18 h of reaction.**

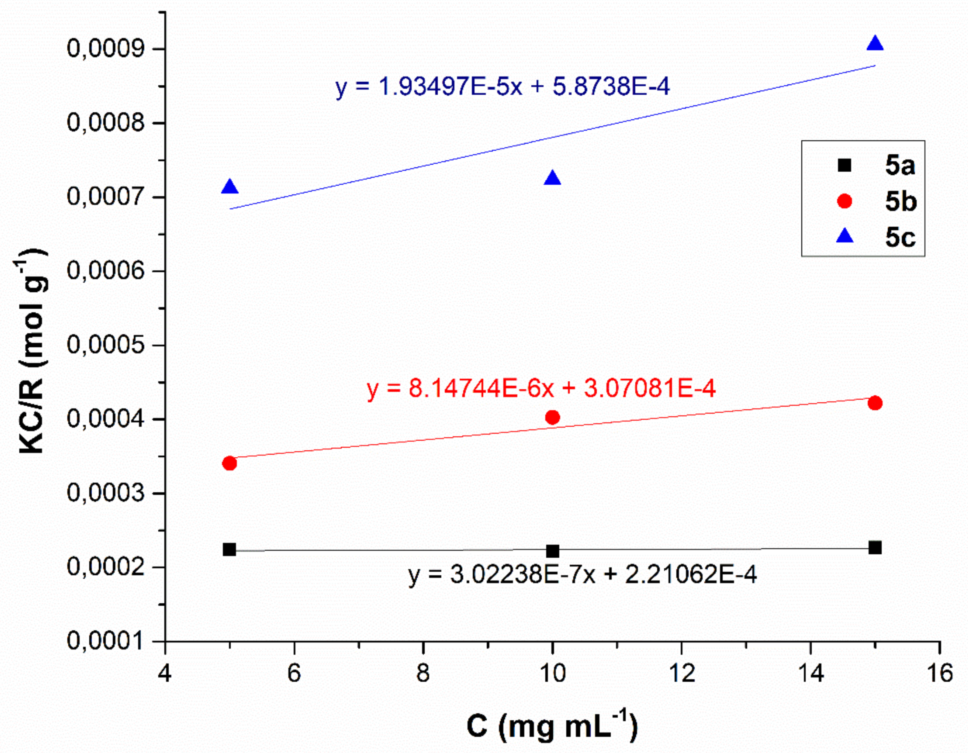

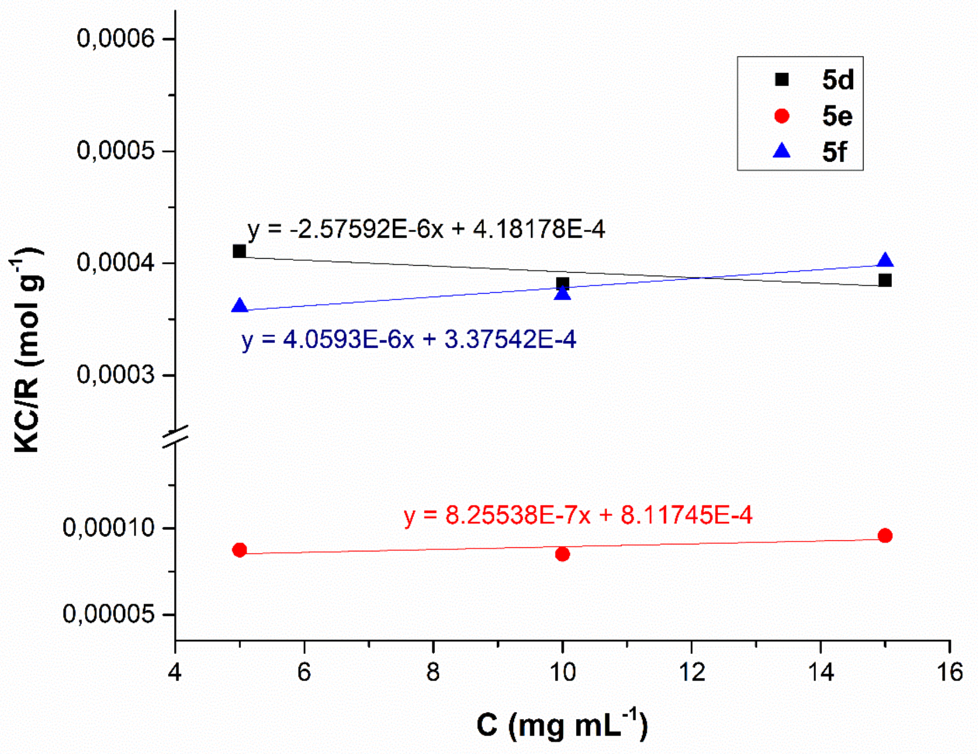

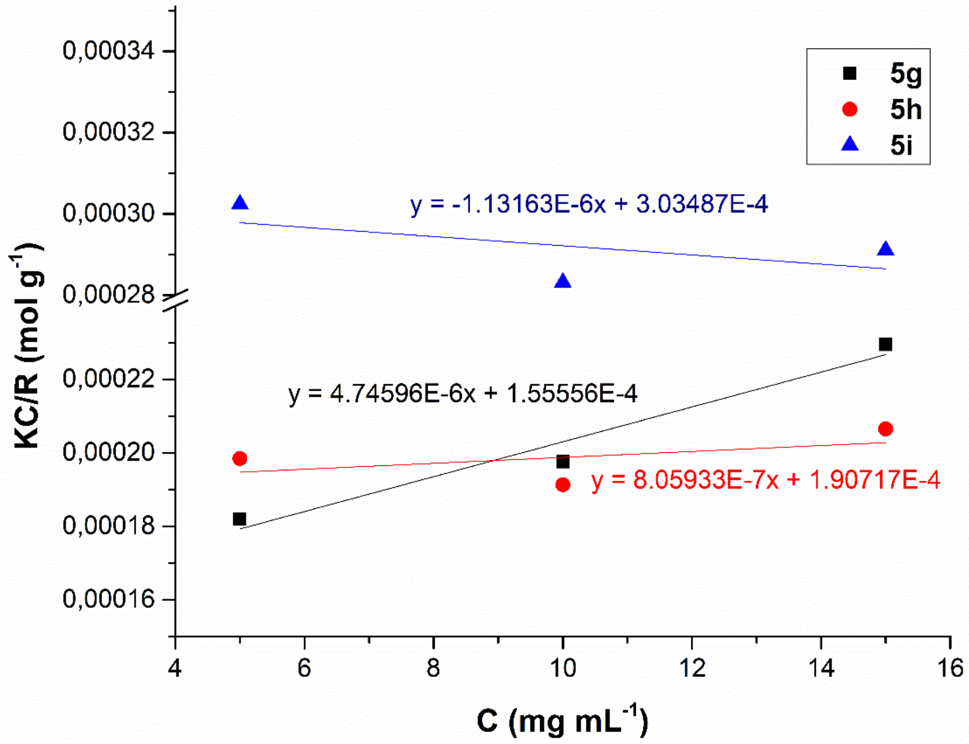
5g
5h
5i
y = -1.13163E-6x + 3.03487E-4
y = 4.74596E-6x + 1.55556E-4
y = 8.05933E-7x + 1.90717E-4
KC/R (mol g-1)
C (mg mL-1)

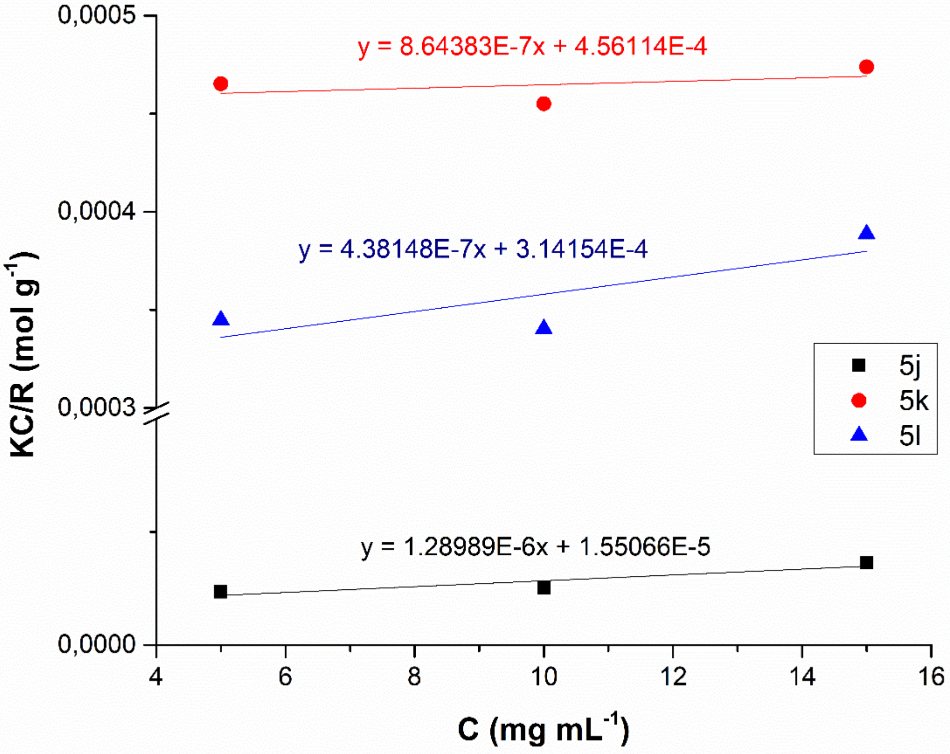
y = 8.64383E-7x + 4.56114E-4
y = 4.38148E-7x + 3.14154E-4
5j
5k
5l
y = 1.28989E-6x + 1.55066E-5
KC/R (mol g-1)
C (mg mL-1)

9. **Copies of the Debye plots obtained for each PU synthesized using 0.5 h of reaction.**

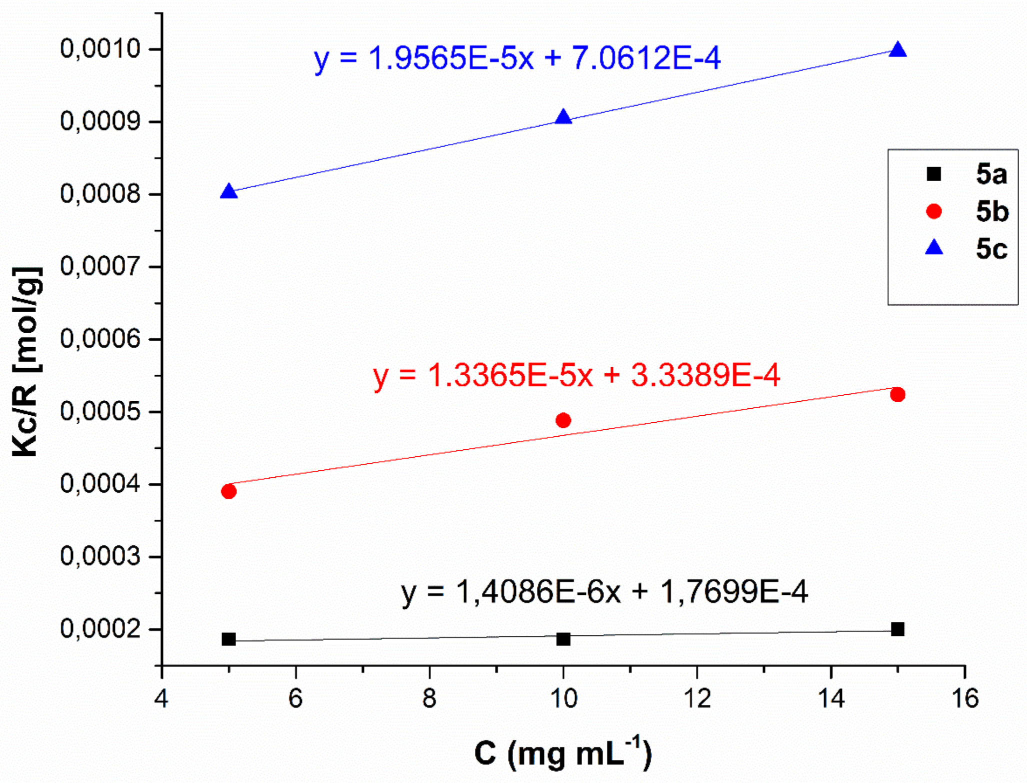

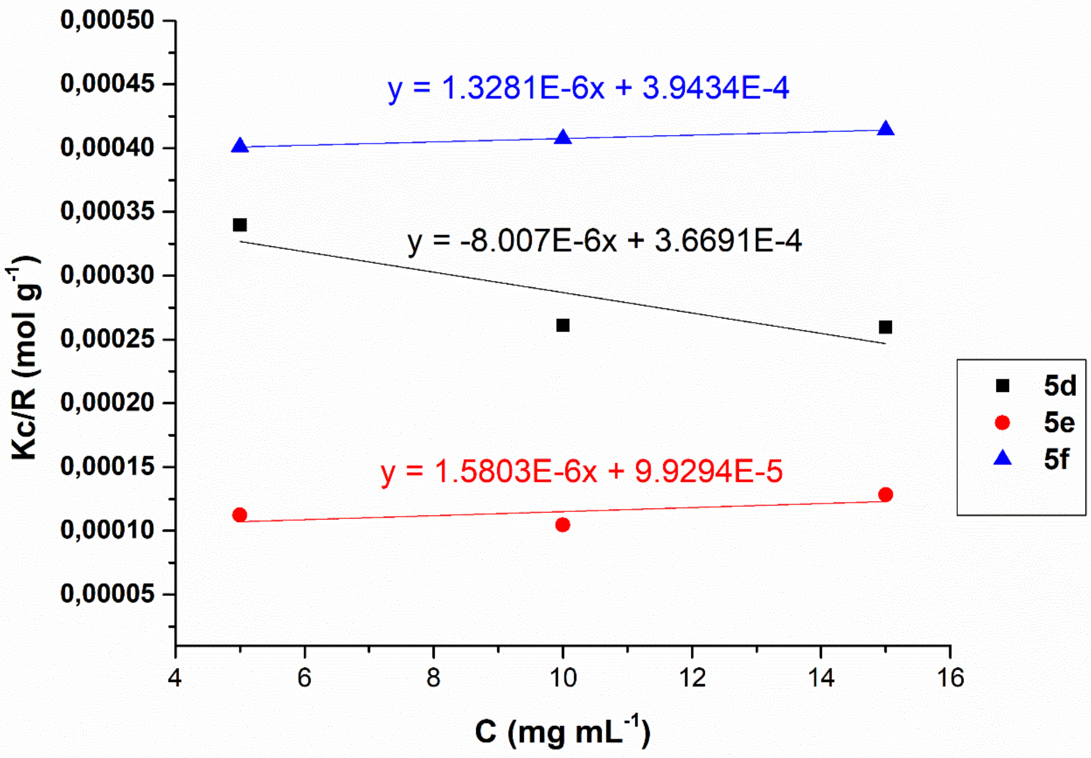

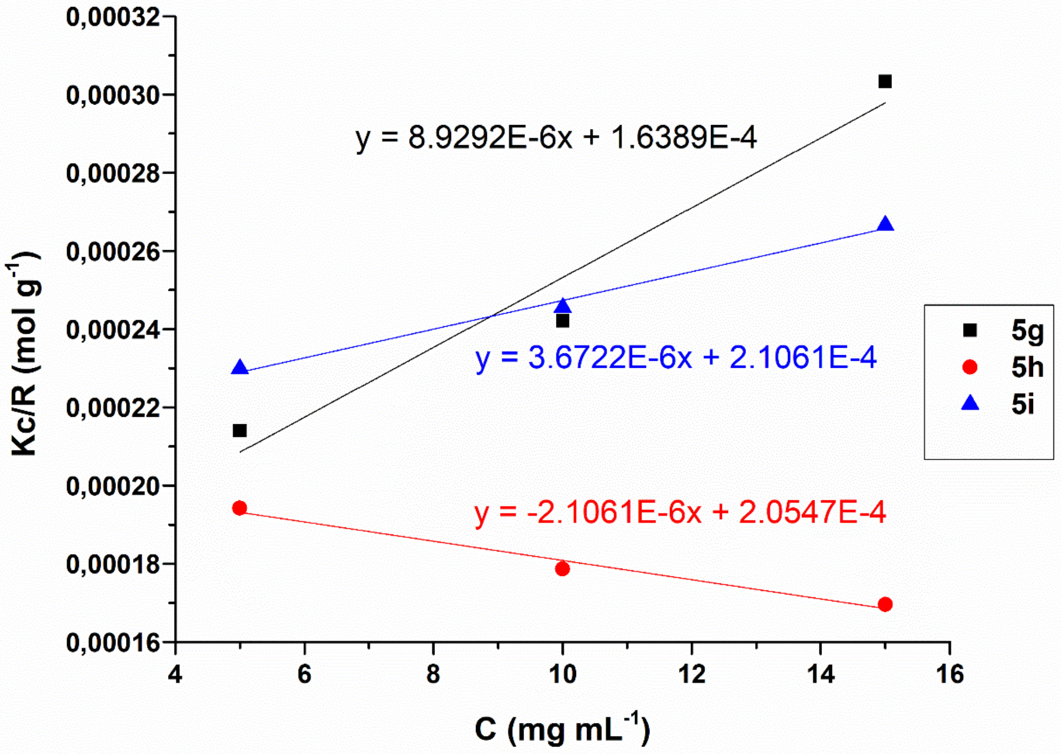

0,00032
0,00030
0,00028
0,00026
0,00024
0,00022
0,00020
0,00018
0,00016
4
6
8
10
12
14
16
C (mg mL-1)
Kc/R (mol g-1)
y = 8.9292E-6x + 1.6389E-4
y = 3.6722E-6x + 2.1061E-4
y = -2.1061E-6x + 2.0547E-4
5g
5h
5i

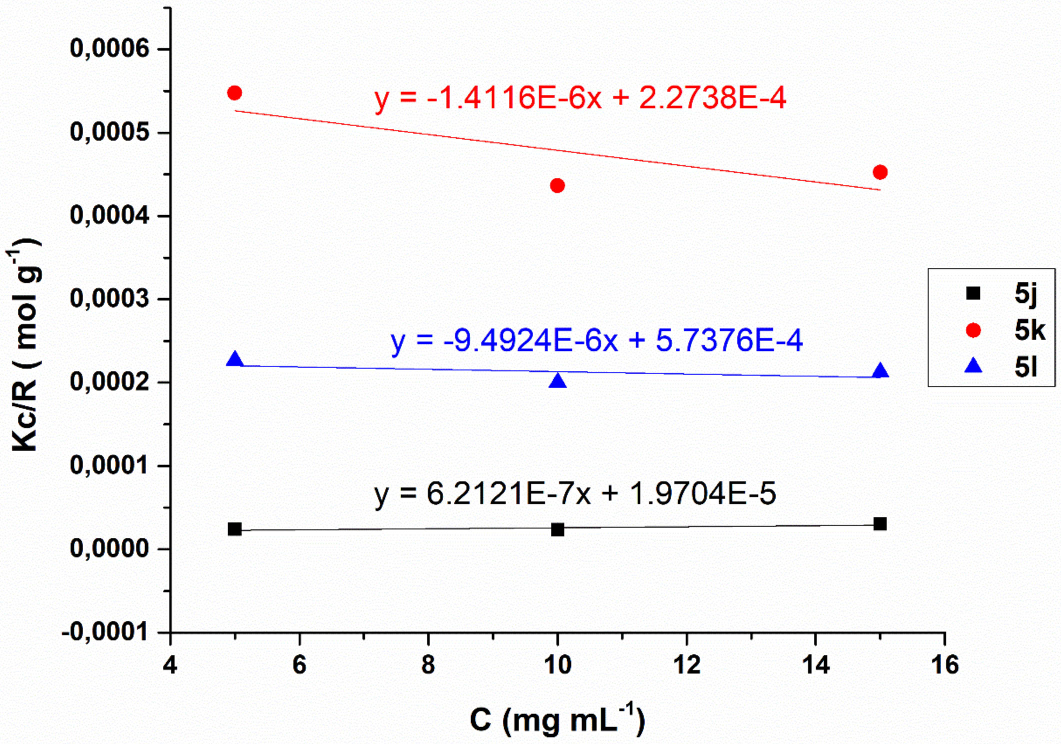

0,0006
0,0005
0,0004
0,0003
0,0002
0,0001
0,0000
-0,0001
4
6
8
10
12
14
16
C (mg mL-1)
Kc/R (mol g-1)
y = -1.4116E-6x + 2.2738E-4
y = -9.4924E-6x + 5.7376E-4
y = 6.2121E-7x + 1.9704E-5
5j
5k
5l

Table S1. Molecular weights obtained for the polymer synthesized at different reaction times.[a]

Polyurethane	Molecular weights (kDa)		2nd virial coefficient (mol*ml/g^2)	
	18 h	0.5 h	18 h	0.5 h
**5a**	4.5	5.6	-1.4E-04	7.0E-04
**5b**	2.2	3.0	4.3E-04	6.7E-03
**5c**	1.7	1.4	9.7E-03	9.8E-03
**5d**	2.6	2.7	5.2E-04	-4.0E-03
**5e**	12.3	10.1	4.1E-04	7.9E-04
**5f**	2.8	2.5	1.2E-03	6.6E-04
**5g**	6.4	6.1	2.4E-03	4.5E-03
**5h**	5.2	4.9	4.0E-04	-1.2E-03
**5i**	3.3	4.7	2.2E-03	1.8E-03
**5j**	57.7	50.7	6.3E-04	3.1E-04
**5k**	2.2	1.7	4.3E-04	-4.7E-04
**5l**	3.0	4.4	2.0E-03	-7.1E-04

[a] Determined using static light scattering (SLS).

10. Synthesis of the polymer 5m via Curtius rearrangement

A 50 mL round bottom flask with a distillation column was charged with 2.0 g of 5-(chloromethyl)furfural (CMF, 13.8 mmol) and 14 mL of nitric acid (HNO_3, 67%v/v), and the formed mixture reacted for 30 min at room temperature. Then, the temperature was increased to 40°C and the mixture reacted for 24 h. After completion, the liquid was evaporated under reduced pressure, and the solid obtained was filtered, washed with water, and dried in a high vacuum to afford 1.44 g of the furan dicarboxylic acid (67% yield), which was employed in the next step without further purifications. In the next step, 1.0 g of the obtained solid was dissolved in dry THF (15 mL) under an argon atmosphere. Then, triethylamine (2.9 eq.) was added to the reaction mixture. Subsequently, diphenyl phosphoryl azide (2.9 eq.) was added dropwise, and the mixture reacted for 18 h at room temperature. Upon completion, the solvent was evaporated under reduced pressure, and the obtained crude material was purified using a chromatography column loaded with silica gel pre-eluted with the mobile phase solvents (EtOAc/Hex 1:9). Chromatography afforded the desired furan-2,5-diacyl azide as a white solid (0.9 g, 70%). Furan-2,5-diacyl azide (FDAz) = **^{1}H NMR (400 MHz, $CDCl_3$)** δ 7.29 (s, 2H). **^{13}C NMR (101 MHz, $CDCl_3$)** δ 162.4, 148.5, 120.1.

Then, 0.5 mmol of FDAz and 3 mL of dry THF were added under an argon atmosphere in a 10 mL flask. Subsequently. a solution containing 0.5 mmol of the corresponding diol **4a** and 20 mol% of DBU in 2 mL of dry THF was added. The mixture formed reacted for 18 h at reflux. Then, the solvent was evaporated under reduced pressure, and the polymer was precipitated with methanol.

Polyurethane (**5m**) = The product was obtained as a brown light solid after precipitation with methanol (208 mg, 78%). **^{1}H NMR (500 MHz, DMSO-d_6)** δ 7.53 (s, 2H), 6.54 (d, J = 3.2 Hz, 2H), 6.27 (d, J = 3.2 Hz, 2H), 5.28 (s, 4H), 3.75 (s, 4H), 2.54 (t, J = 7.1 Hz, 4H), 1.63 (p, J = 7.1 Hz, 2H). **^{13}C NMR (126 MHz, DMSO-d_6)** δ 157.1, 154.1, 147.6, 145.8, 120.1, 112.9, 108.1, 58.8, 30.2, 28.4, 27.4. **IR: ν (neat, ATR)/cm^{-1}** 3126, 2915, 1719, 1581, 1554, 1438, 1368, 1298, 1272, 1225, 1149,1125, 1021, 975, 943, 797, 766.

Copies of the RMN spectral

^{1}H NMR (400 MHz, $CDCl_3$) of Furan-2,5-diacyl azide (FDAz)

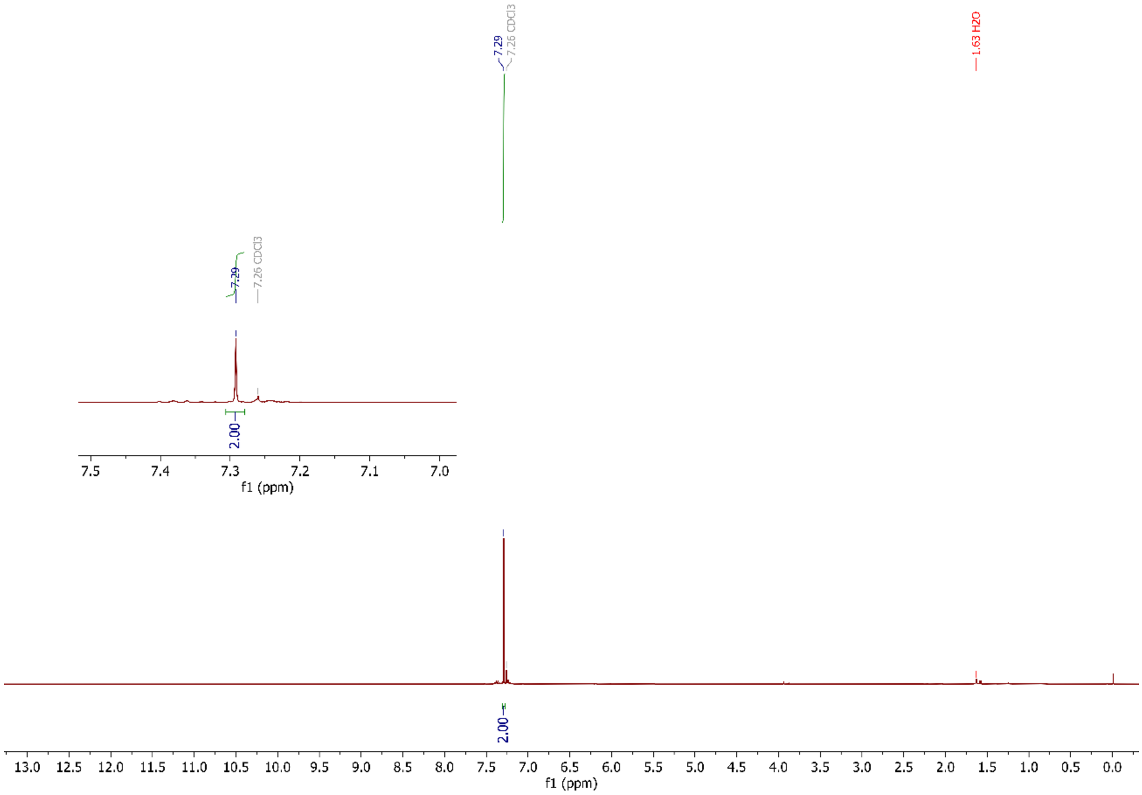

^{13}C NMR (101 MHz, $CDCl_3$) of Furan-2,5-diacyl azide (FDAz)

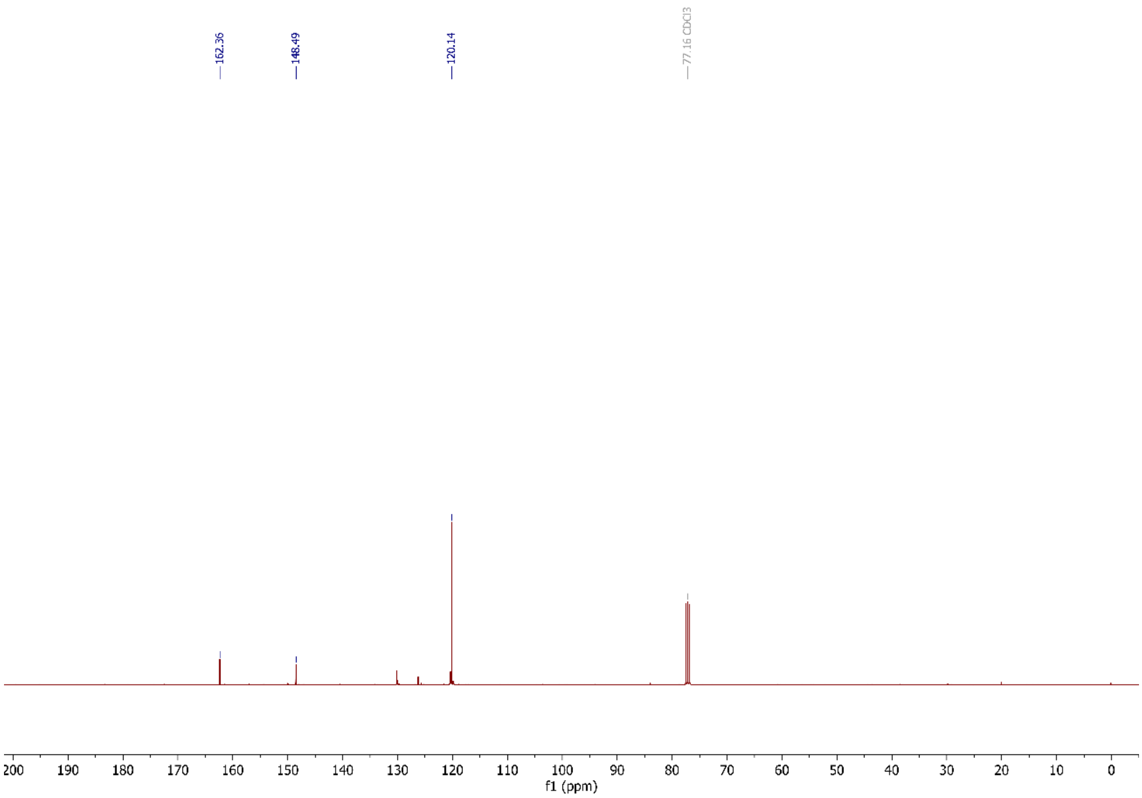

^{1}H NMR (500 MHz, DMSO-d6) Polyurethane (5m)

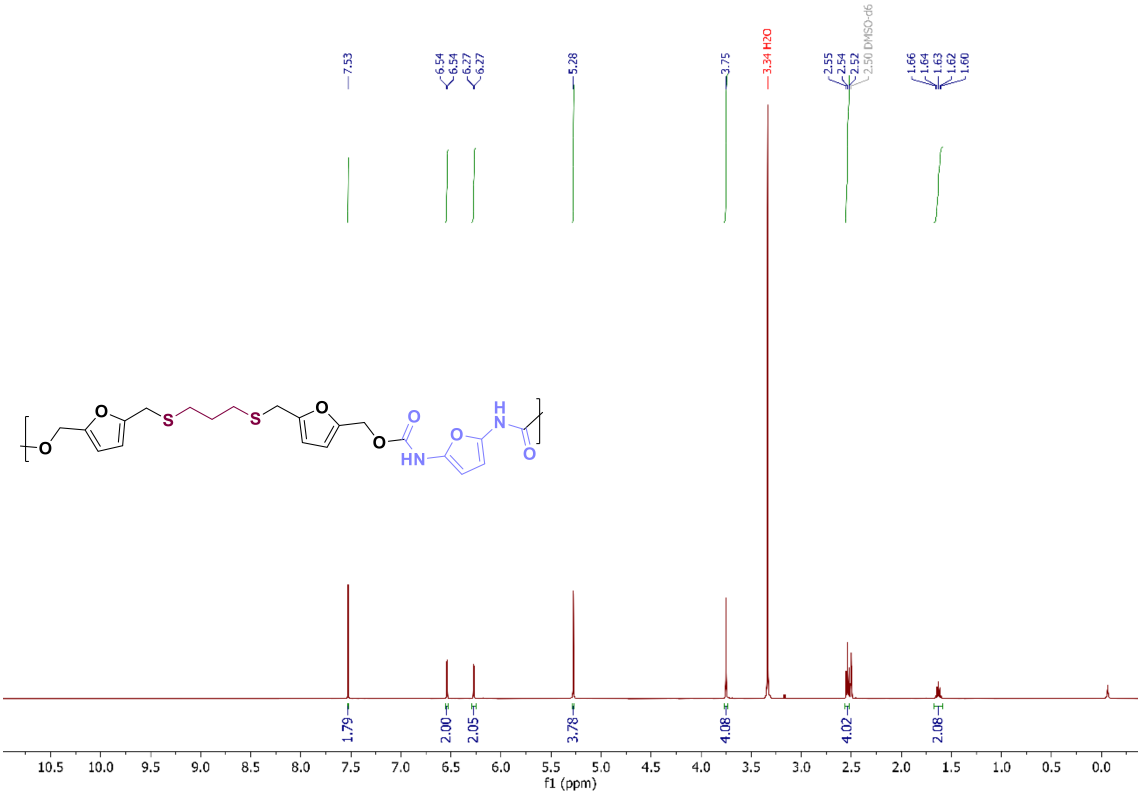

^{13}C NMR (126 MHz, DMSO-d6) Polyurethane (5m)

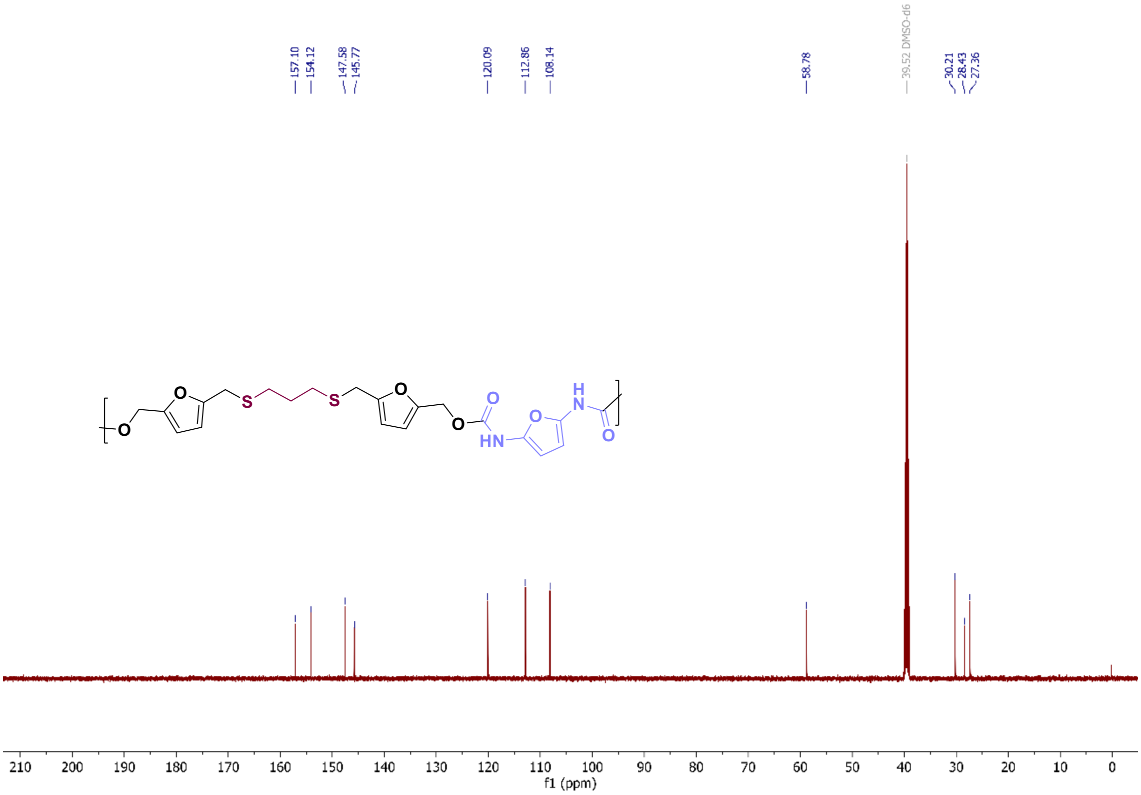

Copy of the IR spectrum

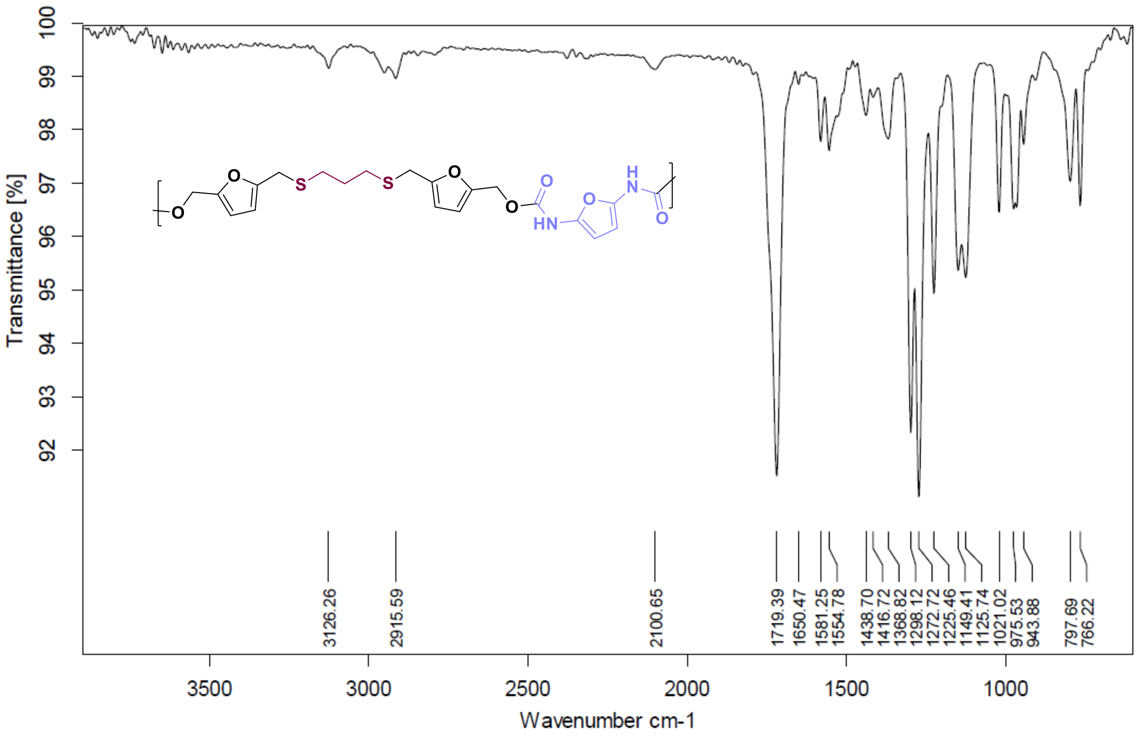

Copy of the TGA curve

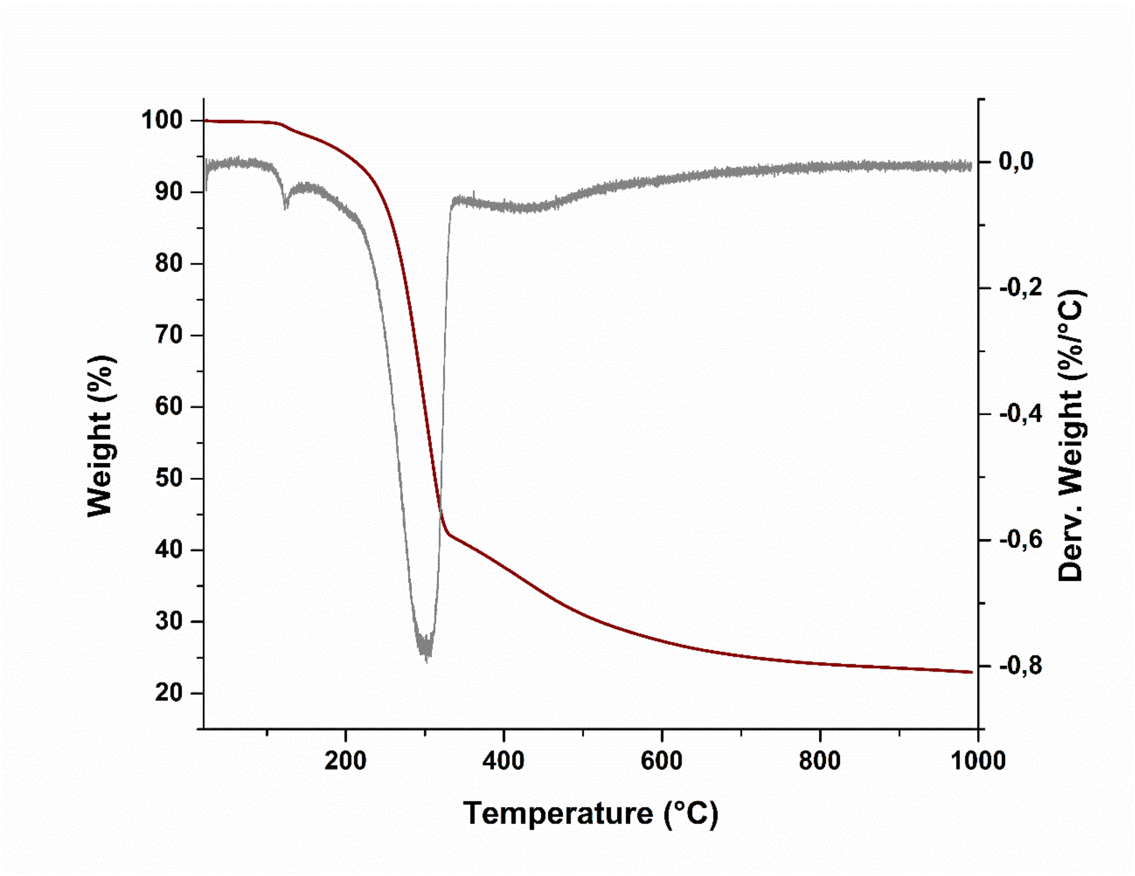

Copies of the Debye plot

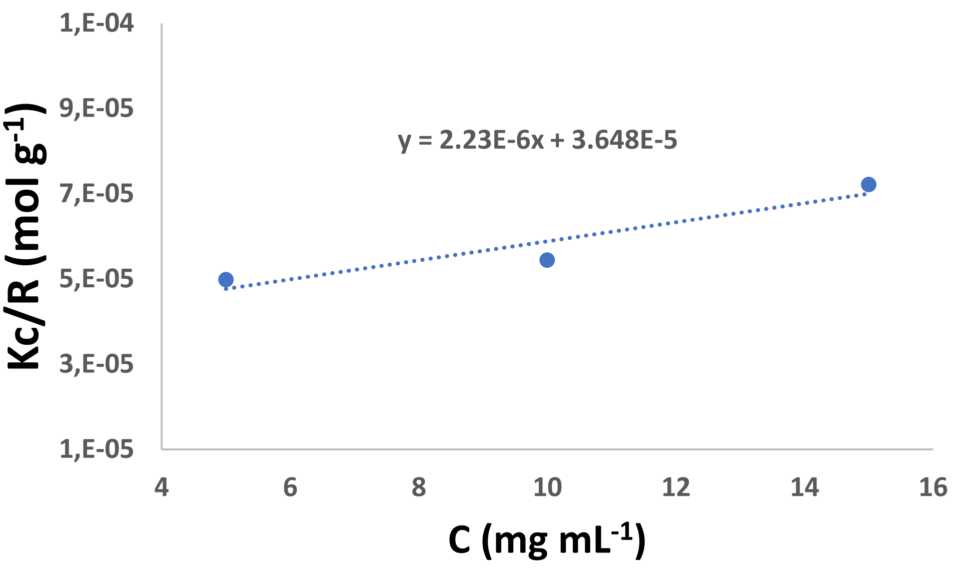

11. **Degradation of polyurethane 5a using p-toluenesulfonic acid.**

A 10 mL round-bottom flask with a distillation column was charged with 100 mg of polyurethane **5a** and 5 mL of methanol. Then, 50 of *p*-toluenesulfonic acid monohydrate was added, and the formed mixture reacted for 4 h at 60°C. Subsequently, the remaining solid was filtered. The obtained organic phase was evaporated under reduced pressure, and the crude material was purified using a chromatography column loaded with silica gel pre-eluted with the mobile phase solvents (EtOAc/Hex 1:9).

1,3-bis(((5-(methoxymethyl)furan-2-yl)methyl)thio)propane (6a) = This product was obtained as a yellow oil (32 mg, 42%) from polyurethane **5a.** ^{1}**H NMR (500 MHz, CDCl$_3$)** δ 6.24 (d, *J* = 3.2 Hz, 2H), 6.14 (d, *J* = 3.2 Hz, 2H), 4.36 (s, 4H), 3.68 (s, 4H), 3.36 (s, 6H), 2.59 (t, *J* = 7.2 Hz, 4H), 1.81 (p, *J* = 7.2 Hz, 2H). ^{13}**C NMR (101 MHz, CDCl$_3$)** δ 152.27, 151.39, 110.39, 108.25, 66.55, 57.99, 30.79, 28.77, 28.53. **IR: ν (neat, ATR)/cm^{-1}** 2923, 2852, 2819, 1678, 1553, 1411, 1250, 1230, 11900, 1087, 1019, 967, 945, 902, 794. **R$_f$** = 0.14 (EtOAc: Hex 1:9). **HMRS (ESI-TOF) m/z: [M + Na]$^+$** calculated for $C_{17}H_{24}O_4S_2$ 379.1008, found 379.1008.

1,2-bis(((5-(methoxymethyl)furan-2-yl)methyl)thio)ethane (6b) = This product was obtained as a yellow oil (31 mg, 40%) from polyurethane **5d.** ^{1}**H NMR (500 MHz, CDCl$_3$)** δ 6.24 (d, *J* = 3.2 Hz, 2H), 6.14 (d, *J* = 3.1 Hz, 2H), 4.35 (s, 4H), 3.72 (d, *J* = 0.7 Hz, 4H), 3.36 (s, 6H), 2.69 (s, 4H). ^{13}**C NMR (126 MHz, CDCl$_3$)** δ 151.9, 151.4, 110.3, 108.3, 66.4, 57.9, 31.5, 28.4. **IR: ν (neat, ATR)/cm^{-1}** 2918, 2850, 2819, 1552, 1463, 1377, 1359, 1258, 1230, 1191, 1178, 1083, 1018, 967, 943, 900, 791. **R$_f$** = 0.12 (EtOAc: Hex 1:9). **HMRS (ESI-TOF) m/z: [M + Na]$^+$** calculated for $C_{16}H_{22}O_4S_2$ 365.0852, found 365.0843.

bis(4-(((5-(methoxymethyl)furan-2-yl)methyl)thio)phenyl)sulfane (6c) = This product was obtained as a yellow oil (22 mg, 33%) from polyurethane **5g.** ^{1}**H NMR (500 MHz, cdcl$_3$) δ** 7.27 – 7.24 (m, 4H), 7.22 – 7.18 (m, 4H), 6.21 (d, *J* = 3.2 Hz, 2H), 6.07 (d, *J* = 3.2 Hz, 2H), 4.34 (s, 4H), 4.07 (s, 4H), 3.34 (s, 6H). ^{13}**C NMR (126 MHz, cdcl$_3$)** δ 151.5, 151.3, 135.0, 134.2, 131.4, 131.2, 110.4, 108.8, 66.5, 58.0, 31.7. **IR: ν (neat, ATR)/cm^{-1}** 2927, 2821, 1777, 1573, 1516, 1179, 1013, 992, 967, 945, 903, 796, 758. **R$_f$** = 0.11 (EtOAc: Hex 1:9). **HMRS (ESI-TOF) m/z: [M + Na]$^+$** calculated for $C_{26}H_{26}O_4S_3$ 521.0885, found 521.0870.

1,12-bis(5-(methoxymethyl)furan-2-yl)-5,8-dioxa-2,11-dithiadodecane (6d) = This product was obtained as a colorless oil (31 mg, 41%) from polyurethane **5j.** **^{1}H NMR (500 MHz, $CDCl_3$)** δ 6.24 (d, *J* = 3.1 Hz, 2H), 6.15 (d, *J* = 3.1 Hz, 2H), 4.35 (s, 4H), 3.75 (s, 4H), 3.66 – 3.56 (m, 9H), 3.36 (s, 6H), 2.71 (t, *J* = 6.7 Hz, 4H). **^{13}C NMR (126 MHz, $CDCl_3$)** δ 152.2, 151.4, 110.3, 108.4, 70.9, 70.4, 66.5, 57.9, 31.1, 28.9. **IR: v (neat, ATR)/cm^{-1}** 2871, 1553, 1450, 1355, 1084, 1019, 994, 967, 944, 793, 759. **R_f =** 0.10 (EtOAc: Hex 1:9). **HMRS (ESI-TOF) m/z: [M + Na]$^+$** calculated for $C_{20}H_{30}O_6S_2$ 453.1376, found 453.1370.

Copies of the RMN spectral of the degraded products

^{1}H NMR (500 MHz, $CDCl_3$) 1,3-bis(((5-(methoxymethyl)furan-2-yl)methyl)thio)propane (6a)

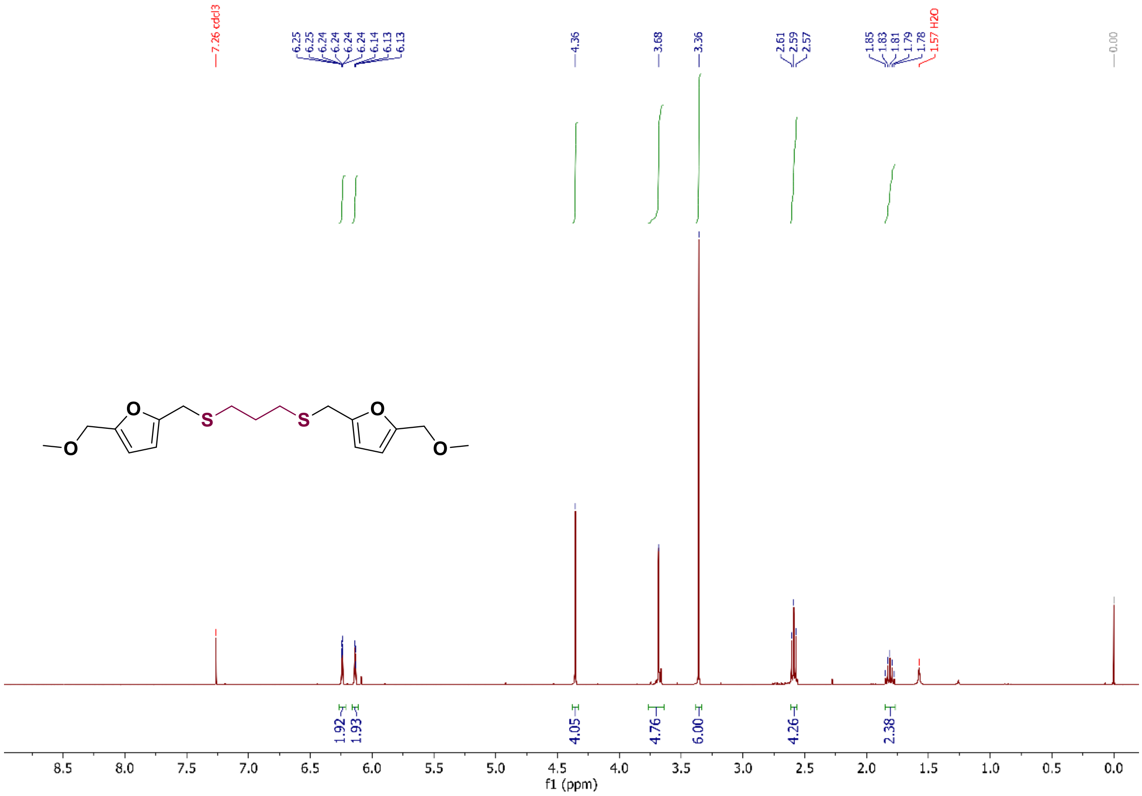

^{13}C NMR (101 MHz, $CDCl_3$) 1,3-bis(((5-(methoxymethyl)furan-2-yl)methyl)thio)propane (6a)

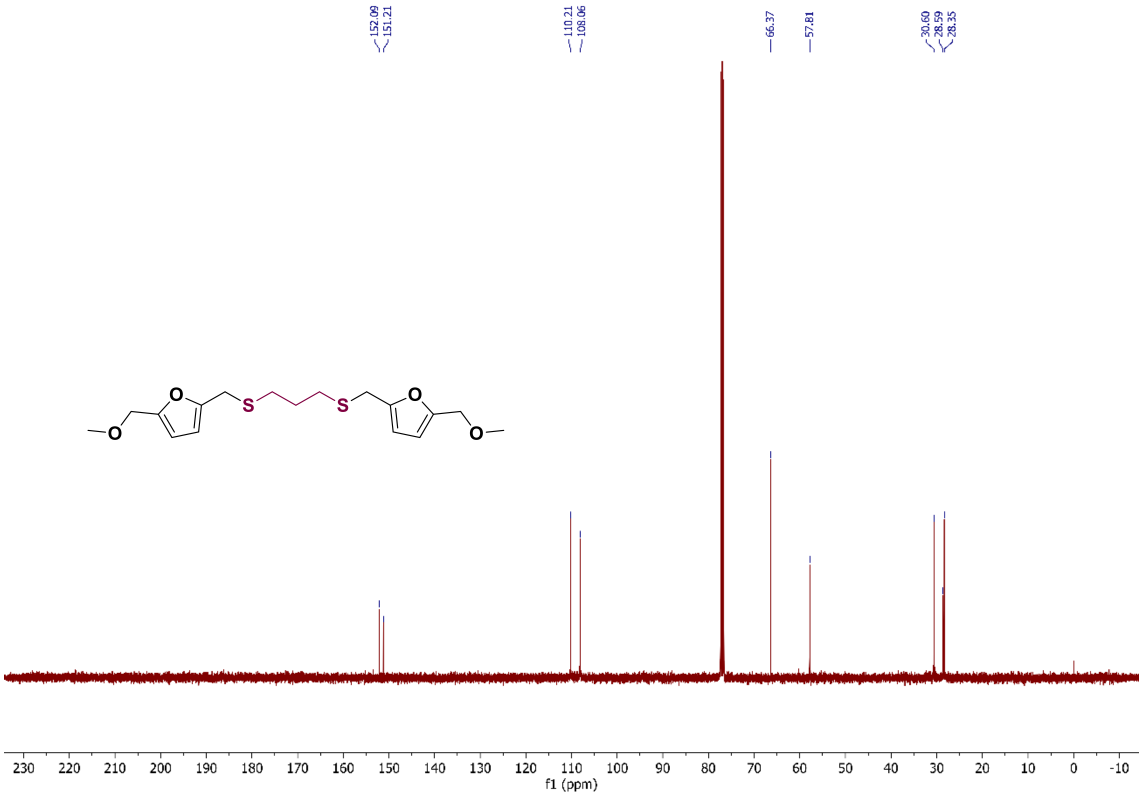

^{1}H NMR (500 MHz, $CDCl_3$) 1,2-bis(((5-(methoxymethyl)furan-2-yl)methyl)thio)ethane (6b)

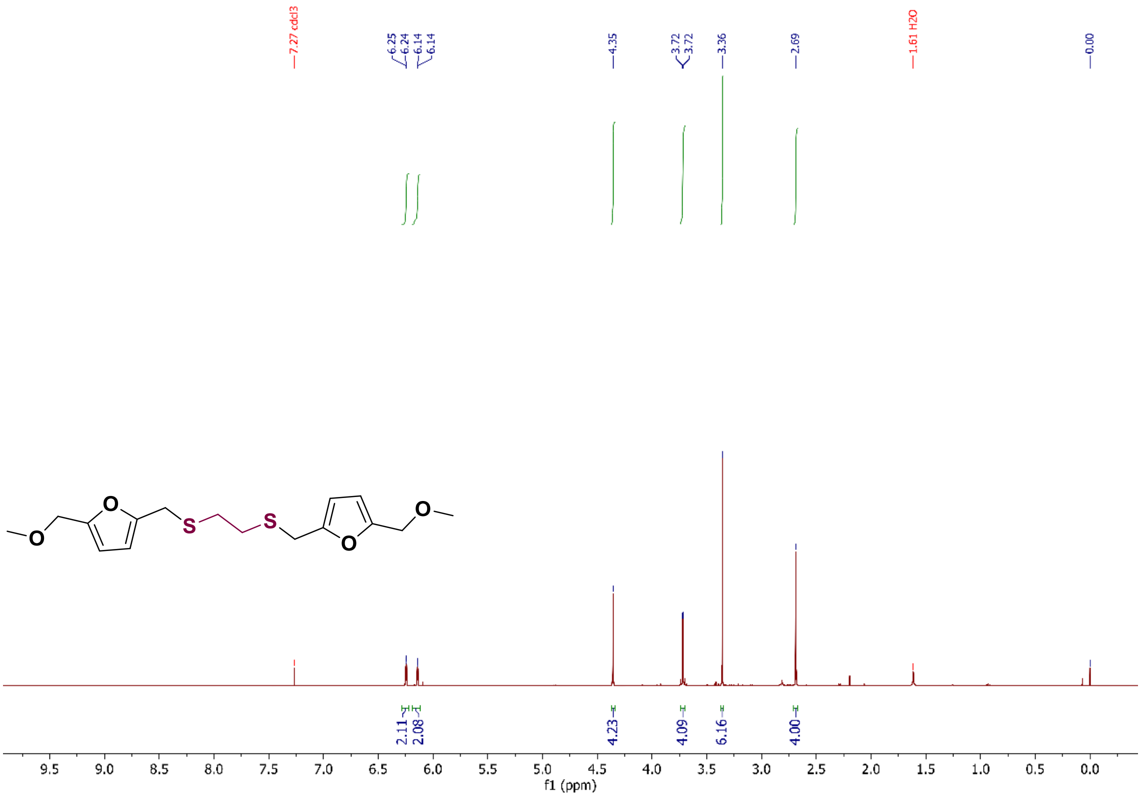

^{13}C NMR (126 MHz, $CDCl_3$) 1,2-bis(((5-(methoxymethyl)furan-2-yl)methyl)thio)ethane (6b)

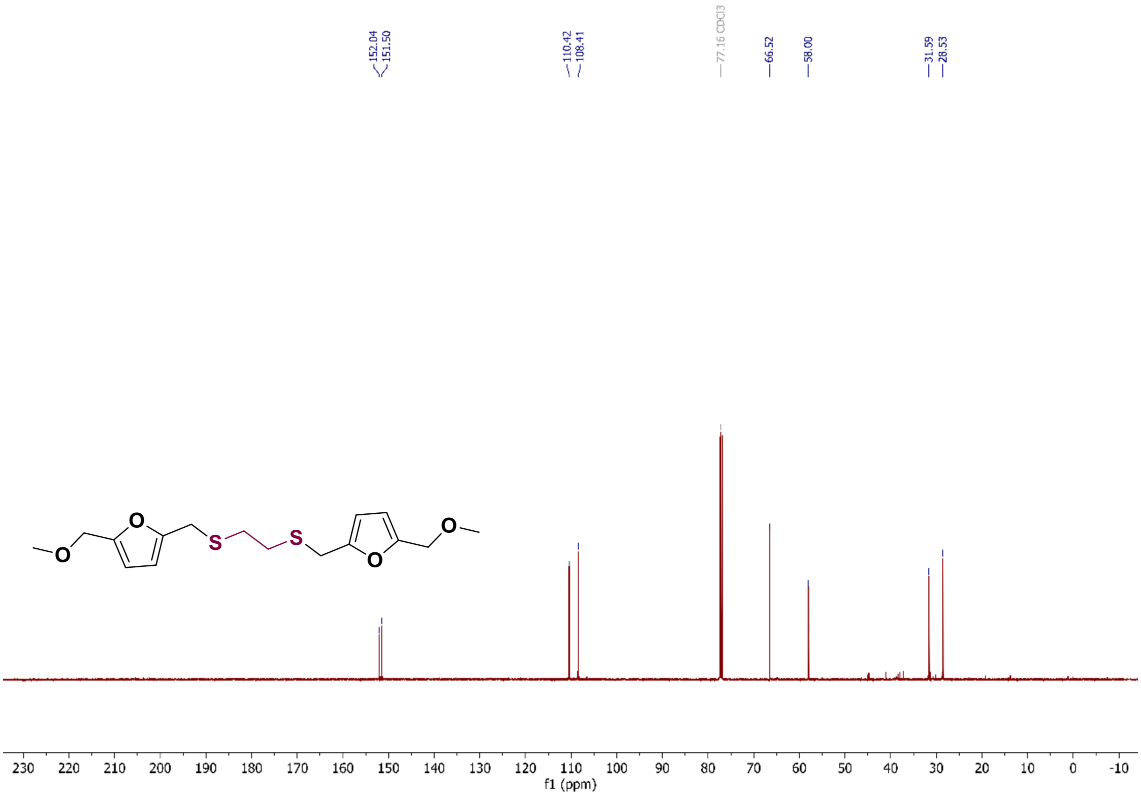

^{1}H NMR (500 MHz, $CDCl_3$) bis(4-(((5-(methoxymethyl)furan-2-yl)methyl)thio)phenyl)sulfane (6c)

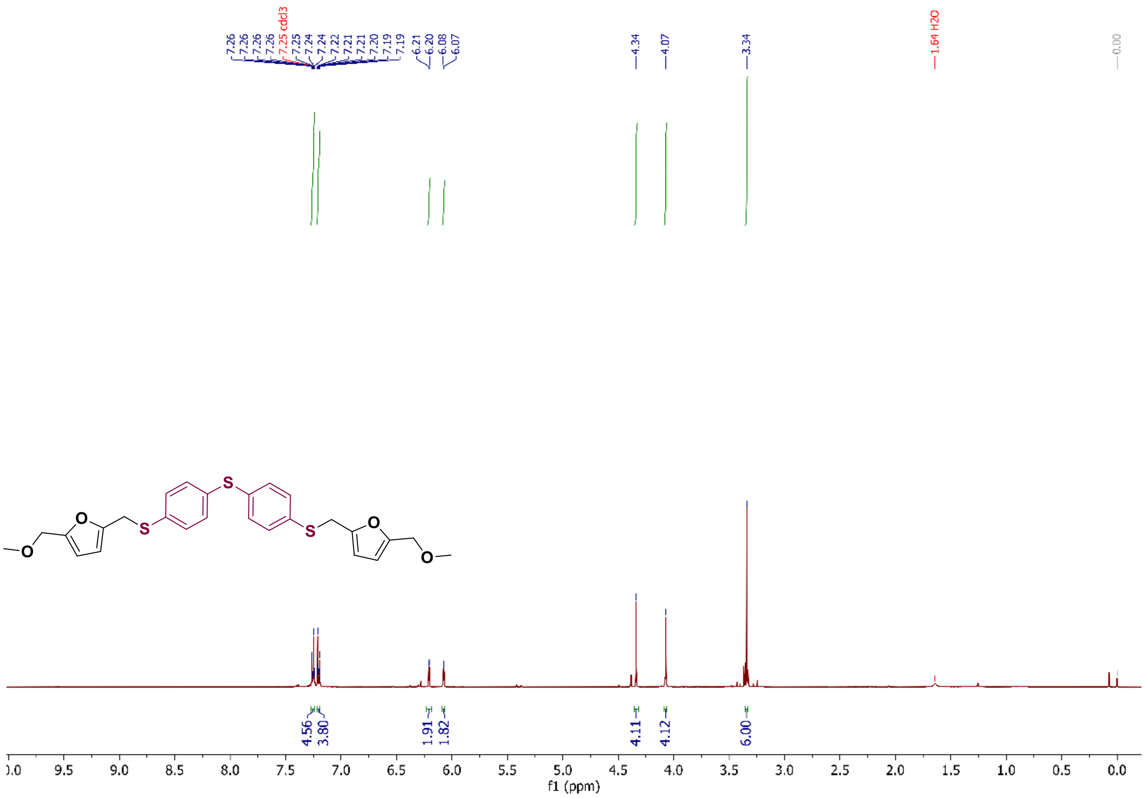

^{13}C NMR (126 MHz, $CDCl_3$) bis(4-(((5-(methoxymethyl)furan-2-yl)methyl)thio)phenyl)sulfane (6c)

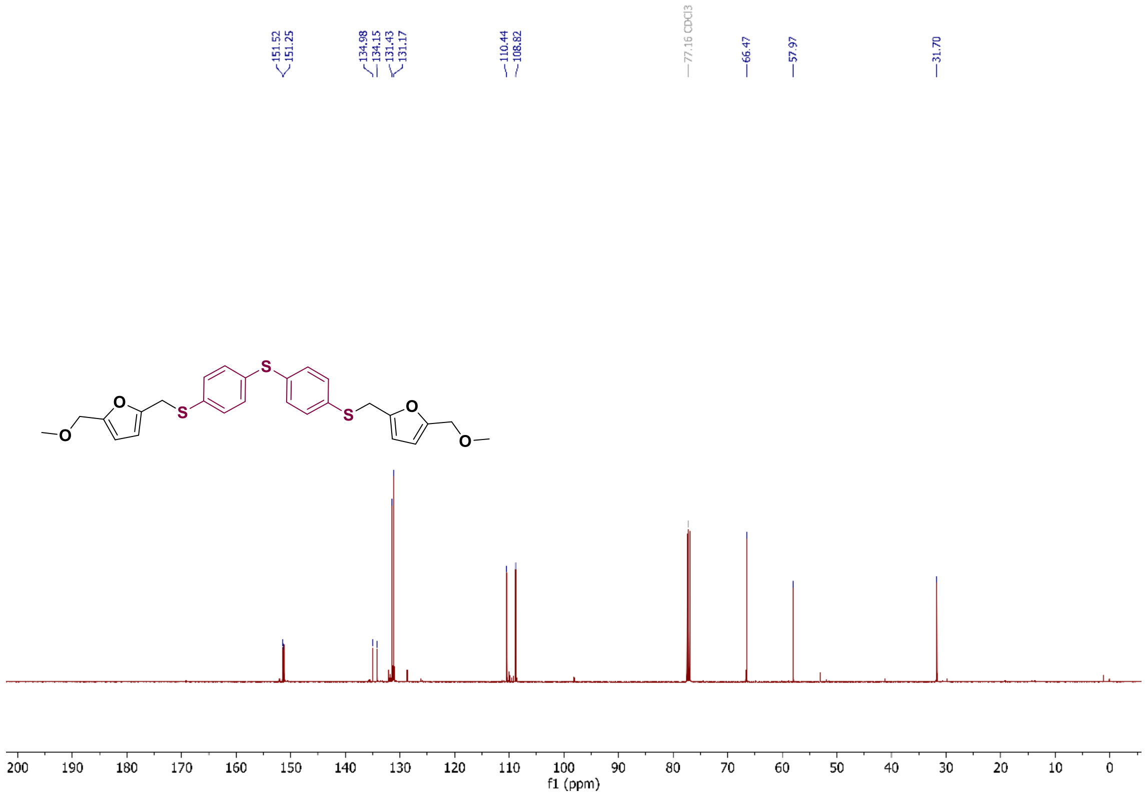

^{1}H NMR (500 MHz, $CDCl_3$) 1,12-bis(5-(methoxymethyl)furan-2-yl)-5,8-dioxa-2,11-dithiadodecane (6d)

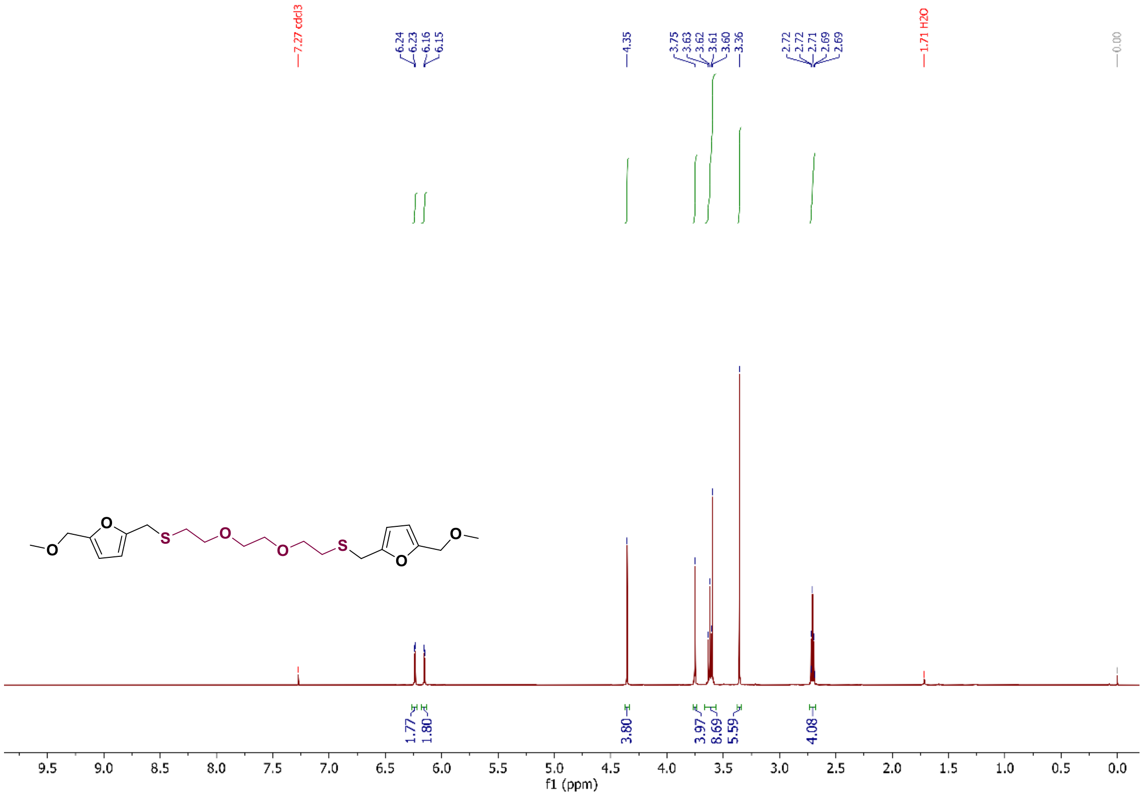

^{13}C NMR (126 MHz, $CDCl_3$) 1,12-bis(5-(methoxymethyl)furan-2-yl)-5,8-dioxa-2,11-dithiadodecane (6d)

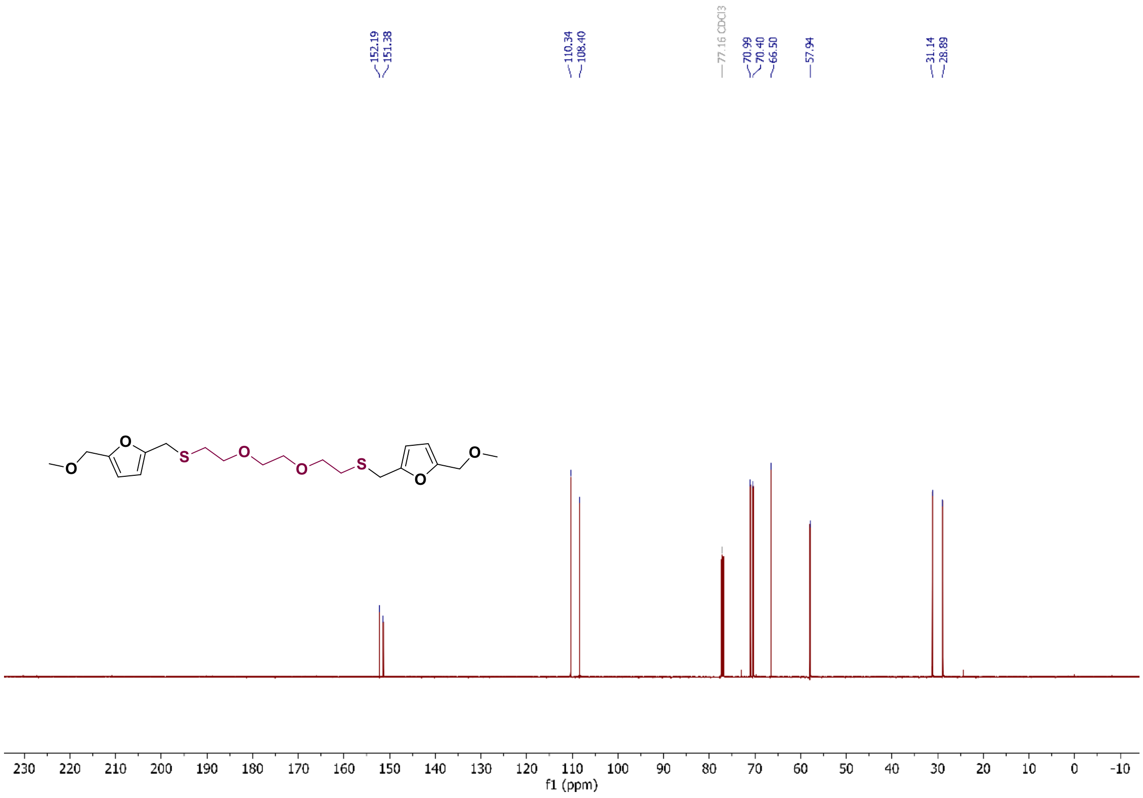

Copy of the ^{1}H RMN (400 MHz, DMSO-d_6) spectrum obtained for the recovered polymer 5a after degradation

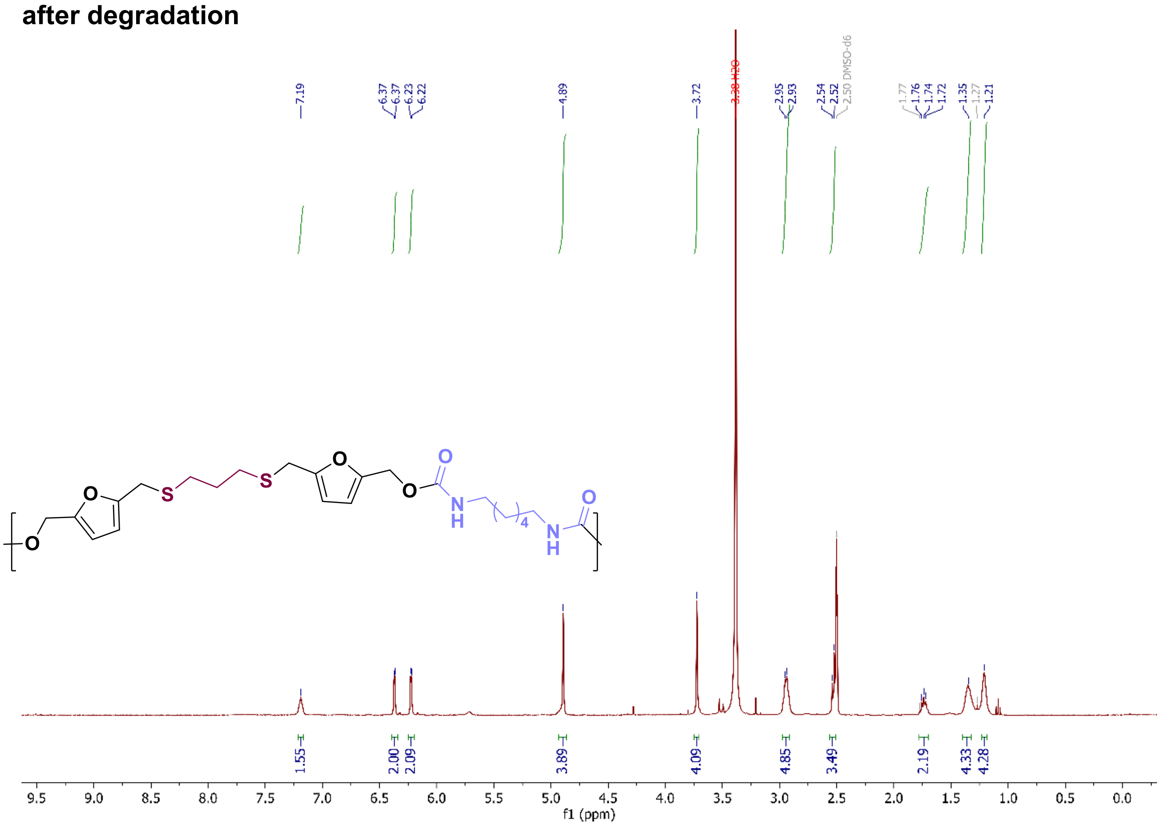

Synthesis and degradation of Polymer 8

Initially, a 50 mL round bottom flask equipped with a distillation column was charged with 5 g of fructose (27.7 mmol) and 25 mL of dimethylsulfoxide (DMSO). The formed solution was heated at 110°C for 6 h. Then, 50 mL of distilled water was added, and the obtained mixture was extracted with ethyl acetate (6 x 50 mL). The organic phases were mixed and washed with a saturated solution of sodium chloride, dried over anhydrous sodium sulfate, and evaporated using reduced pressure providing a crude material, which was purified using a chromatography column loaded with silica gel pre-eluted with the mobile phase solvent (EtOAc/Hex (3:7)). Chromatography afforded the desired HMF as a yellow oil in 78% yield. Subsequently, a 25 mL round bottom flask was charged with 1 mmol of HMF, 1.2 mmol of $NaBH_4$, and 15 mL of ethanol. The formed mixture reacted at room temperature for 2.5 h. Upon completion, 20 mL of water was added and the obtained solution was extracted with ethyl acetate (4 x 20 mL). The organic phases were mixed and washed with a saturated solution of sodium chloride, dried over anhydrous sodium sulfate, and evaporated using reduced pressure providing a crude material, which was purified using a chromatography column loaded with silica gel pre-eluted with the mobile phase solvents (EtOAc/Hex 1:1). Chromatography afforded the desired 2,5-Bis(hydroxymethyl)furan (BHMF) as a white solid in 85% yield.

Finally, a 10 mL round bottom flask with a distillation column was charged with 0.5 mmol of the BHMF, 20 mol% of DBU, and 5 mL of dry THF. Then, 0.5 mmol of hexamethiylene diisocyanate was added. The formed mixture reacted at 40°C for 18 h. Upon completion, methanol was added and a white precipitate was obtained, which was filtered and washed with methanol providing the desired polyurethane **8**. **Polyurethane 8** = ^{1}H NMR (500 MHz, DMSO-d_6) δ 7.23 (s, 2H), 6.43 (s, 2H), 4.92 (s, 4H), 2.99 – 2.91 (m, 4H), 1.38 – 1.30 (m, 4H), 1.22 (d, J = 7.1 Hz, 4H). ^{13}C NMR (126 MHz, DMSO-d_6) δ 158.08, 155.62, 150.68, 111.07, 57.29, 30.00, 29.29, 25.90.

Copy of the ^{1}H RMN (500 MHz, DMSO-d$_6$)

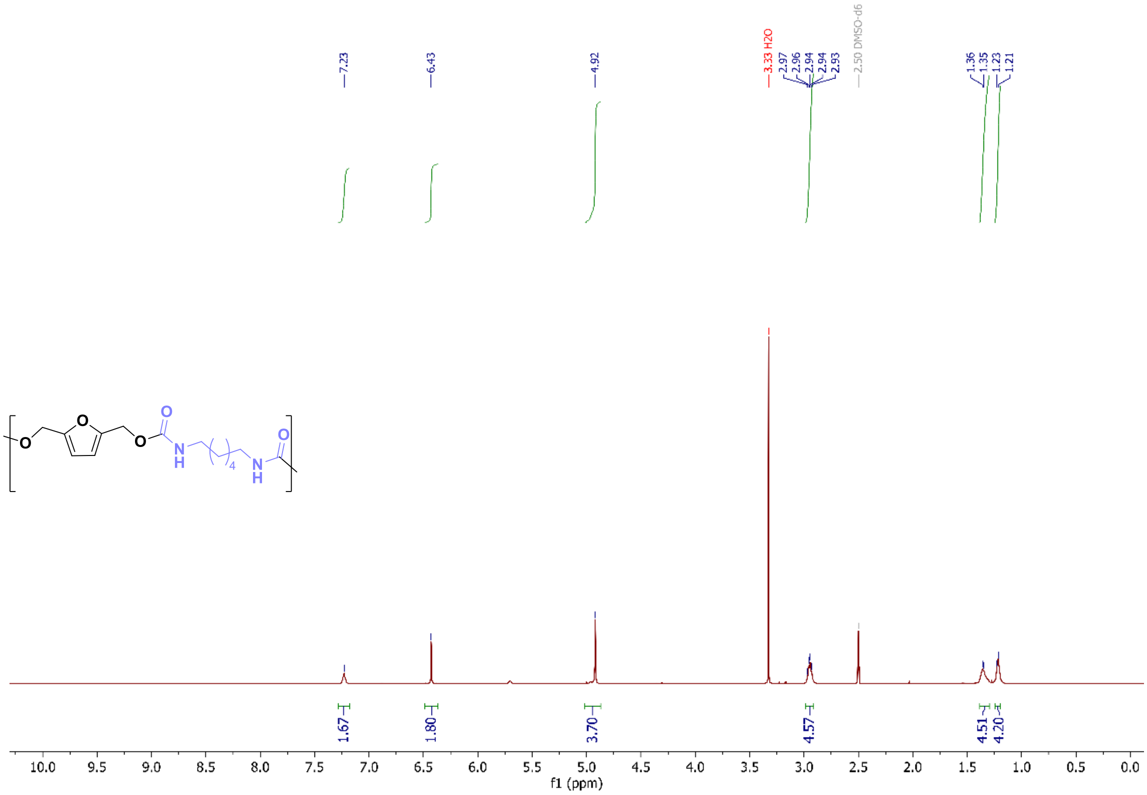

Copy of the ^{13}C RMN (126 MHz, DMSO-d$_6$)

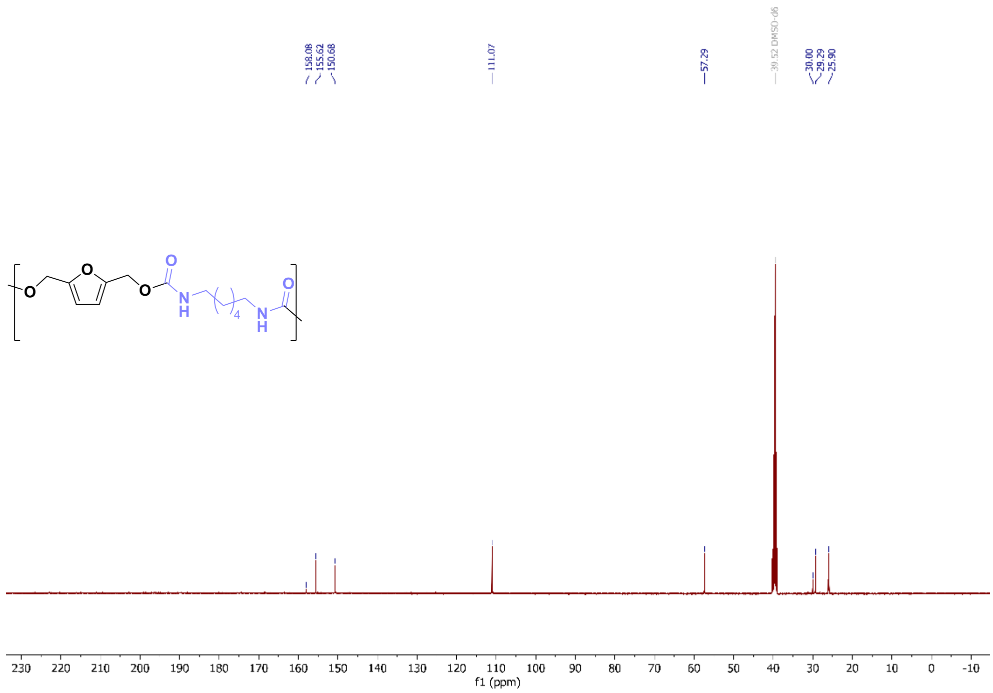

Subsequently, a 10 mL round-bottom flask with a distillation column was charged with 100 mg of polyurethane **8** and 5 mL of methanol. Then, 200 mg of *p*-toluenesulfonic acid monohydrate was added, and the formed mixture reacted for 4 h at 60°C. Subsequently, the remaining solid was filtered, washed with methanol, dried under reduced pressure, and analyzed by ^{1}H NMR.

Copy of the 1H NMR (400 MHz, $CDCl_3$) obtained for the crude material after degradation of polymer 8

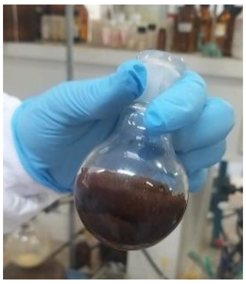

Photography of the obtained crude material after the reaction.

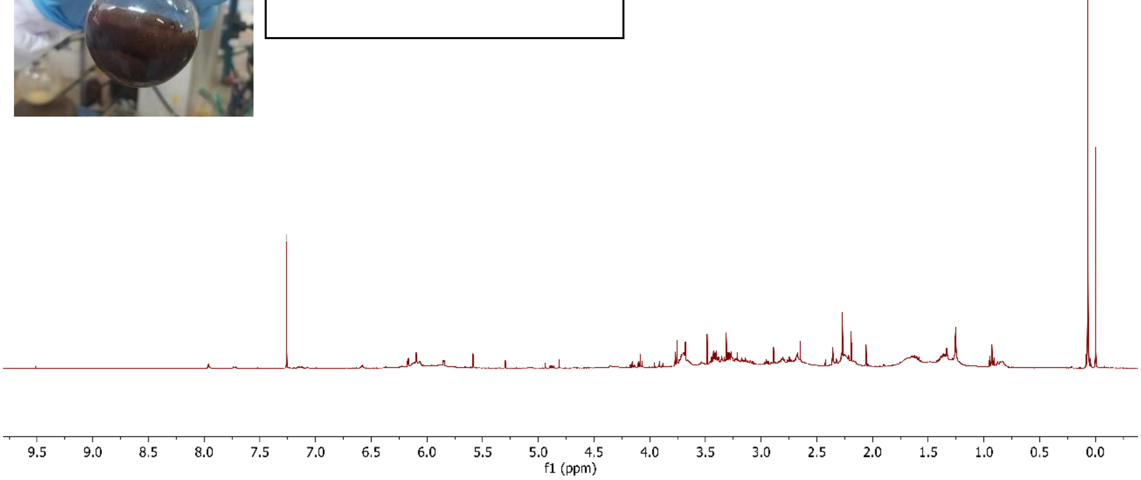

Supplement: Supplementary file 1 — Supplementary Material [file CSSC-18-e202500888-s001.pdf]
